# Structure-Activity Relationships of the Antitumor C_5_-Curcuminoid GO-Y030

**DOI:** 10.3390/molecules200815374

**Published:** 2015-08-24

**Authors:** Aki Kohyama, Hiroyuki Yamakoshi, Shoko Hongo, Naoki Kanoh, Hiroyuki Shibata, Yoshiharu Iwabuchi

**Affiliations:** 1 Graduate School of Pharmaceutical Sciences, Tohoku University, 6-3 Aobayama, Sendai 980-8578, Japan; E-Mails: a-kohyama@dc.tohoku.ac.jp (A.K.); yamakoshi@phar.nagoya-cu.ac.jp (H.Y.); nkanoh@m.tohoku.ac.jp (N.K.); 2 Department of Clinical Oncology, Faculty of Medicine, Akita University, Akita 010-8643, Japan; E-Mails: hongoh23@gmail.com (S.H.); hiroyuki@med.akita-u.ac.jp (H.S.)

**Keywords:** curcumin, antitumor, Michael reaction, structure-activity relationship

## Abstract

1,5-Bis(4-hydroxy-3-methoxyphenyl)-1,4-pentadiene-3-one (**2**) was isolated from *Curcuma domestica* as a curcumin (**1**)-related compound, which we named C_5_-curcumin. Intrigued by the potent antitumor activity of C_5_-curcumin (**2**)-related 1,5-bisaryl-1,4-pentadiene-3-ones [bis(arylmethylidene)acetones, termed C_5_-curcuminoids], we previously conducted a structure–activity relationship study of C_5_-curcuminoids and showed that highly active GO-Y030 [1,5-bis(3,5-bis(methoxymethoxy)phenyl)-1,4-pentadiene-3-one (**4**)] is the most promising antitumor compound. In this study, a panel of C_5_-curcuminoids based on GO-Y030, consisting of 30 new and 10 known compounds, was synthesized to elucidate in detail which moiety of GO-Y030 is significant for antitumor activity. The results confirmed that both the cross-conjugated dienone moiety and the 3,5-bis(methoxymethoxy) substituent are important for the antitumor activity.

## 1. Introduction

**Figure 1:**
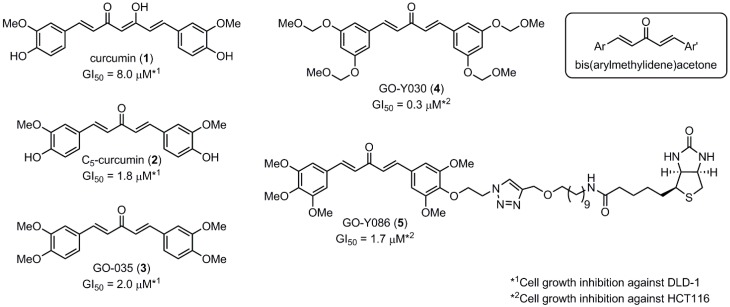
Structures of curcumin and C_5_-curcuminoids.

Despite their unconventional, cross-conjugated dienone structure, bis(arylmethylidene)acetones have spurred numerous studies in medicinal chemistry owing to their capability of eliciting useful biological activities including anti-HIV [1], antioxidant [2,3,4], anti-inflammatory [2,5], and antitumor activities [2,6,7,8,9,10]. Previously, we identified GO-035 (**3**) as a hit antitumor compound while screening our in-house compound library (Figure 1). Our intuition tempted us to view GO-035 as a truncated analogue of curcumin (**1**), the active ingredient of turmeric, which elicits various biological activities without apparent toxicity [11,12]. Indeed, GO-035 corresponds to the 4,4′-*O*-dimethylated analogue of naturally occurring 1,5-bis(4-hydroxy-3-methoxyphenyl)-1,4-pentadiene-3-one (**2**), isolated by Masuda and coworkers from *Curcuma domestica* along with curcumin [13,14], which we named “C_5_-curcumin”. The first-generation structure-activity relationship (SAR) studies focusing on aromatic substituents led us to the identification of GO-Y030 [1,5-bis(3,5-bis(methoxymethoxy)phenyl)-1,4-pentadiene-3-one (**4**)] as a promising candidate for further development. GO-Y030, featuring a 3,5-bis(methoxymethoxy) substituent, exhibits more than 10 times the antitumor activity (GI_50_ = 0.3 μM against human colon carcinoma HCT116 cells) of curcumin [12,15]. In addition, it has been confirmed that GO-Y030 has higher potential to regulate a number of oncoproteins that curcumin interferes with. Specifically, GO-Y030 was shown to downregulate β-catenin, ErbB-2, c-Myc, cyclin D1, and Ki-Ras, and to inhibit NF-κB, PI3K/AKT, JAK/STAT3, and IRF4 pathways [11,16,17]. The promising features of GO-Y030 as an antitumor agent were highlighted by its chemopreventive capability in familial adenomatous polyposis (FAP) mice without apparent *in vivo* toxicity [15].

To shed light on the mode of antitumor action of C_5_-curcuminoid, our group had synthesized the biotinylated probe GO-Y086 (**5**) and identified a binding protein, which is the nuclear protein Far Upstream Element-Binding Protein 2/KH-type Splicing Regulatory Protein (FUBP2/KSRP) [18]. It was indicated that GO-Y086 covalently binds to Cys 500 of FUBP2 via a Michael reaction, resulting in suppression of the expression of the c-Myc protein, which plays an important role in cellular proliferation. Historically, reactive functional groups, such as epoxides and α,β-unsaturated carbonyl groups have been regarded as risk factors in medicinal chemistry [19,20,21]. In recent years, however, exceptional examples have proven that covalent-bond-forming compounds can afford promising results in drug development. Very recently, Mahlknecht and coworkers disclosed that C_5_-curcumin (**2**), showing superior antitumor activity to curcumin, is formed by pyrolyzing curcumin with coconut fat, suggesting that cooking enhances the anticancerogenic activity of curcumin [22]. Yang and coworkers also reported that C_5_-curcuminoid has better pharmacokinetic characteristics than curcumin [9]. As such, C_5_-curcuminoids are emerging as intriguing bioactive compounds in current medicinal chemistry. Hence, we decided to conduct a SAR study focusing on GO-Y030 to gain insight into the importance in exerting the cytotoxic properties of: (i) the methoxymethyl substituent; (ii) the cross-conjugated dienone moiety; and (iii) the two phenyl rings. We also explored the possibility of increasing the cytotoxicity of C_5_-curcuminoids by modulating the stereoelectronic properties of the cross-conjugated dienone moiety.

## 2. Results and Discussion

### 2.1. Chemistry

**Scheme 1:**
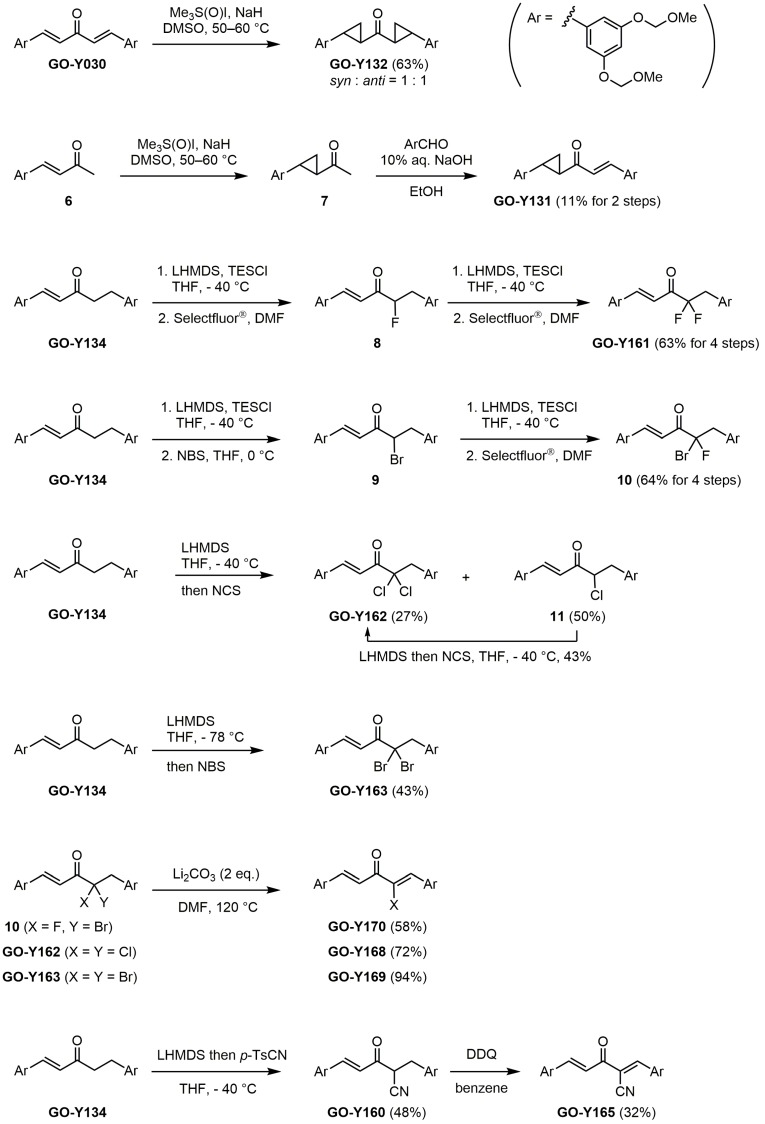
Synthesis of cyclopropanated analogues and α-halo and α-cyano analogues.

δ-Symmetric 1,5-diaryl-3-oxo-1,4-pentadienes were synthesized by the aldol condensation of benzaldehydes with acetone under basic conditions using NaOH as catalyst. The bis-cyclopropane analogue GO-Y132 was obtained in one operation from GO-Y030 as a diastereomeric mixture by the Corey-Chaykovsky reaction (Scheme 1) [23]. The monocyclopropane analogue was synthesized from enone **6** in two steps. The α-halogenated dienone analogues GO-Y168–GO-Y170 were synthesized from dihalogenated analogues **10**, GO-Y162 and GO-Y163, by elimination of HX [24,25]. GO-Y165 was prepared from GO-Y134 by cyanation using LHMDS and *p*-TsCN, followed by DDQ oxidation [26]. The (*E*)-β-(3,5-bis(methoxymethoxy)phenyl)-α,β-unsaturated carbonyl compounds GO-Y148–GO-Y151, GO-Y157, and GO-Y171 were synthesized by transformation of a homologous aldehyde [see Supporting Information (SI) Scheme S1].

### 2.2. Pharmacological Evaluation

The antitumor activities of the synthesized compounds were evaluated in terms of the viability of human colon cancer line HCT-116 cells with the quantitation of the uptake and digestion of 2-(2-methoxy-4-nitrophenyl)-3-(4-nitrophenyl)-5-(2,4-disulfophenyl)-2*H*-tetrazolium monosodium salt in accordance with the manufacturer’s instructions (see Section 3).

### 2.3. SAR of C_5_-Curcuminoid GO-Y030

#### 2.3.1. Roles of the 3,5-Bismethoxymethoxy Substituent

Considering the inherent acid sensitivity of the methoxymethoxy group and its potential to release toxic formaldehyde, the cytotoxic effect of the 3,5-bis(methoxymethoxy) substituent analogue was investigated. Theoretically, one equiv. of GO-Y030 could produce one equiv. of GO-Y038 and four equiv. of formaldehyde via acid hydrolysis.

Therefore, GO-Y129 and GO-Y130, which do not produce formaldehyde under acidic conditions, were synthesized for comparison. As shown in Table 1, GO-Y129 exhibited comparable activity to GO-Y030, and GO-Y147 exhibited a decreased but still high cytotoxicity. To enhance water solubility, GO-Y130, carrying four hydroxyl groups on the aromatic 3,5-substitutent termini, was synthesized. Unfortunately, its cytotoxicity decreased. GO-Y164, the 2,5-substituted bis(arylmethylidene)acetone, showed a slightly attenuated activity, underscoring the more productive effect of 3,5-substitution. GO-Y156, a hybrid analogue of the most potent GO-Y030 and the inactive GO-949, showed an intermediate cytotoxicity as expected. Regarding the effect of 3,5-bis(methoxymethoxy) substitution, it was suggested that it is necessary for antitumor activity but does not function as a formaldehyde source.

**Table 1:** SAR of 3,5-bis(methoxymethoxy) substitution. 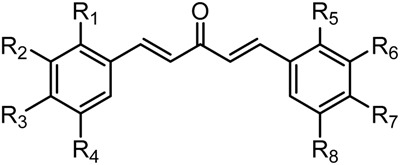

Compound	R		GI_50_ (μM) *^2^
GO-949	R_2_ = R_3_ = OCH_2_O, R_6_ = R_7_ = OCH_2_O	R_1_ = R_4_ = R_5_ = R_8_ = H	>50 *^1^
GO-Y030	R_2_ = R_4_ = R_6_ = R_8_ = OCH_2_OMe	R_1_ = R_3_ = R_5_ = R_7_ = H	0.3
GO-Y038	R_2_ = R_4_ = R_6_ = R_8_ = OH	R_1_ = R_3_ = R_5_ = R_7_ = H	1.5
GO-Y067	R_2_ = R_4_ = R_6_ = R_8_ = OMe	R_1_ = R_3_ = R_5_ = R_7_ = H	2.0
GO-Y129	R_2_ = R_4_ = R_6_ = R_8_ = OCH_2_CH_2_OMe	R_1_ = R_3_ = R_5_ = R_7_ = H	0.2
GO-Y130	R_2_ = R_4_ = R_6_ = R_8_ = OCH_2_CH_2_OH	R_1_ = R_3_ = R_5_ = R_7_ = H	6.4
GO-Y147	R_2_ = R_4_ = R_6_ = R_8_ = OCH_2_OCH_2_CH_2_OMe	R_1_ = R_3_ = R_5_ = R_7_ = H	2.0
GO-Y156	R_2_ = R_4_ = OCH_2_OMe, R_6_ = R_7_ = OCH_2_O	R_1_ = R_3_ = R_5_ = R_8_ = H	7.9
GO-Y164	R_1_ = R_4_ = R_5_ = R_8_ = OCH_2_OMe	R_2_ = R_3_ = R_6_ = R_7_ = H	1.7

*^1^ Cell growth inhibition against DLD-1. *^2^ Cell growth inhibition against HCT116 except for GO-949.

#### 2.3.2. Roles of the Central Tether Moiety

To analyze in detail the roles of the cross-conjugated dienone moiety in the cytotoxicity, the following analogues were synthesized: *EZ*-isomer (GO-Y095), thiol-adduct analogues (GO-Y077 and GO-Y075), cyclopropanated analogues (GO-Y131 and GO-Y132), and reduced analogues (GO-Y133 and GO-Y134) (Table 2). In addition, in light of the accumulated knowledge of toxic natural products that elicit biological functions via covalent bond formation with a cysteine residue of functional proteins [27,28], we expected that cyclopropanated analogues of GO-Y030, namely, GO-Y131 and GO-Y132, would exhibit higher antitumor activity via irreversible bond formation with particular biothiols. Their cytotoxicities were compared with that of GO-Y030. The *EZ*-isomer GO-Y095 showed a comparable activity to GO-Y030, indicating that the stereochemistry of the double bond is not important for biological activity. This particular result indicates that C_5_-curcuminoids undergo a reversible Michael-retro Michael reaction with a biogenic nucleophile [29], which cancels out the initial stereochemistry. Indeed, the monothiol adduct GO-Y077 exhibited a comparable cytotoxicity to GO-Y030, whereas the bisthiol adduct GO-Y075 showed a decreased but still significant antitumor activity. The monoenone-type analogues GO-Y131 and GO-Y134 exhibited a lower activity than GO-Y030. Unfortunately, the cyclopropane analogues GO-Y131 and GO-Y132 exhibited a lower activity than GO-Y030. The monocyclopropane analogue GO-Y131 exhibited a higher activity than GO-Y134. Note that both biscyclopropane analogue GO-Y132 and saturated ketone GO-Y133 completely lost their activity, indicating that the α,β-unsaturated carbonyl moiety is essential for antitumor activity. To summarize, these results clearly indicate that the central dienone moiety constitutes the pharmacophore in which both aromatic rings function as tuning moieties.

**Table 2:** SAR of central tether moiety. 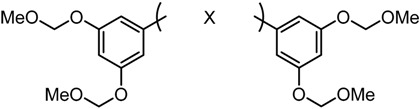

X Compound GI_50_ (μM) *^2^	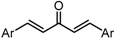	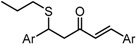	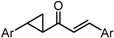	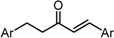
	GO-Y030	GO-Y077	GO-Y131	GO-Y134
	0.3	0.9	6.8	18
X Compound GI_50_ (μM) *^2^	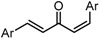	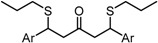	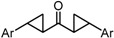	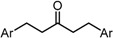
	GO-Y095	GO-Y075	GO-Y132	GO-Y133
	0.9	2.1	>50	>50

*^2^ Cell growth inhibition against HCT116.

#### 2.3.3. Effect of Two Aromatic Rings

To examine the role of the α,β-unsaturated carbonyl moiety as well as to estimate the impact of the two aromatic rings on the antitumor activity of C_5_-curcuminoids, we synthesized the (*E*)-β-(3,5-bis(methoxymethoxy)phenyl)-α,β-unsaturated carbonyl compounds GO-Y148, GO-Y149, GO-Y150, GO-Y151, GO-Y157, and GO-Y171. As shown in Table 3, most of these analogues did not show any significant cytotoxicity, showcasing again the great importance of the cross-conjugated dienone structure. To further investigate the role of the cross-conjugated dienone structure, GO-Y154, an amide analogue bearing two aromatic rings, was synthesized and its cytotoxicity was evaluated. It was found that GO-Y154 was also inactive, which indicates that both the reactivity of the enone and the presence of two aromatic rings are important for cytotoxicity. Next, GO-Y158 and GO-Y159 were synthesized to evaluate the effect of the length of the linker tethering the two aromatic rings. Unlike in the case of the previously reported analogue GO-Y025 [12], GO-Y159, having a C7 linker identical to that of curcumin, was inactive, whereas GO-Y158 and GO-Y025 exhibited reasonable antitumor activity. Judging from the results of curcumin analogues containing two aromatic rings with 3,5-bis(methoxymethoxy) substituents, the appropriate length of central linker for antitumor activity was confirmed to be C_5_.

Table 3SAR of α,β-unsaturated carbonyl: different reactivities and lengths. (**a**) (*E*)-β-(3,5-Bis(methoxymethoxy)phenyl)-α,β-unsaturated carbonyl compounds; (**b**) Central tether moiety.

**Table:** **(a)** 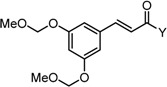

Compound	Y	GI_50_ (μM) *^2^
GO-Y148	OMe	>50
GO-Y149	H	49
GO-Y150	SCH_2_CH_2_CH_3_	>50
GO-Y151	NHMe	>50
GO-Y157	OH	>50
GO-Y171	C(CH_3_)_3_	36

*^2^ Cell growth inhibition against HCT116.

**Table:** **(b)** 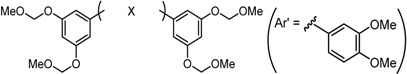

Compound	X	GI_50_ (μM) *^2^
GO-Y154	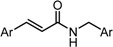	>50
GO-Y158	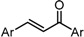	7.3
GO-Y159	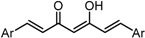	>50
GO-Y025	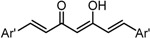	2.0

*^2^ Cell growth inhibition against HCT116.

#### 2.3.4. Effects of Introduction of Electron Withdrawing Group and Amino Group at C-2

Several thought-provoking lessons have been reported in synthetic exploitations of pharmaceutical candidates that feature an α,β-unsaturated carbonyl moiety. In the development of the anticancer drug candidates neratinib and pelitinib, which are known to bind to Cys-773 (or Cys-797) and Cys-805 in EGFR and HER2, Wissner and Mansour proposed that the amino group at the γ-position to the carbonyl group of an α,β-unsaturated carbonyl moiety plays a role as a base for deprotonation of the cysteine thiol of the target protein, and is effective for binding to the target protein [30]. Importantly, Snyder and coworkers developed the promising antitumor agent EF-24 [6,7], which shares a cross-conjugated dienone structure with GO-Y030, but is distinguished by the piperidine ring and aromatic substituent. Therefore, we designed GO-Y166 and GO-Y167, in which an amino methyl group was introduced at the α-position to the carbonyl group; we classified them as type 1 analogues. To assess the impact of the α-aminomethyl moiety of GO-Y166 and GO-Y167, EF-24, GO-Y152, and GO-Y153 were synthesized. On another front, in 1998, Sporn and coworkers reported that introduction of a cyano group as an electron-withdrawing group (EWG) at the α-position of an α,β-unsaturated carbonyl moiety is effective for increasing inhibitory activity [31]. Recently, Taunton [29] and Amslinger [32] independently demonstrated in their research on RSK inhibitors and chalcone derivatives that tuning the reactivity of the Michael acceptor moiety is a useful strategy for increasing the pharmacological activity. As such, we designed GO-Y165, GO-Y168–GO-Y170, and GO-Y172, in which cyano and halogen groups (F, Cl, Br) were introduced at the α-position to the carbonyl group to determine the correlation between the reactivity of the dienone group and cytotoxicity; we classified them as type 2 analogues. As shown in Table 4, type 1 analogues did not exhibit increased cytotoxicity compared with GO-Y030 but were superior to the cyclohexanone analogues GO-Y152 and GO-Y153. The α-cyano analogues GO-Y160 and GO-Y165 were inactive, which is consistent with previous reports on SAR of biologically active chalcones [32]. Type 2 analogues having an additional EWG on the cross-conjugated dienone moiety of GO-Y030 also did not give rise to any positive effect for antitumor activity. From the results shown in Table 5, the electron-withdrawing effects were indicated by the down-field chemical shift of β-hydrogen. Although no promising compounds in terms of antitumor activity were obtained from the type 2 panel, we observed an interesting α-halogen effect in the α,β-unsaturated carbonyl system: although the chlorinated analogue GO-Y168 and the brominated analogues GO-Y169 and GO-Y172 exhibited comparable antitumor activity to GO-Y030, the fluorinated analogue GO-Y170 showed a decreased activity, which can be rationalized by the resonance effect of fluoride. Primarily, halogens play the electron-withdrawing role via the σ-inductive effect, in which the most electronegative fluorine exhibits a pronounced effect. On the other hand, the fluorine atom exerts an electron donating effect via 2*p*-2*p* orbital overlap, which is reflected in the order of the chemical shift of Cβ-H (Br > Cl > H > F).

**Table 4:** SAR of the central tether moiety: introduction of electron-withdrawing group and amine.

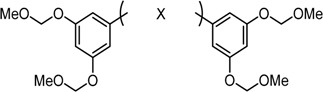			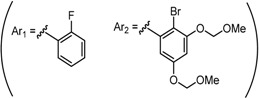		
***Type 1 Analogues***						
X Compound GI_50_ (μM) *^2^	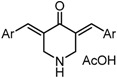	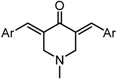	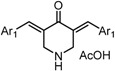	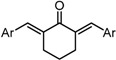	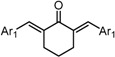
	GO-Y166	GO-Y167	EF-24	GO-Y152	GO-Y153
	1.9	2.8	2.3	3.9	18
X Compound GI_50_ (μM) *^2^	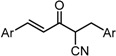	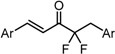	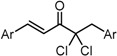	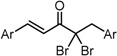	
	GO-Y160	GO-Y161	GO-Y162	GO-Y163	
	>50	>50	19	3.6	
***Type 2 Analogues***						
X Compound GI_50_ (μM) *^2^	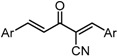	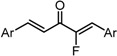	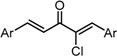	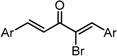	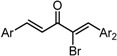
	GO-Y165	GO-Y170	GO-Y168	GO-Y169	GO-Y172
	33	3.6	1.0	1.8	1.3

*^2^ Cell growth inhibition against HCT116.

**Table 5:** Proton chemical shifts of Cβ-H. 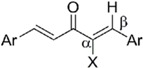

Compound	X	Chemical Shift of Cβ-H (ppm)	GI_50_ (μM) *^2^
GO-Y030	H	7.64	0.3
**GO-Y165**	CN	8.22	3.3
**GO-Y170**	F	6.89	3.6
**GO-Y168**	Cl	7.77	1.0
**GO-Y169**	Br	7.99	1.8

*^2^ Cell growth inhibition against HCT116.

## 3. Experimental Section

### 3.1. General Information

All melting points were determined with a Micro Melting Point BY-2 melting point apparatus (Yazawa, Tokyo, Japan) and are uncorrected. Infrared spectra were obtained on a *FT*/*IR*-410 Fourier Transform Infrared Spectrophotometer (JASCO, Tokyo, Japan) at a 4.0 cm^−1^ resolution and reported in wave numbers. ^1^H-NMR and ^13^C-NMR spectra were recorded using JNM-AL-400 (400/100 MHz), and JNM-ECA-600 (600/150 MHz) spectrometers (JEOL, Tokyo, Japan). ^1^H chemical shifts (δ) are reported in parts per million (ppm) downfield, relative to tetramethylsilane (TMS). Coupling constants (*J*) are reported in Hertz. Multiplicities are reported using the following abbreviations: s, singlet; d, doublet; t, triplet; q, quartet; br, broad. ^13^C chemical shifts are reported in ppm relative to the center of the CDCl_3_ or DMSO-*d*_6_ signals. Low- and high-resolution mass spectra were recorded on a JEOL JMS-DX303 or JMS-700 instrument using electron impact (EI). FAB mass spectra were recorded on a JEOL-JMS700 spectrometer. 

### 3.2. Chemical Synthesis

The synthesis and spectral properties of compounds GO-949, GO-Y030, GO-Y038, GO-Y067, GO-Y075, GO-Y077, and GO-Y095 were reported in our previous papers [11,12]. EF-24 [6,7] and GO-Y153 [33] are known compounds, and were prepared by the reported procedures. The synthesis schemes for GOY133, GO-Y134, GO-Y148–GO-Y151, GO-Y154, GO-Y155, GO-Y157–GO-Y159, GO-Y166, and GO-Y167 and characterization data other than GO-Y compounds are provided in the Supplementary Data of this paper. All reactions were carried out under an atmosphere of argon unless otherwise specified. Anhydrous solvents were transferred via a syringe to flame-dried glassware, which had been cooled under a stream of dry nitrogen. Ethereal solvents and dichloromethane (anhydrous; Kanto Chemical Co., Inc., Tokyo, Japan) were used as received. All other solvents were dried and distilled by standard procedures. Reactions were monitored by thin-layer chromatography (TLC) carried out on silica gel plates (Merck Kieselgel 60 F_254_; Fuji Silysia Chemical, Ltd., Research Triangle Park, NC, USA, NH TLC plates). Column chromatography was performed on Silica gel 60N (Kanto Chemical Co. Inc., spherical, neutral, 63–210 μm) or Cromatorex^®^ NH-DM1020 (Fuji Silysia Chemical, Aichi, Japan; aminopropyl-modified type, 75–150 μm), and flash column chromatography was performed on Silica gel 60N (Kanto Chemical Co. Inc.; spherical, neutral, 40–50 μm). Yields refer to chromatographically and spectroscopically (^1^H-NMR) homogeneous materials unless otherwise stated. Reagents of the highest commercial quality were purchased and used without further purification.

*(1E,4E)-1,5-Bis(3,5-bis(2-methoxyethoxy)phenyl)penta-1,4-dien-3-one* (GO-Y-129). Yellow solid; mp 85–87 °C. IR (CHCl_3_): 2927, 1652, 1590, 1441 cm^−1^; ^1^H-NMR (400 MHz, CDCl_3_) δ 7.61 (2H, d, *J* = 15.9 Hz), 7.00 (2H, d, *J* = 15.9 Hz), 6.79 (4H, d, *J* = 1.9 Hz), 6.60 (2H, t, *J* = 1.9 Hz), 4.14 (8H, t, *J* = 4.6 Hz), 3.76 (8H, t, *J* = 4.6 Hz), 3.46 (12H, s); ^13^C-NMR (100 MHz, CDCl_3_) δ 188.8, 160.2, 143.3, 136.6, 125.8, 107.2, 104.0, 70.9, 67.5, 59.2; LR-MS (EI) *m*/*z* 530 (M^+^, 100%); HR-MS (EI) Calcd. for C_29_H_38_O_9_: 530.2516, found: 530.2517.

*(1E,4E)-1,5-Bis(3,5-bis(2-hydroxyethoxy)phenyl)penta-1,4-dien-3-one* (GO-Y130). Yellow solid (EtOAc:hexane = 1:1) mp 171–173 °C. IR (solid): 3235, 1647, 1622, 1597, 1441 cm^−1^; ^1^H-NMR (600 MHz, DMSO-*d*_6_) δ 7.68 (2H, d, *J* = 15.7 Hz), 7.32 (2H, d, *J* = 15.7 Hz), 6.95 (4H, d, *J* = 1.9 Hz), 6.59–6.56 (2H, m), 4.86 (4H, t, *J* = 5.5 Hz), 4.03 (8H, t, *J* = 5.0 Hz), 3.72 (8H, dt, *J* = 5.5, 5.0 Hz); ^13^C-NMR (150 MHz, DMSO-*d*_6_) δ 188.5, 160.1, 142.8, 136.6, 126.1, 106.9, 103.6, 69.7, 59.5; LR-MS (FAB) *m*/*z* 475 ([M + H]^+^), 136.1 (100%), 137.1 (100%), 154.1 (100%); HR-MS (FAB) Calcd. for C_25_H_30_O_9_: 474.1887, found: 475.1990.

*(E)-3-(3,5-Bis(methoxymethoxy)phenyl)-1-(2-(3,5-bis(methoxymethoxy)phenyl)cyclopropyl)prop-2-en-1-one* (GO-Y131). Yellow oil; IR (CHCl_3_): 2955, 2826, 1674, 1647, 1592, 1453 cm^−1^; ^1^H-NMR (400 MHz, CDCl_3_) δ 7.51 (1H, d, *J* = 16.4 Hz), 6.91 (2H, d, *J* = 2.3 Hz), 6.85 (1H, d, *J* = 16.4 Hz), 6.76 (1H, t, *J* = 2.3 Hz), 6.61 (1H, t, *J* = 2.0 Hz), 6.49 (2H, d, *J* = 2.0 Hz), 5.17 (4H, s), 5.15 (4H, s), 3.50 (12H, s), 2.60–2.55 (1H, m), 2.50–2.45 (1H, m), 1.80–1.76 (1H, m), 1.48–1.43 (1H, m); ^13^C-NMR (100 MHz, CDCl_3_) δ 197.8, 158.6, 158.5, 143.2, 142.2, 136.7, 127.2, 109.6, 107.7, 107.2, 103.0, 94.6, 56.12, 56.09, 31.7, 29.8, 19.3; LR-MS (EI) *m*/*z* 488.1 (M^+^, 100%); HR-MS (EI) Calcd. for C_26_H_32_O_9_: 488.2046, found: 488.2047.

*Bis(2-(3,5-bis(methoxymethoxy)phenyl)cyclopropyl)methanone* (GO-Y132) (*syn*:*anti* = 1:1). Colorless oil; IR (CHCl_3_): 2955, 2903, 2826, 2360, 1680, 1594, 1463, 1439 cm^−1^; ^1^H-NMR (400 MHz, CDCl_3_) δ 6.60 (1H, t, *J* = 2.3 Hz), 6.58 (1H, t, *J* = 2.3 Hz), 6.45 (4H, d, *J* = 2.3 Hz), 5.14 (4H, s), 5.12 (4H, s), 3.47 (6H, s), 3.46 (6H, s), 2.56–2.47 (2H, m), 2.38–2.33 (2H, m), 1.73–1.65 (2H, m), 1.41–1.35 (2H, m); ^13^C-NMR (100 MHz, CDCl_3_) δ 206.7 (206.6), 158.41 (158.38), 143.03 (142.95), 107.52 (107.50), 102.91 (102.85), 94.5 (94.4), 56.03 (56.00), 33.2 (33.1), 29.4 (29.1), 19.5 (19.3); LR-MS (EI) *m*/*z* 502.1 (M^+^), 278.1 (100%); HR-MS (EI) Calcd. for C_27_H_34_O_9_: 502.2203, found: 502.2214.

*1,5-Bis(3,5-bis(methoxymethoxy)phenyl)pentan-3-one* (GO-Y133). Colorless solid; mp 31–32 °C. IR (CHCl_3_): 1714, 1595, 1460, 1440 cm^−1^; ^1^H-NMR (400 MHz, CDCl_3_) δ 6.58 (2H, t, *J* = 2.3 Hz), 6.51 (4H, d, *J* = 2.3 Hz), 5.13 (8H, s), 3.46 (12H, s), 2.84 (4H, t, *J* = 7.5 Hz), 2.70 (4H, t, *J* = 7.5 Hz); ^13^C-NMR (100 MHz, CDCl_3_) δ 208.6, 158.3, 143.5, 109.6, 102.6, 94.4, 56.0, 44.1, 29.8; LR-MS (EI) *m*/*z* 478(M^+^), 370 (100%); HR-MS (EI) Calcd. for C_25_H_34_O_9_: 478.2203, found: 478.2217.

*(E)-1,5-Bis(3,5-bis(methoxymethoxy)phenyl)pent-1-en-3-one* (GO-Y134). Colorless oil; IR (CHCl_3_): 1691, 1662, 1589, 1457 cm^−1^; ^1^H-NMR (400 MHz, CDCl_3_) δ 7.45 (1H, d, *J* = 16.0 Hz), 6.88 (2H, d, *J* = 2.4 Hz), 6.77 (1H, t, *J* = 2.4 Hz), 6.69 (1H, d, *J* = 16.0 Hz), 6.59 (1H, t, *J* = 2.0 Hz), 6.58 (2H, d, *J* = 2.0 Hz), 5.17 (4H, s), 5.14 (4H, s), 3.48 (6H, s), 3.47 (6H, s), 3.00–2.91 (4H, m); ^13^C-NMR (100 MHz, CDCl_3_) δ 199.1, 158.6, 158.4, 143.7, 142.4, 136.6, 126.8, 109.8, 109.5, 107.1, 102.7, 94.5, 56.12, 56.06, 42.1, 30.3; LR-MS (EI) *m*/*z* 476 (M^+^), (100%); HR-MS (EI) Calcd. for C_25_H_32_O_9_: 476.2036, found: 476.2046.

*(1E,4E)-1,5-Bis(3,5-bis((2-methoxyethoxy)methoxy)phenyl)penta-1,4-dien-3-one* (GO-Y147). Colorless oil; IR (CHCl_3_): 2923, 1654, 1598, 1449 cm^−1^; ^1^H-NMR (400 MHz, CDCl_3_) δ 7.64 (2H, d, *J* = 16.0 Hz), 7.04 (2H, d, *J* = 16.0 Hz), 6.99 (4H, d, *J* = 2.2 Hz), 6.81 (2H, t, *J* = 2.2 Hz), 5.30 (8H, s), 3.84 (8H, t, *J* = 4.6 Hz), 3.58 (8H, t, *J* = 4.6 Hz), 3.39 (12H, s); ^13^C-NMR (100 MHz, CDCl_3_) δ 188.7, 158.5, 143.0, 136.8, 126.0, 109.7, 107.2, 93.5, 71.5, 67.8, 67.7, 59.01, 58.95; LR-MS (EI) *m*/*z* 650 (M^+^), 89 (100%); HR-MS (EI) Calcd. for C_33_H_46_O_13_: 650.2938, found: 650.2933.

*Methyl (E)-3-(3,5-bis(methoxymethoxy)phenyl)acrylate* (GO-Y148). Colorless oil; IR (CHCl_3_): 2952, 1719, 1640, 1591, 1438, 1277 cm^−1^; ^1^H-NMR (400 MHz, CDCl_3_) δ 7.60 (1H, d, *J* = 16.2 Hz), 6.87 (2H, d, *J* = 2.3 Hz), 6.77 (1H, t, *J* = 2.3 Hz), 6.41 (1H, d, *J* = 16.2 Hz), 5.16 (4H, s), 3.80 (3H, s), 3.48 (6H, s); ^13^C-NMR (100 MHz, CDCl_3_) δ 167.2, 158.6, 144.5, 136.5, 118.6, 109.4, 106.9, 94.5, 56.1, 51.7; LR-MS (EI) *m*/*z* 282 (M^+^), 45 (100%); HR-MS (EI) Calcd. for C_14_H_18_O_6_: 282.1103, found: 282.1098.

*(E)-3-(3,5-Bis(methoxymethoxy)phenyl)acrylaldehyde* (GO-Y149). Colorless oil; IR (CHCl_3_): 1678, 1590, 1451, 1295 cm^−1^; ^1^H-NMR (400 MHz, CDCl_3_) δ 9.69 (1H, d, *J* = 7.7 Hz), 7.40 (1H, d, *J* = 15.9 Hz), 6.92 (2H, d, *J* = 2.1 Hz), 6.82 (1H, t, *J* = 2.1 Hz), 6.69 (1H, dd, *J* = 15.9, 7.7 Hz), 5.18 (4H, s), 3.49 (6H, s); ^13^C-NMR (100 MHz, CDCl_3_) δ 193.6, 158.7, 152.3, 136.0, 129.2, 109.7, 107.8, 94.5, 56.1; LR-MS (EI) *m*/*z* 252 (M^+^), 45 (100%); HR-MS (EI) Calcd. for C_13_H_16_O_5_: 252.0998, found: 252.0989.

*S-Propyl (E)-3-(3,5-bis(methoxymethoxy)phenyl)prop-2-enethioate* (GO-Y150). Colorless oil; IR (CHCl_3_): 2961, 2931, 1664, 1616, 1590, 1450, 1399, 1271 cm^−1^; ^1^H-NMR (400 MHz, CDCl_3_) δ 7.51 (1H, d, *J* = 15.7 Hz), 6.88 (2H, d, *J* = 2.3 Hz), 6.77 (1H, t, *J* = 2.3 Hz), 6.67 (1H, d, *J* = 15.7 Hz), 5.17 (4H, s), 3.48 (6H, s), 2.99 (2H, t, *J* = 7.3 Hz), 1.67 (2H, quin, *J* = 7.3 Hz), 1.01 (3H, t, *J* = 7.3 Hz); ^13^C-NMR (100 MHz, CDCl_3_) δ 189.7, 158.50, 158.48, 139.8, 136.2, 125.7, 109.4, 107.0, 94.4, 56.0, 30.8, 22.9, 13.3; LR-MS (EI) *m*/*z* 326 (M^+^), 45 (100%); HR-MS (EI) Calcd. for C_16_H_22_O_5_S: 326.1185, found: 326.1194.

*(E)-3-(3,5-Bis(methoxymethoxy)phenyl)-N-methylacrylamide* (GO-Y151). Colorless solid; mp 98–100 °C; IR (CHCl_3_): 3235, 1658, 1606, 1590 cm^−1^; ^1^H-NMR (400 MHz, CDCl_3_) δ 7.53 (1H, d, *J* = 15.7 Hz), 6.85 (2H, d, *J* = 2.1 Hz), 6.73 (1H, t, *J* = 2.1 Hz), 6.34 (1H, d, *J* = 15.7 Hz), 5.59 (1H, brs), 5.16 (4H, s), 3.48 (6H, s), 2.94 (3H, d, *J* = 4.9 Hz); ^13^C-NMR (100 MHz, CDCl_3_) δ 166.4, 158.6, 140.6, 137.0, 121.3, 109.1, 106.3, 94.6, 56.1, 26.5; LR-MS (EI) *m*/*z* 281 (M^+^), 45 (100%); HR-MS (EI) Calcd. for C_14_H_19_NO_5_: 281.1263, found: 281.1291.

*2,6-Bis((E)-3,5-bis(methoxymethoxy)benzylidene)cyclohexan-1-one* (GO-Y152). Yellow oil; IR (CHCl_3_): 2952, 2826, 1668, 1590, 1436, 1400 cm^−1^; ^1^H-NMR (400 MHz, CDCl_3_) δ 7.69 (2H, s), 6.81 (4H, d, *J* = 2.1 Hz), 6.74 (2H, t, *J* = 2.1 Hz), 5.17 (8H, s), 3.48 (12H, s), 2.91 (4H, t, *J* = 5.9 Hz), 1.79 (2H, quint, *J* = 5.9 Hz); ^13^C-NMR (100 MHz, CDCl_3_) δ 190.2, 158.1, 137.9, 136.68, 136.62, 111.8, 105.3, 94.6, 56.1, 28.4, 22.9; LR-MS (EI) *m*/*z* 514 (M^+^), 469 (100%); HR-MS (EI) Calcd. for C_28_H_34_O_9_: 514.2203, found: 514.2218.

*(E)-N-(3,5-Bis(methoxymethoxy)benzyl)-3-(3,5-bis(methoxymethoxy)phenyl)acrylamide* (GO-Y154). Colorless solid; mp 89–90 °C; IR (CHCl_3_): 3282, 2955, 1659, 1597, 1546, 1455 cm^−1^; ^1^H-NMR (400 MHz, CDCl_3_) δ 7.56 (1H, d, *J* = 15.5 Hz), 6.84 (2H, d, *J* = 2.3 Hz), 6.73 (1H, t, *J* = 2.3 Hz), 6.65 (3H, s), 6.39 (1H, d, *J* = 15.5 Hz), 6.05 (1H, brs), 5.15 (4H, s), 5.14 (4H, s), 4.49 (2H, d, *J* = 5.3 Hz), 3.47 (6H, s), 3.46 (6H, s); ^13^C-NMR (100 MHz, CDCl_3_) δ 165.5, 158.51, 158.49, 141.0, 140.5, 136.8, 121.1, 109.1, 106.3, 103.8, 94.5, 94.4, 56.04, 56.03, 43.8; LR-MS (EI) *m*/*z* 478 ([M + H]^+^), 69 (100%); HR-MS (EI) Calcd. for C_24_H_32_NO_9_: 478.2077, found: 478.2077.

*(1E,4E)-1-(Benzo[d][1,3]dioxol-5-yl)-5-(3,5-bis(methoxymethoxy)phenyl)penta-1,4-dien-3-one* (GO-Y156). Yellow plates (EtOAc:hexane = 1.5:1) mp 104–108 °C; IR (CHCl_3_): 1650, 1590, 1502, 1489, 1447 cm^−1^; ^1^H-NMR (400 MHz, CDCl_3_) δ 7.65 (1H, d, *J* = 16.1 Hz), 7.62 (1H, d, *J* = 16.1 Hz), 7.13 (1H, d, *J* = 1.8 Hz), 7.10 (1H, dd, *J* = 8.0, 1.8 Hz), 7.00 (1H, d, *J* = 16.1 Hz), 6.96 (2H, d, *J* = 2.3 Hz), 6.92 (1H, d, *J* = 16.1 Hz), 6.84 (1H, d, *J* = 7.9 Hz), 6.79 (1H, t, *J* = 2.3 Hz), 6.02 (2H, s), 5.19 (4H, s), 3.50 (6H, s); ^13^C-NMR (100 MHz, CDCl_3_) δ 188.6, 158.5, 149.9, 148.4, 143.2, 142.6, 136.9, 129.2, 126.3, 125.1, 123.3, 109.5, 108.6, 107.0, 106.6, 101.6, 94.5, 56.1; LR-MS (EI) *m*/*z* 398 (M^+^, 100%); HR-MS (EI) Calcd. for C_22_H_22_O_7_: 398.1366, found: 398.1392.

*(E)-3-(3,5-Bis(methoxymethoxy)phenyl)acrylic acid* (GO-Y157). Colorless needles (EtOAc:hexane = 1.5:1) mp 126–128 °C; IR (CHCl_3_): 2946, 2904, 1688, 1634, 1598, 1279 cm^−1^; ^1^H-NMR (400 MHz, CDCl_3_) δ 7.70 (1H, d, *J* = 15.9 Hz), 6.90 (2H, d, *J* = 2.1 Hz), 6.79 (1H, t, *J* = 2.1 Hz), 6.43 (1H, d, *J* = 15.9 Hz), 5.18 (4H, s), 3.49 (6H, s); ^13^C-NMR (100 MHz, CDCl_3_) δ 172.0, 158.6, 146.7, 136.1, 118.1, 109.6, 107.3, 94.5, 56.1; LR-MS (EI) *m*/*z* 268 (M^+^), 45 (100%); HR-MS (EI) Calcd. for C_13_H_16_O_6_: 268.0947, found: 268.9824.

*(E)-1,3-Bis(3,5-bis(methoxymethoxy)phenyl)prop-2-en-1-one* (GO-Y158). Yellow solid (EtOAc:hexane = 1:2); mp 82–83 °C; IR (CHCl_3_): 2955, 1665, 1590, 1450, 1400 cm^−1^; ^1^H-NMR (400 MHz, CDCl_3_) δ 7.69 (1H, d, *J* = 15.7 Hz), 7.37 (1H, d, *J* = 15.7 Hz), 7.30 (2H, d, *J* = 1.9 Hz), 6.98-6.96 (3H, m), 6.81 (1H, t, *J* = 2.1 Hz), 5.21 (4H, s), 5.19 (4H, s), 3.503 (6H, s), 3.496 (6H, s); ^13^C-NMR (100 MHz, CDCl_3_) δ 189.9, 158.6, 158.4, 144.7, 140.2, 136.9, 122.8, 109.8, 109.7, 109.1, 107.0, 94.54, 94.51, 56.2, 56.1; LR-MS (EI) *m*/*z* 448 (M^+^), 45 (100%); HR-MS (EI) Calcd. for C_23_H_28_O_9_: 448.1733, found: 448.1736.

*(1E,4Z,6E)-1,7-Bis(3,5-bis(methoxymethoxy)phenyl)-5-hydroxyhepta-1,4,6-trien-3-one* (GO-Y159). Yellow needles (EtOAc:hexane = 1:2); mp 200–201 °C; IR (CHCl_3_): 1631, 1591, 1146 cm^−1^; ^1^H-NMR (400 MHz, CDCl_3_) δ 7.57 (2H, d, *J* = 15.7 Hz), 6.91 (4H, d, *J* = 2.2 Hz), 6.77 (2H, t, *J* = 2.2 Hz), 6.59 (2H, d, *J* = 15.7 Hz), 5.86 (1H, s), 5.19 (8H, s), 3.50 (12H, s); ^13^C-NMR (100 MHz, CDCl_3_) δ 183.2, 158.6, 140.3, 137.1, 124.8, 109.4, 106.7, 101.9, 94.5, 56.1; LR-MS (FAB) *m*/*z* 517 ([M + H]^+^), 154 (100%); HR-MS (FAB) Calcd. for C_27_H_33_O_10_: 517.2074, found: 517.2074.

*(E)-2-(3,5-Bis(methoxymethoxy)benzyl)-5-(3,5-bis(methoxymethoxy)phenyl)-3-oxopent-4-ene-nitrile* (GO-Y160). Colorless oil; IR (CHCl_3_): 2926, 2201, 1697, 1595 cm^−1^; ^1^H-NMR (400 MHz, CDCl_3_) δ 7.64 (1H, d, *J* = 15.9 Hz), 6.90 (2H, d, *J* = 1.8 Hz), 6.88 (1H, d, *J* = 15.9 Hz), 6.83 (1H, t, *J* = 1.8 Hz), 6.66 (1H, t, *J* = 1.9 Hz), 6.63 (2H, d, *J* = 1.9 Hz), 5.18 (4H, s), 5.14 (4H, s), 3.91 (1H, dd, *J* = 8.9, 5.8 Hz), 3.49 (6H, s), 3.46 (6H, s), 3.24 (1H, dd, *J* = 14.0, 5.8 Hz), 3.10 (1H, dd, *J* = 14.0, 8.9 Hz); ^13^C-NMR (100 MHz, CDCl_3_) δ 188.9, 158.63, 158.59, 146.4, 138.1, 135.6, 122.1, 117.0, 110.3, 110.0, 108.0, 104.0, 94.5, 56.1, 56.0, 44.7, 35.3; LR-MS (EI) *m*/*z* 501 (M^+^), 45 (100%); HR-MS (EI) Calcd. for C_26_H_31_NO_9_: 501.1999, found: 501.1975.

*(E)-1,5-Bis(3,5-bis(methoxymethoxy)phenyl)-4,4-difluoropent-1-en-3-one* (GO-Y161). Colorless oil; IR (CHCl_3_): 2956, 2904, 1704, 1609, 1455, 1440 cm^−1^; ^1^H-NMR (400 MHz, CDCl_3_) δ 7.72 (1H, d, *J* = 15.7 Hz), 6.95 (1H, d, *J* = 15.7 Hz), 6.89 (2H, d, *J* = 2.3 Hz), 6.81 (1H, t, *J* = 2.3 Hz), 6.67 (1H, t, *J* = 2.0 Hz), 6.63 (2H, d, *J* = 2.0 Hz), 5.17 (4H, s), 5.13 (4H, s), 3.48 (6H, s), 3.45 (6H, s), 3.32 (2H, t, ^3^*J*_HF_ = 17.1 Hz); ^13^C-NMR (100 MHz, CDCl_3_) δ 189.1 (t, ^2^*J*_CF_ = 30.3 Hz), 158.6, 158.2, 147.2, 135.9, 133.2 (t, ^3^*J*_CF_ = 4.1 Hz), 118.7, 117.4 (t, ^1^*J*_CF_ = 253.9 Hz), 112.2, 110.0, 108.0, 104.1, 94.47, 94.45, 56.1, 56.0, 39.7 (t, ^2^*J*_CF_ = 23.7 Hz); LR-MS (EI) *m*/*z* 512 (M^+^), 45 (100%); HR-MS (EI) Calcd. for C_25_H_30_O_9_F_2_: 512.1858, found: 512.1855.

*(E)-1,5-Bis(3,5-bis(methoxymethoxy)phenyl)-4,4-dichloropent-1-en-3-one* (GO-Y162). Colorless oil; IR (CHCl_3_): 2955, 1725, 1698, 1610, 1595, 1456, 1439, 1400 cm^−1^; ^1^H-NMR (400 MHz, CDCl_3_) δ 7.78 (1H, d, *J* = 15.5 Hz), 7.38 (1H, d, *J* = 15.5 Hz), 6.95 (2H, d, *J* = 1.9 Hz), 6.82 (1H, t, *J* = 1.9 Hz), 6.75 (2H, d, *J* = 1.9 Hz), 6.72 (1H, m), 5.19 (4H, s), 5.15 (4H, s), 3.64 (2H, s), 3.49 (6H, s), 3.47 (6H, s); ^13^C-NMR (100 MHz, CDCl_3_) δ 186.8, 158.6, 157.8, 146.8, 136.2, 135.9, 119.5, 113.3, 110.0, 107.5, 104.1, 94.6, 94.5, 87.8, 56.15, 56.05, 47.7; LR-MS (EI) *m*/*z* 544 (M^+^), 45 (100%); HR-MS (EI) Calcd. for C_25_H_30_O_9_Cl_2_: 544.1267, found: 544.1265.

*(E)-1,5-Bis(3,5-bis(methoxymethoxy)phenyl)-4,4-dibromopent-1-en-3-one* (GO-Y163). Colorless oil; IR (CHCl_3_): 2954, 1688, 1610, 1593, 1455 cm^−1^; ^1^H-NMR (400 MHz, CDCl_3_) δ 7.76 (1H, d, *J* = 15.5 Hz), 7.55 (1H, d, *J* = 15.5 Hz), 6.96 (2H, d, *J* = 2.3 Hz), 6.83 (1H, t, *J* = 2.3 Hz), 6.80 (2H, d, *J* = 2.3 Hz), 6.73 (1H, t, *J* = 2.3 Hz), 5.19 (4H, s), 5.16 (4H, s), 3.83 (2H, s), 3.49 (6H, s), 3.48 (6H, s); ^13^C-NMR (100 MHz, CDCl_3_) δ 186.7, 158.6, 157.7, 146.1, 137.6, 136.2, 120.7, 113.3, 110.0, 107.4, 104.1, 94.7, 94.5, 69.1, 56.2, 56.1, 49.2; LR-MS (EI) *m*/*z* 634 (M^+^), 45 (100%); HR-MS (EI) Calcd. for C_25_H_30_O_9_^79^Br^81^Br: 634.0237, found: 634.0220.

*(1E,4E)-1,5-Bis(2,5-bis(methoxymethoxy)phenyl)penta-1,4-dien-3-one* (GO-Y164). Yellow solid (EtOAc:hexane = 1:6) mp 64–66 °C; IR (CHCl_3_): 2954, 1688, 1610, 1593, 1455 cm^−1^; ^1^H-NMR (400 MHz, CDCl_3_) δ 8.07 (2H, d, *J* = 16.1 Hz), 7.33 (2H, d, *J* = 2.9 Hz), 7.11 (2H, d, *J* = 16.1 Hz), 7.11 (2H, s), 7.05 (2H, dd, *J* = 9.3, 2.9 Hz), 5.22 (4H, s), 5.16 (4H, s), 3.51 (6H, s), 3.50 (6H, s); ^13^C-NMR (100 MHz, CDCl_3_) δ 189.3, 152.0, 151.5, 137.7, 126.4, 125.5, 119.8, 116.4, 115.2, 95.2, 95.0, 56.2, 55.9; LR-MS (EI) *m*/*z* 474 (M^+^), 45 (100%); HR-MS (EI) Calcd. for C_25_H_30_O_9_: 474.1890, found: 474.1895.

*(E)-2-((E)-3,5-Bis(methoxymethoxy)benzylidene)-5-(3,5-bis(methoxymethoxy)phenyl)-3-oxopent-4-enenitrile* (GO-Y165). Yellow solid; mp: 107–108 °C; IR (CHCl_3_): 2923, 2216, 1676, 1590, 1438 cm^−1^; ^1^H-NMR (600 MHz, CDCl_3_) δ 8.23 (1H, s), 7.83 (1H, d, *J* = 15.4 Hz), 7.46 (1H, d, *J* = 15.4 Hz), 7.40 (2H, d, *J* = 2.1 Hz), 7.00 (2H, d, *J* = 2.4 Hz), 6.95 (1H, t, *J* = 2.1 Hz), 6.85 (1H, t, *J* = 2.4 Hz), 5.22 (4H, s), 5.20 (4H, s), 3.51 (6H, s), 3.50 (6H, s); ^13^C-NMR (150 MHz, CDCl_3_) δ 181.9, 158.6, 153.8, 146.9, 136.1, 133.6, 121.1, 117.2, 112.2, 110.8, 110.3 110.1, 107.8, 94.6, 94.5, 56.23, 56.18; LR-MS (EI) *m*/*z* 499 (M^+^), 45 (100%); HR-MS (EI) Calcd. for C_26_H_29_NO_9_: 499.1842, found: 499.1849.

*3,5-Bis((E)-3,5-bis(methoxymethoxy)benzylidene)piperidin-4-one acetate* (GO-Y166). Yellow oil; IR (CHCl_3_): 2954, 1590, 1438, 1400 cm^−1^; ^1^H-NMR (400 MHz, CDCl_3_) δ 7.83 (2H, brs), 7.74 (2H, s), 6.76 (2H, t, *J* = 1.9 Hz), 6.72 (4H, d, *J* = 1.9 Hz), 5.17 (8H, s), 4.17 (4H, s), 3.48 (12H, s), 1.96 (3H, s); ^13^C-NMR (100 MHz, CDCl_3_) δ 186.8, 176.0, 158.2, 136.8, 136.7, 133.9, 111.7, 105.8, 94.5, 56.0, 47.0, 21.4; LR-MS (EI) *m*/*z* 515 (M^+^-HOAc, 100%); HR-MS (EI) Calcd. for C_27_H_33_NO_9_: 515.2155, found: 515.2132.

*3,5-Bis((E)-3,5-bis(methoxymethoxy)benzylidene)-1-methylpiperidin-4-one* (GO-Y167). Yellow oil; IR (CHCl_3_): 2951, 1674, 1591, 1448 cm^−1^; ^1^H-NMR (400 MHz, CDCl_3_) δ 7.71 (2H, s), 6.76-6.75 (6H, m), 5.17 (8H, s), 3.75 (4H, s), 3.49 (12H, s), 2.45 (3H, s); ^13^C-NMR (150 MHz, CDCl_3_) δ 186.6, 158.1, 137.0, 135.9, 133.5, 111.6, 105.4, 94.5, 56.8, 55.9, 45.6; LR-MS (EI) *m*/*z* 529 (M^+^, 100%); HR-MS (EI) Calcd. for C_28_H_35_NO_9_: 529.2312, found: 529.2294.

*(1Z,4E)-1,5-Bis(3,5-bis(methoxymethoxy)phenyl)-2-chloropenta-1,4-dien-3-one* (GO-Y168). Yellow amorphous solid (EtOAc:hexane = 1:6) mp 85–89 °C; IR (CHCl_3_): 2955, 1664, 1590, 1466 cm^−1^; ^1^H-NMR (400 MHz, CDCl_3_) δ 7.77 (1H, s), 7.73 (1H, d, *J* = 15.6 Hz), 7.50 (1H, d, *J* = 15.6 Hz), 7.25 (2H, d, *J* = 2.0 Hz), 6.98 (2H, d, *J* = 2.0 Hz), 6.83–6.81 (2H, m), 5.20, (8H, s), 3.50 (12H, s); ^13^C-NMR (100 MHz, CDCl_3_) δ 185.1, 158.6, 158.2, 145.7, 136.6, 135.3, 134.9, 131.1, 121.0, 112.2, 109.9, 107.3, 107.0, 94.6, 94.5, 56.18, 56.16; LR-MS (FAB) *m*/*z* 508 (M^+^), 45 (100%); HR-MS (FAB) Calcd. for C_25_H_29_O_9_Cl: 508.1500, found: 508.1504.

*(1Z,4E)-1,5-Bis(3,5-bis(methoxymethoxy)phenyl)-2-bromopenta-1,4-dien-3-one* (GO-Y169). Yellow amorphous solid (EtOAc:hexane = 1:6) mp 79–82 °C; IR (CHCl_3_): 2954, 1662, 1589, 1448 cm^−1^; ^1^H-NMR (600 MHz, CDCl_3_) δ 7.99 (1H, s), 7.70 (1H, d, *J* = 15.7 Hz), 7.47 (1H, d, *J* = 15.7 Hz), 7.25 (2H, d, *J* = 2.1 Hz), 6.97 (2H, d, *J* = 2.1 Hz), 6.828 (1H, t, *J* = 2.1 Hz), 6.821 (1H, t, *J* = 2.1 Hz), 5.20 (8H, s), 3.50 (12H, s); ^13^C-NMR (150 MHz, CDCl_3_) δ 185.6, 158.6, 158.1, 145.6, 138.9, 136.6, 135.7, 123.8, 121.7, 111.7, 109,9, 107.2, 107.0, 94.6, 94.5, 56.2, 56.1; LR-MS (EI) *m*/*z* 552 (M^+^), 45 (100%); HR-MS (EI) Calcd. for C_25_H_29_O_9_Br: 552.0995, found: 552.0957.

*(1Z,4E)-1,5-Bis(3,5-bis(methoxymethoxy)phenyl)-2-fluoropenta-1,4-dien-3-one* (GO-Y170). Yellow amorphous solid; mp 80–83 °C; IR (CHCl_3_): 2956, 1680, 1590, 1440 cm^−1^; ^1^H-NMR (600 MHz, CDCl_3_) δ 7.78 (1H, d, *J* = 15.7 Hz), 7.33 (1H, dd, *J* = 15.7, 2.7 Hz), 7.08 (2H, d, *J* = 2.2 Hz), 6.98 (2H, d, *J* = 2.1 Hz), 6.89 (1H, d, ^3^*J*_HF_ = 36.2 Hz), 6.82 (1H, t, *J* = 2.2 Hz), 6.79 (1H, t, *J* = 2.1 Hz), 5.20 (4H, s), 5.19 (4H, s), 3.50 (12H, s); ^13^C-NMR (150 MHz, CDCl_3_) δ 183.1 (d, ^2^*J*_CF_ = 31.5 Hz), 158.6, 158.4, 155.0 (d, ^1^*J*_CF_ = 275.1 Hz), 145.5, 136.5, 133.1 (^3^*J*_CF_ = 3.0 Hz), 120.1, 115.9 (^2^*J*_CF_ = 4.3 Hz), 112.0 (d, ^4^*J*_CF_ = 8.6 Hz), 110.0, 107.6, 106.8, 94.6, 94.5, 56.18, 56.16; LR-MS (FAB) *m*/*z* 492 (M^+^), 45 (100%); HR-MS (FAB) Calcd. for C_25_H_29_O_9_F: 492.1796, found: 492.1800.

*(E)-1-(3,5-Bis(methoxymethoxy)phenyl)-4,4-dimethylpent-1-en-3-one* (GO-Y171). Colorless oil; IR (CHCl_3_): 2965, 1684, 1610, 1589, 1477, 1283 cm^−1^; ^1^H-NMR (400 MHz, CDCl_3_) δ 7.58 (1H, *J* = 15.6 Hz), 7.06 (1H, d, *J* = 15.6 Hz), 6.90 (2H, d, *J* = 2.0 Hz), 6.79-6.77 (1H, m), 5.18 (4H, s), 3.49 (6H, s), 1.22 (9H, s); ^13^C-NMR (100 MHz, CDCl_3_) δ 203.9, 158.4, 142.5, 136.9, 121.3, 109.5, 106.5, 94.4, 56.0, 43.1, 26.2; LR-MS (EI) *m*/*z* 308 (M^+^), 45 (100%); HR-MS (EI) Calcd. for C_17_H_24_O_5_: 308.1624, found: 308.1610.

*(1Z,4E)-5-(3,5-Bis(methoxymethoxy)phenyl)-2-bromo-1-(2-bromo-3,5-bis(methoxymethoxy)phenyl)penta-1,4-dien-3-one* (GO-Y172). Yellow amorphous solid; mp 49–51 °C; IR (CHCl_3_): 2956, 2827, 1666, 1588, 1436, 1398 cm^−1^; ^1^H-NMR (600 MHz, CDCl_3_) δ 8.11 (1H, s), 7.73 (1H, d, *J* = 15.7 Hz), 7.42 (1H, d, *J* = 15.7 Hz), 7.23 (1H, d, *J* = 2.7 Hz), 6.97 (2H, d, *J* = 2.0 Hz), 6.93 (1H, d, *J* = 2.7 Hz), 6.81 (1H, t, *J* = 2.0 Hz), 5.26 (2H, s), 5.19 (4H, s), 5.18 (2H, s), 3.54 (3H, s), 3.49 (9H, s); ^13^C-NMR (150 MHz, CDCl_3_) δ 185.5, 158.6, 156.8, 154.7, 145.9, 139.1, 136.5, 136.4, 126.5, 121.7, 111.4, 109.9, 107.4, 107.0, 106.0, 95.3, 94.8, 94.5, 56.5, 56.20, 56.17; LR- MS (FAB) *m*/*z* 630 (M^+^), 45 (100%), 153 (100%); HR-MS (FAB) Calcd. for C_25_H_28_O_9_Br_2_: 630.0100, found: 630.0110.

### 3.3. Cell Growth Suppression Analysis

HCT116 was obtained from the Cell Resource Center for Biomedical Research (Institute of Development, Aging and Cancer, Tohoku University, Sendai, Japan). The growth suppressive effects of the compounds were measured for 48 h. Cell viability was assayed by quantifying the uptake and digestion of 2-(2-methoxy-4-nitrophenyl nitrophenyl)-3-(4-nitrophenyl)-5-(2,4-disulfophenyl)-2*H*-tetrazolium monosodium salt in accordance with the manufacturer’s instructions (Dojindo Laboratories, Kumamoto, Japan) using a 96-well plate reader, SpectraMax M2e (Molecular Devices, Sunnyvale, CA, USA). The percentage cell growth of the control, which was treated with 1% DMSO alone, was calculated and plotted, and then mean growth inhibitory concentration (GI_50_) was determined.

## 4. Conclusions

The following conclusions may be drawn from the SAR of GO-Y030: (1) 3,5-bismethoxymethoxy substituents do not play substantial roles as formaldehyde sources for antitumor activity; (2) the cross-conjugated dienone moiety, which could function as a Michael acceptor, is important for antitumor activity. Although it was suggested that the dienone moiety also plays a role in fixing conformation, our results demonstrate that the role as a Michael acceptor contributes more to antitumor activity; (3) one 3,5-bis(methoxymethoxy)phenyl ring and a Michael acceptor are not sufficient for sub-micromolar antitumor activity; (4) introduction of additional EWGs at the α-position of the carbonyl group did not increase the antitumor activity of particular C_5_-curcuminoids having two 3,5-bis(methoxymethoxy)phenyl rings. On the basis of the SAR studies, we confirmed how each moiety in GO-Y030 contributes to the observed antitumor activity. This finding increases the knowledge about C_5_-curcuminoids and other α,β-unsaturated carbonyl compounds as antitumor drug candidates.

## Supplementary Materials

Supplementary materials can be accessed at: http://www.mdpi.com/1420-3049/20/08/15374/s1.

## References

[1] Minassi A., Sánchez-Duffhues G., Collado J.A., Muñoz E., Appendino G. (2013). Dissecting the pharmacophore of curcumin. Which structural element is critical for which action?. J. Nat. Prod..

[2] Anand P., Thomas S.G., Kunnumakkara A.B., Sundaram C., Harikumar K.B., Sung B., Tharakan S.T., Misra K., Priyadarsini I.K., Rajasekharan K.N. (2008). Biological activities of curcumin and its analogues (Congeners) made by man and Mother Nature. Biochem. Pharmacol..

[3] Youssef K.M., El-Sherbeny M.A., El-Shafie F.S., Farag H.A., Al-Deeb O.A., Awadalla S.A.A. (2004). Synthesis of curcumin analogues as potential antioxidant, cancer chemopreventive agents. Arch. Pharm. (Weinheim).

[4] Sardjiman S.S., Reksohadiprodjo M.S., Hakim L., van der Goot H., Timmerman H. (1997). 1,5-Diphenyl-1,4-pentadiene-3-ones and cyclic analogues as antioxidative agents. Synthesis and structure-activity relationship. Eur. J. Med. Chem..

[5] Liang G., Li X., Chen L., Yang S., Wu X., Studer E., Gurley E., Hylemon P.B., Ye F., Li Y. (2008). Synthesis and anti-inflammatory activities of mono-carbonyl analogues of curcumin. Bioorg. Med. Chem. Lett..

[6] Adams B.K., Ferstl E.M., Davis M.C., Herold M., Kurtkaya S., Camalier R.F., Hollingshead M.G., Kaur G., Sausville E.A., Rickles F.R. (2004). Synthesis and biological evaluation of novel curcumin analogs as anti-cancer and anti-angiogenesis agents. Bioorg. Med. Chem..

[7] Adams B.K., Cai J., Armstrong J., Herold M., Lu Y.J., Sun A., Snyder J.P., Liotta D.C., Jones D.P., Shoji M. (2005). EF24, a novel synthetic curcumin analog, induces apoptosis in cancer cells via a redox-dependent mechanism. Anticancer Drugs.

[8] Tan K.L., Ali A., Du Y., Fu H., Jin H.X., Chin T.M., Khan M., Go M.L. (2014). Synthesis and evaluation of bisbenzylidenedioxotetrahydrothiopranones as activators of endoplasmic reticulum (ER) stress signaling pathways and apoptotic cell death in acute promyelocytic leukemic cells. J. Med. Chem..

[9] Liang G., Shao L., Wang Y., Zhao C., Chu Y., Xiao J., Zhao Y., Li X., Yang S. (2009). Exploration and synthesis of curcumin analogues with improved structural stability both *in vitro* and *in vivo* as cytotoxic agents. Bioorg. Med. Chem..

[10] Quincoces Suarez J.A., Rando D.G., Santos R.P., Gonçalves C.P., Ferreira E., de Carvalho J.E., Kohn L., Maria D.A., Faião-Flores F., Michalik D. (2010). New antitumoral agents I: *In vitro* anticancer activity and *in vivo* acute toxicity of synthetic 1,5-bis(4-hydroxy-3-methoxyphenyl)-1,4-pentadien-3-one and derivatives. Bioorg. Med. Chem..

[11] Ohori H., Yamakoshi H., Tomizawa M., Shibuya M., Kakudo Y., Takahashi A., Takahashi S., Kato S., Suzuki T., Ishioka C. (2006). Synthesis and biological analysis of new curcumin analogues bearing an enhanced potential for the medicinal treatment of cancer. Mol. Cancer Ther..

[12] Yamakoshi H., Ohori H., Kudo C., Sato A., Kanoh N., Ishioka C., Shibata H., Iwabuchi Y. (2010). Structure-activity relationship of C_5_-curcuminoids and synthesis of their molecular probes thereof. Bioorg. Med. Chem..

[13] Masuda T., Jitoe A., Isobe J., Nakatani N., Yonemori S. (1993). Anti-oxidative and anti-inflammatory curcumin-related phenolics from rhizomes of *Curcuma domestica*. Phytochemistry.

[14] Jiang J.L., Jin X.L., Zhang H., Su X., Qiao B., Yuan Y.J. (2012). Identification of antitumor constituents in curcuminoids from *Curcuma longa* L. based on the composition-activity relationship. J. Pharm. Biomed. Anal..

[15] Shibata H., Yamakoshi H., Sato A., Ohori H., Kakudo Y., Kudo C., Takahashi Y., Watanabe M., Takano H., Ishioka C. (2009). Newly synthesized curcumin analog has improved potential to prevent colorectal carcinogenesis *in vivo*. Cancer Sci..

[16] Kudo C., Yamakoshi H., Sato A., Ohori H., Ishioka C., Iwabuchi Y., Shibata H. (2011). Novel curcumin analogs, GO-Y030 and GO-Y078, are multi-targeted agents with enhanced abilities for multiple myeloma. Anticancer Res..

[17] Lin L., Liu Y., Li H., Li P.-K., Fuchs J., Shibata H., Iwabuchi Y., Lin J. (2011). Targeting colon cancer stem cells using a new curcumin analogue, GO-Y030. Br. J. Cancer.

[18] Yamakoshi H., Kanoh N., Kudo C., Sato A., Ueda K., Muroi M., Kon S., Satake M., Ohori H., Ishioka C. (2010). KSRP/FUBP2 is a binding protein of GO-Y086, a cytotoxic curcumin analogue. ACS Med. Chem. Lett..

[19] Guengerich F.P., MacDonald J.S. (2007). Applying mechanisms of chemical toxicity to predict drug safety. Chem. Res. Toxicol..

[20] Park B.K., Boobis A., Clarke S., Goldring C.E.P., Jones D., Kenna J.G., Lambert C., Laverty H.G., Naisbitt D.J., Nelson S. (2011). Managing the challenge of chemically reactive metabolites in drug development. Nat. Rev. Drug Discov..

[21] Park B.K., Pirmohamed M., Kitteringham N.R. (1998). Role of drug disposition in drug hypersensitivity: A chemical, molecular, and clinical perspective. Chem. Res. Toxicol..

[22] Dahmke I.N., Boettcher S.P., Groh M., Mahlknecht U. (2014). Cooking enhances curcumin anti-cancerogenic activity through pyrolytic formation of “deketene curcumin”. Food Chem..

[23] Ciaccio J.A., Aman C.E. (2006). “Instant Methylide” modification of the corey-chaykovsky cyclopropanation reaction. Synth. Commun..

[24] Baskin J.M., Prescher J.A., Laughlin S.T., Agard N.J., Chang P.V., Miller I.A., Lo A., Codelli J.A., Bertozzi C.R. (2007). Copper-free click chemistry for dynamic *in vivo* imaging. Proc. Natl. Acad. Sci. USA.

[25] Karimi S., Grohmann K.G., Todaro L. (1995). Intramolecular ring-opening of cyclopropanones by enolate anions. J. Org. Chem..

[26] Zheng S., Laxmi Y.R.S., David E., Dinkova-Kostova A.T., Shiavoni K.H., Ren Y., Zheng Y., Trevino I., Bumeister R., Ojima I. (2012). Synthesis, chemical reactivity as Michael acceptors, and biological potency of monocyclic cyanoenones, novel and highly potent anti-inflammatory and cytoprotective agents. J. Med. Chem..

[27] Parrish J.P., Kastrinsky D.B., Wolkenberg S.E., Igarashi Y., Boger D.L. (2003). DNA alkylation properties of yatakemycin. J. Am. Chem. Soc..

[28] MacMillan K.S., Boger D.L. (2009). Fundamental relationships between structure, reactivity, and biological activity for the duocarmycins and CC-1065. J. Med. Chem..

[29] Serafimova I.M., Pufall M.A., Krishnan S., Duda K., Cohen M.S., Maglathlin R.L., McFarland J.M., Miller R.M., Frödin M., Taunton J. (2012). Reversible targeting of noncatalytic cysteines with chemically tuned electrophiles. Nat. Chem. Biol..

[30] Wissner A., Mansour T.S. (2008). The development of HKI-272 and related compounds for the treatment of cancer. Arch. Pharm. (Weinheim).

[31] Honda T., Rounds B.V., Gribble G.W., Suh N., Wang Y., Sporn M.B. (1998). Design and synthesis of 2-cyano-3,12-dioxoolean-1,9-dien-28-oic acid, a novel and highly active inhibitor of nitric oxide production in mouse macrophages. Bioorg. Med. Chem. Lett..

[32] Al-Rifai N., Rücker H., Amslinger S. (2013). Opening or closing the lock? When reactivity is the key to biological activity. Chem. Eur. J..

[33] Lee K.H., Ab Aziz F.H., Syahida A., Abas F., Shaari K., Israf D.A., Lajis N.H. (2009). Synthesis and biological evaluation of curcumin-like diarylpentanoid analogues for anti-inflammatory, antioxidant and anti-tyrosinase activities. Eur. J. Med. Chem..

