# Supplementary material for: Structure-Activity Relationships of the Antitumor C5-Curcuminoid GO-Y030
Source: Molecules. 2015 Aug 24;20(8):15374–91. doi: 10.3390/molecules200815374 (PMC6332050; doi:10.3390/molecules200815374)
Supplement: Supplementary file 1 [file molecules-20-15374-s001.pdf]

Supplementary Materials

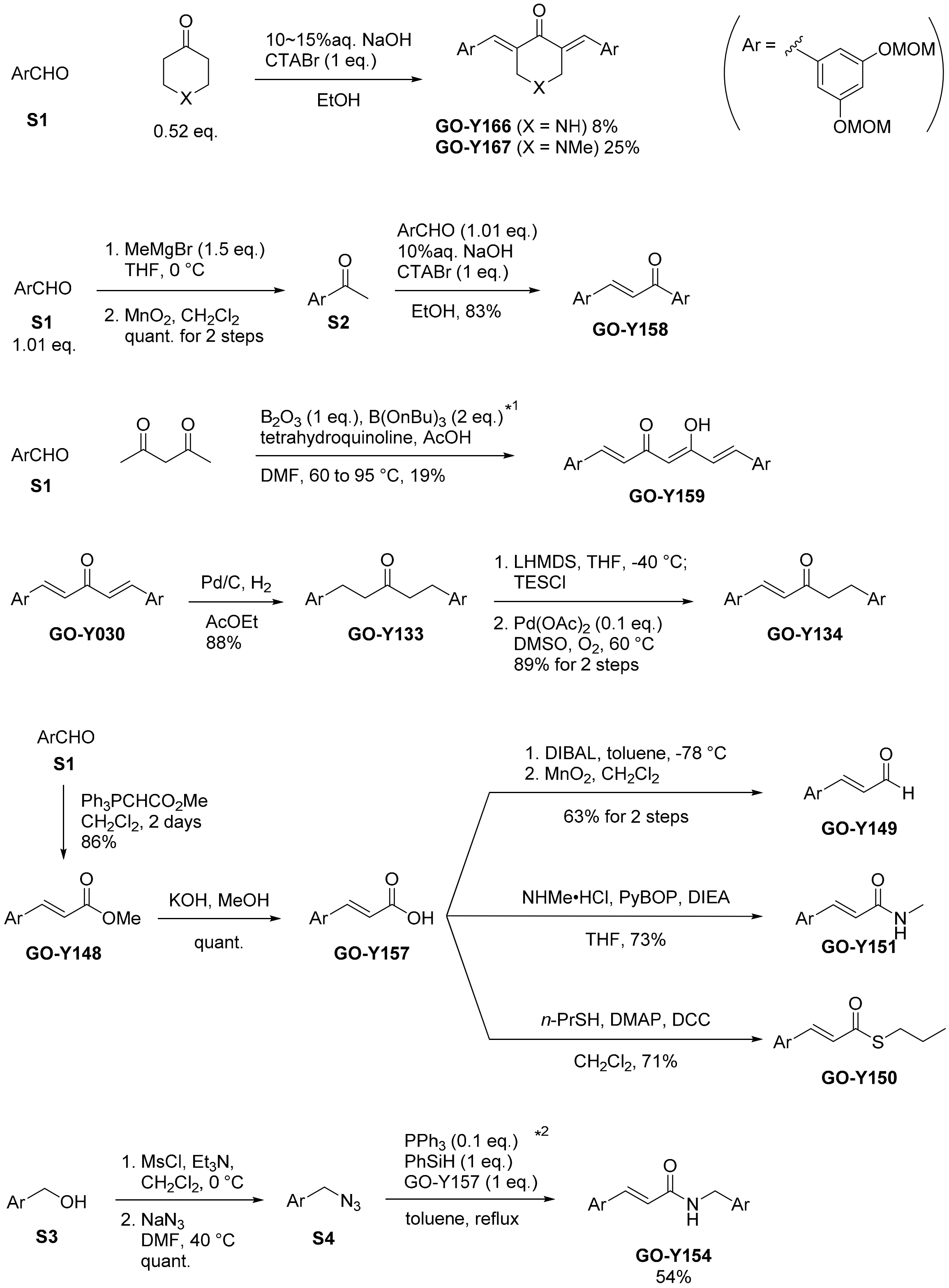

Figure S1. Supplementary Scheme. *1: Kim, M.K.; Jeong, W.; Kang, J.; Chong, Y. Significant enhancement in radical-scavenging activity of curcuminoids conferred by acetoxy substituent at the central methylene carbon. *Bioorg. Med. Chem.* **2011**, *19*, 3793–3800. *2: Kosal, A.D.; Wilson, E.E.; Ashfeld, B.L. Phosphine-Based Redox Catalysis in the Direct Traceless Staudinger Ligation of Carboxylic Acids and Azides. *Angew. Chem. Int. Ed.* **2012**, *51*, 12036–12040.

Compound Data (GO-Yxxx)

GO-Y129

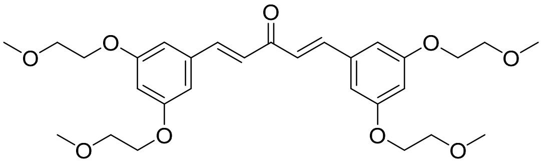

Yellow solid; mp 85–87 °C. IR ($CHCl_3$): 2927, 1652, 1590, 1441 cm^{-1}; ^{1}H-NMR (400 MHz, $CDCl_3$) δ 7.61 (2H, d, *J* = 15.9 Hz), 7.00 (2H, d, *J* = 15.9 Hz), 6.79 (4H, d, *J* = 1.9 Hz), 6.60 (2H, t, *J* = 1.9 Hz), 4.14 (8H, t, *J* = 4.6 Hz), 3.76 (8H, t, *J* = 4.6 Hz), 3.46 (12H, s); ^{13}C-NMR (100 MHz, $CDCl_3$) δ 188.8, 160.2, 143.3, 136.6, 125.8, 107.2, 104.0, 70.9, 67.5, 59.2; LR-MS (EI) *m*/*z* 530 (M^+, 100%); HR-MS (EI) Calcd. for $C_{29}H_{38}O_9$: 530.2516, found: 530.2517.

GO-Y130

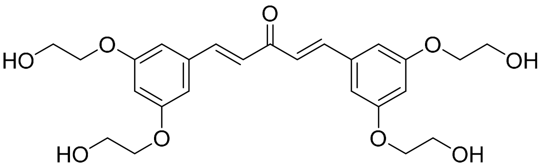

Yellow solid (AcOEt:hexane = 1:1) mp 171–173 °C. IR (solid): 3235, 1647, 1622, 1597, 1441 cm^{-1}; ^{1}H-NMR (600 MHz, DMSO-*d*$_6$) δ 7.68 (2H, d, *J* = 15.7 Hz), 7.32 (2H, d, *J* = 15.7 Hz), 6.95 (4H, d, *J* = 1.9 Hz), 6.59–6.56 (2H, m), 4.86 (4H, t, *J* = 5.5 Hz), 4.03 (8H, t, *J* = 5.0 Hz), 3.72 (8H, dt, *J* = 5.5, 5.0 Hz); ^{13}C-NMR (150 MHz, DMSO-*d*$_6$) δ 188.5, 160.1, 142.8, 136.6, 126.1, 106.9, 103.6, 69.7, 59.5; LR-MS (FAB) *m*/*z* 475 ($[M + H]^+$), 136.1 (100%), 137.1 (100%), 154.1 (100%); HR-MS (FAB) Calcd. for $C_{25}H_{30}O_9$: 474.1887, found: 475.1990.

GO-Y131

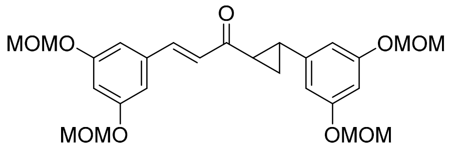

Yellow oil; IR ($CHCl_3$): 2955, 2826, 1674, 1647, 1592, 1453 cm^{-1}; ^{1}H-NMR (400 MHz, $CDCl_3$) δ 7.51 (1H, d, *J* = 16.4 Hz), 6.91 (2H, d, *J* = 2.3 Hz), 6.85 (1H, d, *J* = 16.4 Hz), 6.76 (1H, t, *J* = 2.3 Hz), 6.61 (1H, t, *J* = 2.0 Hz), 6.49 (2H, d, *J* = 2.0 Hz), 5.17 (4H, s), 5.15 (4H, s), 3.50 (12H, s), 2.60–2.55 (1H, m), 2.50–2.45 (1H, m), 1.80–1.76 (1H, m), 1.48–1.43 (1H, m); ^{13}C-NMR (100 MHz, $CDCl_3$) δ 197.8, 158.6, 158.5, 143.2, 142.2, 136.7, 127.2, 109.6, 107.7, 107.2, 103.0, 94.6, 56.12, 56.09, 31.7, 29.8, 19.3; LR-MS (EI) *m*/*z* 488.1 (M^+, 100%); HR-MS (EI) Calcd. for $C_{26}H_{32}O_9$: 488.2046, found: 488.2047.

GO-Y132 (Diastereo Mixture)

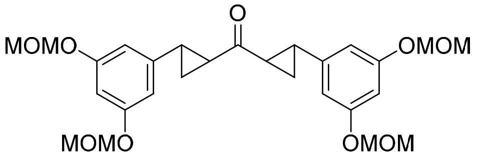

To a solution of $Me_3S(O)I$ (185 mg, 0.842 mmol) in DMSO (1.05 mL) was added NaH (20 mg, 0.84 mmol) washed with hexane. After stirring for 1 h at room temperature, to the reaction mixture was added GO-Y030 (100 mg, 0.842 mmol). After stirring for 16 min at 50–60 °C, the mixture was quenched with H_2O. The organic layer was separated and the aqueous layer was extracted with Et_2O (20 mL × 3). The combined organic phases were washed with brine (5 mL), dried over $MgSO_4$, and evaporated *in vacuo*. The residue was purified direct by silica gel chromatography (Hexanes/EtOAc = 2/1) to give GO-Y132 (66.2 mg, 0.132 mmol, 63%) as a colourless oil.

Colorless oil; IR ($CHCl_3$): 2955, 2903, 2826, 2360, 1680, 1594, 1463, 1439 cm^{-1}; ^{1}H-NMR (400 MHz, $CDCl_3$) δ 6.60 (1H, t, J = 2.3 Hz), 6.58 (1H, t, J = 2.3 Hz), 6.45 (4H, d, J = 2.3 Hz), 5.14 (4H, s), 5.12 (4H, s), 3.47 (6H, s), 3.46 (6H, s), 2.56–2.47 (2H, m), 2.38–2.33 (2H, m), 1.73–1.65 (2H, m), 1.41–1.35 (2H, m); ^{13}C-NMR (100 MHz, $CDCl_3$) δ 206.7 (206.6), 158.41 (158.38), 143.03 (142.95), 107.52 (107.50), 102.91 (102.85), 94.5 (94.4), 56.03 (56.00), 33.2 (33.1), 29.4 (29.1), 19.5 (19.3); LR-MS (EI) *m*/*z* 502.1 (M^+), 278.1 (100%); HR-MS (EI) Calcd. for $C_{27}H_{34}O_9$: 502.2203, found: 502.2214.

GO-Y133

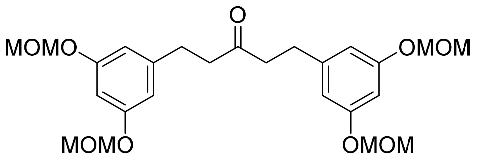

A mixture of GO-Y030 (780 mg, 1.64 mmol) and Pd/C (1.8 mg, 0.016 mmol) in AcOEt (16 mL) was stirred under hydrogen atmosphere for 18 h at room temperature. Additional Pd/C (2.6 mg, 0.024 mmol) was added to the reaction mixture. After 12 h, the reaction was sparged with nitrogen, filtered through Celite, and concentrated *in vacuo*. The residue was purified by flash column chromatography (2.5/1 to 2/1 Hexanes:EtOAc) to give GO-Y133 (694 mg, 1.45 mmol, 88%) as a white solid.

White solid; mp 31–32 °C. IR ($CHCl_3$): 1714, 1595, 1460, 1440 cm^{-1}; ^{1}H-NMR (400 MHz, $CDCl_3$) δ 6.58 (2H, t, J = 2.3 Hz), 6.51 (4H, d, J = 2.3 Hz), 5.13 (8H, s), 3.46 (12H, s), 2.84 (4H, t, J = 7.5 Hz), 2.70 (4H, t, J = 7.5 Hz); ^{13}C-NMR (100 MHz, $CDCl_3$) δ 208.6, 158.3, 143.5, 109.6, 102.6, 94.4, 56.0, 44.1, 29.8; LR-MS (EI) *m*/*z* 478(M^+), 370 (100%); HR-MS (EI) Calcd. for $C_{25}H_{34}O_9$: 478.2203, found: 478.2217.

GO-Y134

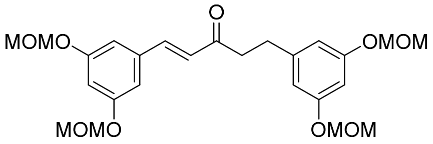

To a solution of GO-Y133 (426 mg, 0.890 mmol) in dry THF (17.8 mL) was added LHMDS (0.92 mL of 1.3 M solution in THF, 1.20 mmol) at −40 °C. After 15 min, to the reaction mixture was added TESCl (0.194 mL, 1.15 mmol) and the solution was warmed up to 0 °C over 15 min. After 45 min, the mixture was quenched with Et_3N (Pasteur pipette 16 drops) and saturated aqueous $NaHCO_3$. The resulting solution was extracted with AcOEt. The combined organic extracts were washed with brine, dried over $MgSO_4$ and concentrated *in vacuo*. The residue was purified by silica gel column chromatography (EtOAc/Hexane = 1/5 to 1/2) to give crude silyl enol ether (536 mg, 0.904 mmol). The crude was dissolved in DMSO (3 mL), to the solution was added $Pd(OAc)_2$ (39.9 mg, 0.178 mmol) and purged with oxygen. After being stirred for 9.5 h under oxygen atmosphere, the reaction mixture was quenched with $NaHCO_3$ (37.4 mg, 0.444 mmol) and filtered through Celite, and extracted with AcOEt. The combined organic extracts were washed with brine, dried over $MgSO_4$ and concentrated *in vacuo*. The residue was purified by column chromatography (EtOAc/Hexane = 1/3) to give GO-Y134 (382 mg, 0.0801 mmol, 89%).

Colorless oil; IR ($CHCl_3$): 1691, 1662, 1589, 1457 cm^{-1}; ^{1}H-NMR (400 MHz, $CDCl_3$) δ 7.45 (1H, d, J = 16.0 Hz), 6.88 (2H, d, J = 2.4 Hz), 6.77 (1H, t, J = 2.4 Hz), 6.69 (1H, d, J = 16.0 Hz), 6.59 (1H, t, J = 2.0 Hz), 6.58 (2H, d, J = 2.0 Hz), 5.17 (4H, s), 5.14 (4H, s), 3.48 (6H, s), 3.47 (6H, s), 3.00–2.91 (4H, m); ^{13}C-NMR (100 MHz, $CDCl_3$) δ 199.1, 158.6, 158.4, 143.7, 142.4, 136.6, 126.8, 109.8, 109.5, 107.1, 102.7, 94.5, 56.12, 56.06, 42.1, 30.3; LR-MS (EI) *m*/*z* 476 (M^+), (100%); HR-MS (EI) Calcd. for $C_{25}H_{32}O_9$: 476.2036, found: 478.2046.

GO-Y147

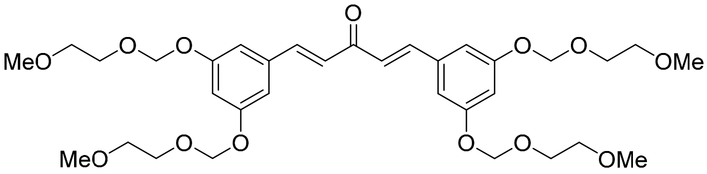

Colorless oil; IR ($CHCl_3$): 2923, 1654, 1598, 1449 cm^{-1}; ^{1}H-NMR (400 MHz, $CDCl_3$) δ 7.64 (2H, d, J = 16.0 Hz), 7.04 (2H, d, J = 16.0 Hz), 6.99 (4H, d, J = 2.2 Hz), 6.81 (2H, t, J = 2.2 Hz), 5.30 (8H, s), 3.84 (8H, t, J = 4.6 Hz), 3.58 (8H, t, J = 4.6 Hz), 3.39 (12H, s); ^{13}C-NMR (100 MHz, $CDCl_3$) δ 188.7, 158.5, 143.0, 136.8, 126.0, 109.7, 107.2, 93.5, 71.5, 67.8, 67.7, 59.01, 58.95; LR-MS (EI) *m*/*z* 650 (M^+), 89 (100%); HR-MS (EI) Calcd. for $C_{33}H_{46}O_{13}$: 650.2938, found: 650.2933.

GO-Y148

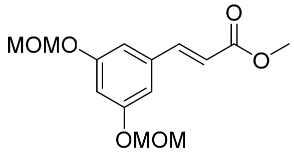

Colorless oil; IR ($CHCl_3$): 2952, 1719, 1640, 1591, 1438, 1277 cm^{-1}; ^{1}H-NMR (400 MHz, $CDCl_3$) δ 7.60 (1H, d, J = 16.2 Hz), 6.87 (2H, d, J = 2.3 Hz), 6.77 (1H, t, J = 2.3 Hz), 6.41 (1H, d, J = 16.2 Hz), 5.16 (4H, s), 3.80 (3H, s), 3.48 (6H, s); ^{13}C-NMR (100 MHz, $CDCl_3$) δ 167.2, 158.6, 144.5, 136.5, 118.6, 109.4, 106.9, 94.5, 56.1, 51.7; LR-MS (EI) *m/z* 282 (M$^+$), 45 (100%); HR-MS (EI) Calcd. for $C_{14}H_{18}O_6$: 282.1103, found: 282.1098.

GO-Y149

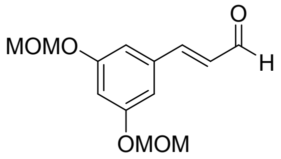

Colorless oil; IR ($CHCl_3$): 1678, 1590, 1451, 1295 cm^{-1}; ^{1}H-NMR (400 MHz, $CDCl_3$) δ 9.69 (1H, d, J = 7.7 Hz), 7.40 (1H, d, J = 15.9 Hz), 6.92 (2H, d, J = 2.1 Hz), 6.82 (1H, t, J = 2.1 Hz), 6.69 (1H, dd, J = 15.9, 7.7 Hz), 5.18 (4H, s), 3.49 (6H, s); ^{13}C-NMR (100 MHz, $CDCl_3$) δ 193.6, 158.7, 152.3, 136.0, 129.2, 109.7, 107.8, 94.5, 56.1; LR-MS (EI) *m/z* 252 (M$^+$), 45 (100%); HR-MS (EI) Calcd. for $C_{13}H_{16}O_5$: 252.0998, found: 252.0989.

GO-Y150

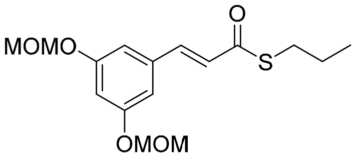

Colorless oil; IR ($CHCl_3$): 2961, 2931, 1664, 1616, 1590, 1450, 1399, 1271 cm^{-1}; ^{1}H-NMR (400 MHz, $CDCl_3$) δ 7.51 (1H, d, J = 15.7 Hz), 6.88 (2H, d, J = 2.3 Hz), 6.77 (1H, t, J = 2.3 Hz), 6.67 (1H, d, J = 15.7 Hz), 5.17 (4H, s), 3.48 (6H, s), 2.99 (2H, t, J = 7.3 Hz), 1.67 (2H, quin, J = 7.3 Hz), 1.01 (3H, t, J = 7.3 Hz); ^{13}C-NMR (100 MHz, $CDCl_3$) δ 189.7, 158.50, 158.48, 139.8, 136.2, 125.7, 109.4, 107.0, 94.4, 56.0, 30.8, 22.9, 13.3; LR-MS (EI) *m/z* 326 (M$^+$), 45 (100%); HR-MS (EI) Calcd. for $C_{16}H_{22}O_5S$: 326.1185, found: 326.1194.

GO-Y151

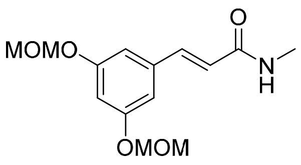

Colorless solid; mp 98–100 °C; IR ($CHCl_3$): 3235, 1658, 1606, 1590 cm^{-1}; ^{1}H-NMR (400 MHz, $CDCl_3$) δ 7.53 (1H, d, J = 15.7 Hz), 6.85 (2H, d, J = 2.1 Hz), 6.73 (1H, t, J = 2.1 Hz), 6.34 (1H, d, J = 15.7 Hz), 5.59 (1H, brs), 5.16 (4H, s), 3.48 (6H, s), 2.94 (3H, d, J = 4.9 Hz); ^{13}C-NMR (100 MHz, $CDCl_3$) δ 166.4, 158.6, 140.6, 137.0, 121.3, 109.1, 106.3, 94.6, 56.1, 26.5; LR-MS (EI) *m/z* 281 (M^+), 45 (100%); HR-MS (EI) Calcd. for $C_{14}H_{19}NO_5$: 281.1263, found: 281.1291.

GO-Y152

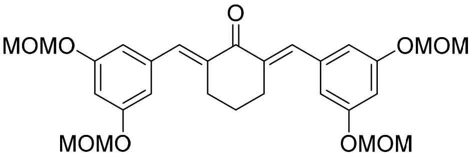

Yellow oil; IR ($CHCl_3$): 2952, 2826, 1668, 1590, 1436, 1400 cm^{-1}; ^{1}H-NMR (400 MHz, $CDCl_3$) δ 7.69 (2H, s), 6.81 (4H, d, J = 2.1 Hz), 6.74 (2H, t, J = 2.1 Hz), 5.17 (8H, s), 3.48 (12H, s), 2.91 (4H, t, J = 5.9 Hz), 1.79 (2H, quint, J = 5.9 Hz); ^{13}C-NMR (100 MHz, $CDCl_3$) δ 190.2, 158.1, 137.9, 136.68, 136.62, 111.8, 105.3, 94.6, 56.1, 28.4, 22.9; LR-MS (EI) *m/z* 514 (M^+), 469 (100%); HR-MS (EI) Calcd. for $C_{28}H_{34}O_9$: 514.2203, found: 514.2218.

GO-Y154

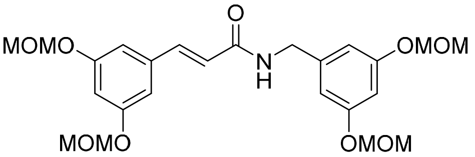

To a solution of GO-Y157 (36.6 mg, 0.136 mmol) and PPh_3 (3.7 mg, 0.0136 mmol) and PhSiH (16.7 μL, 0.136 mmol) in dry toluene (0.23 mL) was added azide **S4** (261 μL, 0.73 M toluene solution, 0.19 mmol). After refluxed for 1 day, the reaction mixture was purified by silica gel column chromatography (EtOAc/Hexane = 1/1.5 to EtOAc) to give GO-Y154 (35 mg, 0.073 mmol, 54%).

Colorless solid; mp 89–90 °C; IR ($CHCl_3$): 3282, 2955, 1659, 1597, 1546, 1455 cm^{-1}; ^{1}H-NMR (400 MHz, $CDCl_3$) δ 7.56 (1H, d, J = 15.5 Hz), 6.84 (2H, d, J = 2.3 Hz), 6.73 (1H, t, J = 2.3 Hz), 6.65 (3H, s), 6.39 (1H, d, J = 15.5 Hz), 6.05 (1H, brs), 5.15 (4H, s), 5.14 (4H, s), 4.49 (2H, d, J = 5.3 Hz), 3.47 (6H, s), 3.46 (6H, s); ^{13}C-NMR (100 MHz, $CDCl_3$) δ 165.5, 158.51, 158.49, 141.0, 140.5, 136.8, 121.1, 109.1, 106.3, 103.8, 94.5, 94.4, 56.04, 56.03, 43.8; LR-MS (EI) *m/z* 478 ($[M + H]^+$), 69 (100%); HR-MS (EI) Calcd. for $C_{24}H_{32}NO_9$: 478.2077, found: 478.2077.

GO-Y156

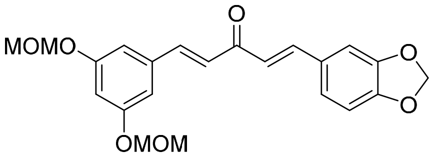

Yellow plate (AcOEt:hexane = 1.5:1) mp 104–108 °C; IR ($CHCl_3$): 1650, 1590, 1502, 1489, 1447 cm^{-1}; ^{1}H-NMR (400 MHz, $CDCl_3$) δ 7.65 (1H, d, *J* = 16.1 Hz), 7.62 (1H, d, *J* = 16.1 Hz), 7.13 (1H, d, *J* = 1.8 Hz), 7.10 (1H, dd, *J* = 8.0, 1.8 Hz), 7.00 (1H, d, *J* = 16.1 Hz), 6.96 (2H, d, *J* = 2.3 Hz), 6.92 (1H, d, *J* = 16.1 Hz), 6.84 (1H, d, *J* = 7.9 Hz), 6.79 (1H, t, *J* = 2.3 Hz), 6.02 (2H, s), 5.19 (4H, s), 3.50 (6H, s); ^{13}C-NMR (100 MHz, $CDCl_3$) δ 188.6, 158.5, 149.9, 148.4, 143.2, 142.6, 136.9, 129.2, 126.3, 125.1, 123.3, 109.5, 108.6, 107.0, 106.6, 101.6, 94.5, 56.1; LR-MS (EI) *m*/*z* 398 (M^+, 100%); HR-MS (EI) Calcd. for $C_{22}H_{22}O_7$: 398.1366, found: 398.1392.

GO-Y157

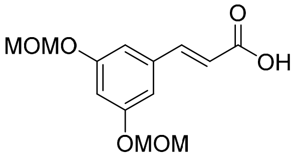

Colorless needle (AcOEt:hexane = 1.5:1) mp 126–128 °C; IR ($CHCl_3$): 2946, 2904, 1688, 1634, 1598, 1279 cm^{-1}; ^{1}H-NMR (400 MHz, $CDCl_3$) δ 7.70 (1H, d, *J* = 15.9 Hz), 6.90 (2H, d, *J* = 2.1 Hz), 6.79 (1H, t, *J* = 2.1 Hz), 6.43 (1H, d, *J* = 15.9 Hz), 5.18 (4H, s), 3.49 (6H, s); ^{13}C-NMR (100 MHz, $CDCl_3$) δ172.0, 158.6, 146.7, 136.1, 118.1, 109.6, 107.3, 94.5, 56.1; LR-MS (EI) *m*/*z* 268 (M^+), 45 (100%); HR-MS (EI) Calcd. for $C_{13}H_{16}O_6$: 268.0947, found: 268.9824.

GO-Y158

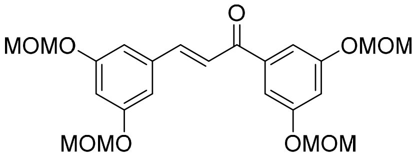

Yellow solid (AcOEt:hexane = 1:2) mp 82–83 °C; IR ($CHCl_3$): 2955, 1665, 1590, 1450, 1400 cm^{-1}; ^{1}H-NMR (400 MHz, $CDCl_3$) δ 7.69 (1H, d, *J* = 15.7 Hz), 7.37 (1H, d, *J* = 15.7 Hz), 7.30 (2H, d, *J* = 1.9 Hz), 6.98–6.96 (3H, m), 6.81 (1H, t, *J* = 2.1 Hz), 5.21 (4H, s), 5.19 (4H, s), 3.503 (6H, s), 3.496 (6H, s); ^{13}C-NMR (100 MHz, $CDCl_3$) δ 189.9, 158.6, 158.4, 144.7, 140.2, 136.9, 122.8, 109.8, 109.7, 109.1, 107.0, 94.54, 94.51, 56.2, 56.1; LR-MS (EI) *m*/*z* 448 (M^+), 45 (100%); HR-MS (EI) Calcd. for $C_{23}H_{28}O_9$: 448.1733, found: 448.1736.

GO-Y159

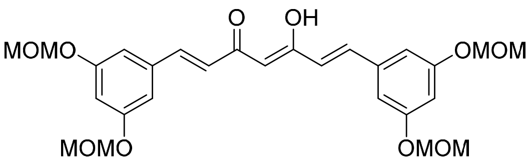

Yellow needle (AcOEt:hexane = 1:2) mp 200–201 °C; IR ($CHCl_3$): 1631, 1591, 1146 cm^{-1}; ^{1}H-NMR (400 MHz, $CDCl_3$) δ 7.57 (2H, d, *J* = 15.7 Hz), 6.91 (4H, d, *J* = 2.2 Hz), 6.77 (2H, t, *J* = 2.2 Hz), 6.59 (2H, d, *J* = 15.7 Hz), 5.86 (1H, s), 5.19 (8H, s), 3.50 (12H, s); ^{13}C-NMR (100 MHz,

$CDCl_3$) δ 183.2, 158.6, 140.3, 137.1, 124.8, 109.4, 106.7, 101.9, 94.5, 56.1; LR-MS (FAB) *m/z* 517 ($[M + H]^+$), 154 (100%); HR-MS (FAB) Calcd. for $C_{27}H_{33}O_{10}$: 517.2074, found: 517.2074.

GO-Y160

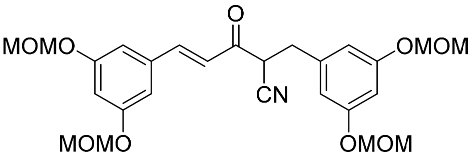

To a solution of GO-Y134 (82 mg, 0.172 mmol) in dry THF (5 ml) was added LHMDS (0.39 mL of 1.3 M solution in THF, 0.52 mmol) at −40 °C. After 35 min, to the reaction mixture was added TsCN (124 mg, 0.688 mmol) in dry THF (1 mL) at −40 °C. After 20 min, the mixture was quenched with saturated aqueous NH_4Cl. The resulting solution was extracted with AcOEt. The combined organic extracts were washed with brine, dried over $MgSO_4$ and concentrated *in vacuo*. The residue was purified by flash silica gel column chromatography (EtOAc/Hexane = 1/4 to 1/2) to give GO-Y160 (41.8 mg, 0.0832 mmol, 48%) as a colorless oil.

Colorless oil; IR ($CHCl_3$): 2926, 2201, 1697, 1595 cm^{-1}; ^{1}H-NMR (400 MHz, $CDCl_3$) δ 7.64 (1H, d, *J* = 15.9 Hz), 6.90 (2H, d, *J* = 1.8 Hz), 6.88 (1H, d, *J* = 15.9 Hz), 6.83 (1H, t, *J* = 1.8 Hz), 6.66 (1H, t, *J* = 1.9 Hz), 6.63 (2H, d, *J* = 1.9 Hz), 5.18 (4H, s), 5.14 (4H, s), 3.91 (1H, dd, *J* = 8.9, 5.8 Hz), 3.49 (6H, s), 3.46 (6H, s), 3.24 (1H, dd, *J* = 14.0, 5.8 Hz), 3.10 (1H, dd, *J* = 14.0, 8.9 Hz); ^{13}C-NMR (100 MHz, $CDCl_3$) δ 188.9, 158.63, 158.59, 146.4, 138.1, 135.6, 122.1, 117.0, 110.3, 110.0, 108.0, 104.0, 94.5, 56.1, 56.0, 44.7, 35.3; LR-MS (EI) *m*/*z* 501 (M^+), 45 (100%); HR-MS (EI) Calcd. for $C_{26}H_{31}NO_9$: 501.1999, found: 501.1975.

(((1*E*,3*Z*)-1,5-Bis(3,5-bis(methoxymethoxy)phenyl)penta-1,3-dien-3-yl)oxy)triethylsilane (**S5**)

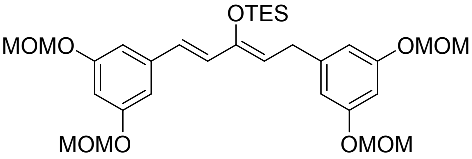

To a solution of GO-Y134 (188 mg, 0.393 mmol) in dry THF (16 ml) was added LHMDS (0.81 mL of 1.3 M solution in THF, 1.05 mmol) at −40 °C. After 7 min, to the reaction mixture was added TESCl (171 μL, 1.02 mmol) at −40 °C. After 1 h, the mixture was quenched with Et_3N (Pasteur pipette 14 drops) and saturated aqueous $NaHCO_3$. The resulting solution was extracted with AcOEt. The combined organic extracts were washed with brine, dried over $MgSO_4$ and concentrated *in vacuo*. The residue was purified by flash silica gel column chromatography (EtOAc/Hexane = 1/4 to 1/2) to give silyl enol ether **S5** (208 mg, 0.0350 mmol, 89%) that was used in the next reaction without further purification.

GO-Y161

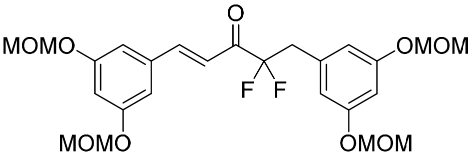

To a Selectfluor® (113 mg, 0.32 mmol) was added resultant crude silyl enol ether **S5** (47.4 mg, 0.802 mmol) in dry DMF (8 mL) at 0 °C. After being stirred for 29 h at room temperature, the reaction mixture was quenched with saturated aqueous $NaHCO_3$. The resulting solution was extracted with AcOEt. The combined organic extracts were washed with brine, dried over $MgSO_4$ and concentrated *in vacuo*. The residue was purified by flash silica gel column chromatography (EtOAc/Hexane = 1/4 to 1/2) to give α-fluoro enone **8** (26.6 mg, 0.0536 mmol, 67%) as a colorless oil. After same procedure was repeated, GO-Y161 (9.85 mg, 0.0192 mmol, 94% for 2 steps) was given.

Colorless oil; IR ($CHCl_3$): 2956, 2904, 1704, 1609, 1455, 1440 cm^{-1}; ^{1}H-NMR (400 MHz, $CDCl_3$) δ 7.72 (1H, d, J = 15.7 Hz), 6.95 (1H, d, J = 15.7 Hz), 6.89 (2H, d, J = 2.3 Hz), 6.81 (1H, t, J = 2.3 Hz), 6.67 (1H, t, J = 2.0 Hz), 6.63 (2H, d, J = 2.0 Hz), 5.17 (4H, s), 5.13 (4H, s), 3.48 (6H, s), 3.45 (6H, s), 3.32 (2H, t, $^3J_{HF}$ = 17.1 Hz); ^{13}C-NMR (100 MHz, $CDCl_3$) δ 189.1 (t, $^2J_{CF}$ = 30.3 Hz), 158.6, 158.2, 147.2, 135.9, 133.2 (t, $^3J_{CF}$ = 4.1 Hz), 118.7, 117.4 (t, $^1J_{CF}$ = 253.9 Hz), 112.2, 110.0, 108.0, 104.1, 94.47, 94.45, 56.1, 56.0, 39.7 (t, $^2J_{CF}$ = 23.7 Hz); LR-MS (EI) *m/z* 512 (M^+), 45 (100%); HR-MS (EI) Calcd. for $C_{25}H_{30}O_9F_2$: 512.1858, found: 512.1855.

GO-Y162

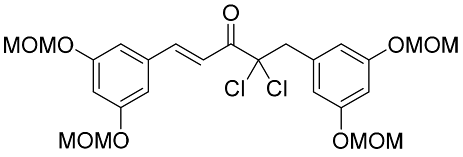

To a solution of GO-Y134 (45.0 mg, 0.0944 mmol) in dry THF (1 mL) was added LHMDS (0.10 mL of 1.3 M solution in THF, 0.141 mmol) at −40 °C. After 20 min, to the reaction mixture was added NCS (37.8 mg, 0.283 mmol) in dry THF (0.8 mL). After being stirred for 30 min at −40 °C, the reaction mixture was quenched with saturated aqueous $NaHCO_3$. The resulting solution was extracted with AcOEt. The combined organic extracts were washed with brine, dried over $MgSO_4$ and concentrated *in vacuo*. The residue was purified by flash silica gel column chromatography (EtOAc/Hexane = 1/5 to 1/2) to give α-chloro enone **11** (24.1 mg, 0.0471 mmol, 50%) and GO-Y162 (14.0 mg, 0.0257 mmol, 27%). α-Chloro enone **11** was converted to GO-Y162 (11.1 mg, 0.0204 mmol, 43%) with repeated procedure.

Colorless oil; IR ($CHCl_3$): 2955, 1725, 1698, 1610, 1595, 1456, 1439, 1400 cm^{-1}; ^{1}H-NMR (400 MHz, $CDCl_3$) δ 7.78 (1H, d, J = 15.5 Hz), 7.38 (1H, d, J = 15.5 Hz), 6.95 (2H, d, J = 1.9 Hz), 6.82 (1H, t, J = 1.9 Hz), 6.75 (2H, d, J = 1.9 Hz), 6.72 (1H, m), 5.19 (4H, s), 5.15 (4H, s), 3.64 (2H, s), 3.49 (6H, s), 3.47 (6H, s); ^{13}C-NMR (100 MHz, $CDCl_3$) δ 186.8, 158.6, 157.8, 146.8, 136.2, 135.9, 119.5,

113.3, 110.0, 107.5, 104.1, 94.6, 94.5, 87.8, 56.15, 56.05, 47.7; LR-MS (EI) *m/z* 544 (M^+), 45 (100%); HR-MS (EI) Calcd. for $C_{25}H_{30}O_9Cl_2$: 544.1267, found: 544.1265.

GO-Y163

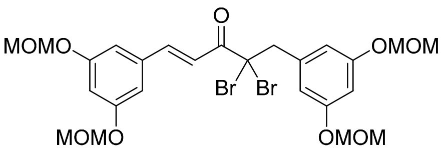

To a solution of GO-Y134 (25.5 mg, 0.0534 mmol) in dry THF (1 mL) was added LHMDS (0.08 mL of 1.3 M solution in THF, 0.11 mmol) at −40 °C. After 30 min, to the reaction mixture was added NBS (23.7 mg, 0.133 mmol) in dry THF (1.5 mL) at −40 °C. After 30 min, the mixture was quenched with saturated aqueous $NaHCO_3$. The resulting solution was extracted with AcOEt. The combined organic extracts were washed with brine, dried over $MgSO_4$ and concentrated *in vacuo*. The residue was purified by flash silica gel column chromatography (EtOAc/Hexane = 1/4) to give GO-Y163 (14.7 mg, 0.0232 mmol, 43%) as a colorless oil.

Colorless oil; IR ($CHCl_3$): 2954, 1688, 1610, 1593, 1455 cm^{-1}; ^{1}H-NMR (400 MHz, $CDCl_3$) δ 7.76 (1H, d, J = 15.5 Hz), 7.55 (1H, d, J = 15.5 Hz), 6.96 (2H, d, J = 2.3 Hz), 6.83 (1H, t, J = 2.3 Hz), 6.80 (2H, d, J = 2.3 Hz), 6.73 (1H, t, J = 2.3 Hz), 5.19 (4H, s), 5.16 (4H, s), 3.83 (2H, s), 3.49 (6H, s), 3.48 (6H, s); ^{13}C-NMR (100 MHz, $CDCl_3$) δ 186.7, 158.6, 157.7, 146.1, 137.6, 136.2, 120.7, 113.3, 110.0, 107.4, 104.1, 94.7, 94.5, 69.1, 56.2, 56.1, 49.2; LR-MS (EI) *m/z* 634 (M^+), 45 (100%); HR-MS (EI) Calcd. for $C_{25}H_{30}O_9{}^{79}Br^{81}Br$: 634.0237, found: 634.0220.

GO-Y164

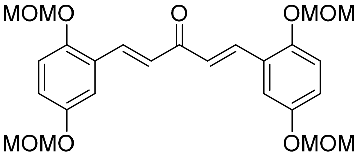

Yellow solid (AcOEt:hexane = 1:6) mp 64–66 °C; IR ($CHCl_3$): 2954, 1688, 1610, 1593, 1455 cm^{-1}; ^{1}H-NMR (400 MHz, $CDCl_3$) δ 8.07 (2H, d, J = 16.1 Hz), 7.33 (2H, d, J = 2.9 Hz), 7.11 (2H, d, J = 16.1 Hz), 7.11 (2H, s), 7.05 (2H, dd, J = 9.3, 2.9 Hz), 5.22 (4H, s), 5.16 (4H, s), 3.51 (6H, s), 3.50 (6H, s); ^{13}C-NMR (100 MHz, $CDCl_3$) δ 189.3, 152.0, 151.5, 137.7, 126.4, 125.5, 119.8, 116.4, 115.2, 95.2, 95.0, 56.2, 55.9; LR-MS (EI) *m/z* 474 (M^+), 45 (100%); HR-MS (EI) Calcd. for $C_{25}H_{30}O_9$: 474.1890, found: 474.1895.

GO-Y165

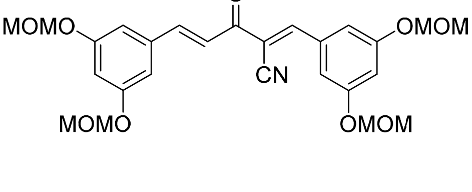

To a solution of GO-Y160 (4.25 mg, 0.00847 mmol) in dry benzene (0.8 mL) was added DDQ (2.47 mg, 0.0108 mmol). After 23 h, to the reaction mixture was added DDQ (4.8 mg, 15 mmol). After being stirred for 7 h at room temperature, the reaction was diluted with Et_2O, quenched with saturated aqueous $NaHCO_3$, and extracted with Et_2O. The combined organic layers were washed with saturated aqueous $NaHCO_3$, dried over $MgSO_4$, and concentrated *in vacuo*. The residue was purified by flash silica gel column chromatography (EtOAc/Hexane = 1/3 to 1/1) to give GO-Y165 (1.28 mg, 0.00272 mmol, 32%) and GO-Y160 (1.36 mg, 0.00271 mmol, 32%).

Yellow solid; mp: 107 – 108 °C; IR ($CHCl_3$): 2923, 2216, 1676, 1590, 1438 cm^{-1}; ^{1}H-NMR (600 MHz, $CDCl_3$) δ 8.23 (1H, s), 7.83 (1H, d, *J* = 15.4 Hz), 7.46 (1H, d, *J* = 15.4 Hz), 7.40 (2H, d, *J* = 2.1 Hz), 7.00 (2H, d, *J* = 2.4 Hz), 6.95 (1H, t, *J* = 2.1 Hz), 6.85 (1H, t, *J* = 2.4 Hz), 5.22 (4H, s), 5.20 (4H, s), 3.51 (6H, s), 3.50 (6H, s); ^{13}C-NMR (150 MHz, $CDCl_3$) δ 181.9, 158.6, 153.8, 146.9, 136.1, 133.6, 121.1, 117.2, 112.2, 110.8, 110.3 110.1, 107.8, 94.6, 94.5, 56.23, 56.18; LR-MS (EI) *m*/*z* 499 (M^+), 45 (100%); HR-MS (EI) Calcd. for $C_{26}H_{29}NO_9$: 499.1842, found: 499.1849.

GO-Y166

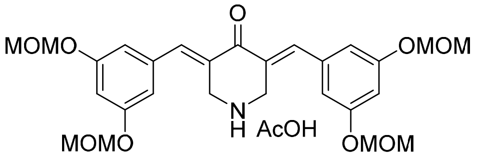

Yellow oil; IR ($CHCl_3$): 2954, 1590, 1438, 1400 cm^{-1}; ^{1}H-NMR (400 MHz, $CDCl_3$) δ 7.83 (2H, brs), 7.74 (2H, s), 6.76 (2H, t, *J* = 1.9 Hz), 6.72 (4H, d, *J* = 1.9 Hz), 5.17 (8H, s), 4.17 (4H, s), 3.48 (12H, s), 1.96 (3H, s); ^{13}C-NMR (100 MHz, $CDCl_3$) δ 186.8, 176.0, 158.2, 136.8, 136.7, 133.9, 111.7, 105.8, 94.5, 56.0, 47.0, 21.4; LR-MS (EI) *m*/*z* 515 (M^+-HOAc, 100%); HR-MS (EI) Calcd. for $C_{27}H_{33}NO_9$: 515.2155, found: 515.2132.

GO-Y167

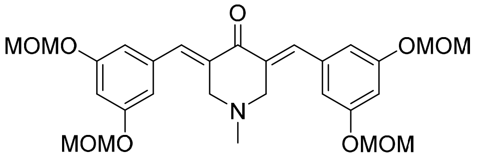

Yellow oil; IR ($CHCl_3$): 2951, 1674, 1591, 1448 cm^{-1}; ^{1}H-NMR (400 MHz, $CDCl_3$) δ 7.71 (2H, s), 6.76–6.75 (6H, m), 5.17 (8H, s), 3.75 (4H, s), 3.49 (12H, s), 2.45 (3H, s); ^{13}C-NMR (150 MHz, $CDCl_3$) δ 186.6, 158.1, 137.0, 135.9, 133.5, 111.6, 105.4, 94.5, 56.8, 55.9, 45.6; LR-MS (EI) *m*/*z* 529 (M^+, 100%); HR-MS (EI) Calcd. for $C_{28}H_{35}NO_9$: 529.2312, found: 529.2294.

GO-Y168

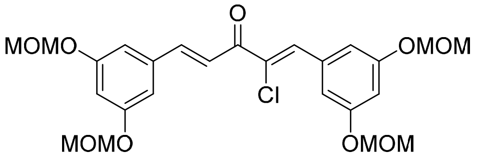

To a solution of GO-Y162 (10.1 mg, 0.0184 mmol) in dry DMF (1.8 mL) was added Li_2CO_3 (2.7 mg, 0.0369 mmol). After being stirred for 8 h at 120 °C, the reaction was quenched with saturated aqueous NH_4Cl, and extracted with Et_2O. The combined organic layers were dried over $MgSO_4$, and concentrated *in vacuo*. The residue was purified by flash silica gel column chromatography (EtOAc/Hexane = 1/4) to give GO-Y168 (6.76 mg, 0.0132 mmol, 72%) as a yellow amorphous.

Yellow amorphous (AcOEt:hexane = 1:6) mp 85–89 °C; IR ($CHCl_3$): 2955, 1664, 1590, 1466 cm^{-1}; ^{1}H-NMR (400 MHz, $CDCl_3$) δ 7.77 (1H, s), 7.73 (1H, d, J = 15.6 Hz), 7.50 (1H, d, J = 15.6 Hz), 7.25 (2H, d, J = 2.0 Hz), 6.98 (2H, d, J = 2.0 Hz), 6.83–6.81 (2H, m), 5.20, (8H, s), 3.50 (12H, s); ^{13}C-NMR (100 MHz, $CDCl_3$) δ 185.1, 158.6, 158.2, 145.7, 136.6, 135.3, 134.9, 131.1, 121.0, 112.2, 109.9, 107.3, 107.0, 94.6, 94.5, 56.18, 56.16; LR-MS (FAB) *m/z* 508 (M^+), 45 (100%); HR-MS (FAB) Calcd. for $C_{25}H_{29}O_9Cl$: 508.1500, found: 508.1504.

GO-Y169

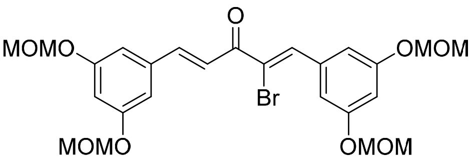

To a solution of GO-Y163 (3.66 mg, 0.00577 mmol) in dry DMF (0.57 mL) was added Li_2CO_3 (0.9 mg, 0.01 mmol). After being stirred for 1.75 h at 120 °C, the reaction was quenched with saturated aqueous NH_4Cl, and extracted with Et_2O. The combined organic layers were dried over $MgSO_4$, and concentrated *in vacuo*. The residue was purified by silica gel column chromatography (EtOAc/Hexane = 1/3 to 1/2) to give GO-Y169 (3.01 mg, 0.00543 mmol, 94%) as a yellow amorphous.

Yellow amorphous (AcOEt:hexane = 1:6) mp 79–82 °C; IR ($CHCl_3$): 2954, 1662, 1589, 1448 cm^{-1}; ^{1}H-NMR (600 MHz, $CDCl_3$) δ 7.99 (1H, s), 7.70 (1H, d, J = 15.7 Hz), 7.47 (1H, d, J = 15.7 Hz), 7.25 (2H, d, J = 2.1 Hz), 6.97 (2H, d, J = 2.1 Hz), 6.828 (1H, t, J = 2.1 Hz), 6.821 (1H, t, J = 2.1 Hz), 5.20 (8H, s), 3.50 (12H, s); ^{13}C-NMR (150 MHz, $CDCl_3$) δ 185.5, 158.6, 158.1, 145.6, 138.9, 136.6, 135.7, 123.8, 121.7, 111.6, 109,9, 107.3, 107.0, 94.6, 94.5, 56.2, 56.1; LR-MS (EI) *m/z* 552 (M^+), 45 (100%); HR-MS (EI) Calcd. for $C_{25}H_{29}O_9Br$: 552.0995, found: 552.0957.

GO-Y170

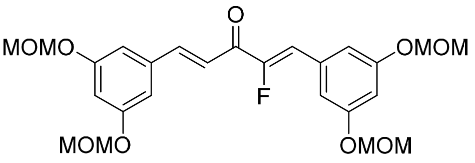

To a solution of silyl enol ether **S5** (0.01 M in THF, 8 mL, 0.080 mmol) was added NBS (17.6 mg, 0.0992 mmol) at 0 °C. After being stirred for 20 min at 0 °C, the mixture was quenched with saturated aqueous $NaHCO_3$. The resulting solution was extracted with AcOEt. The combined organic extracts were washed with brine, dried over $MgSO_4$ and concentrated *in vacuo*. The residue was purified by flash silica gel column chromatography (EtOAc/Hexane = 1/4) to give bromoketone **9** (43.0 mg,

0.0776 mmol) as a crude oil. To a solution of bromoketone **9** (0.0776 mmol) in dry THF (3 mL) was added TESCl (0.13 mL, 0.776 mmol) and LHMDS (0.59 mL of 1.3 M solution in THF, 0.776 mmol) at −40 °C. After 30 min, the mixture was quenched with Et_3N (Pasteur pipette 6 drops) and saturated aqueous $NaHCO_3$. The resulting solution was extracted with AcOEt. The combined organic extracts were washed with brine, dried over $MgSO_4$ and concentrated *in vacuo*. The residue was purified by flash silica gel column chromatography (EtOAc/Hexane = 1/6 to 1/4) to give α-bromo silyl enol ether (41.6 mg, 0.0621 mmol). To a Selectfluor® (87.9 mg, 0.248 mmol) was added resultant α-bromo silyl enol ether (41.6 mg, 0.0621 mmol) in dry DMF (5 mL) at 0 °C. After being stirred for 5 h at room temperature, the reaction mixture was quenched with saturated aqueous $NaHCO_3$. The resulting solution was extracted with AcOEt. The combined organic extracts were washed with brine, dried over $MgSO_4$ and concentrated *in vacuo*. The residue was purified by flash silica gel column chromatography (EtOAc/Hexane = 1/4 to 1/2) to give α-bromo α-fluoro enone **10** (29.5 mg, 0.0514 mmol). To a solution of resultant α-bromo α-fluoro enone **10** (29.5 mg, 0.0514 mmol) in dry DMF (5 mL) was added Li_2CO_3 (7.6 mg, 0.102 mmol). After being stirred for 1.75 h at 120 °C, the reaction mixture was quenched with saturated aqueous NH_4Cl. The resulting solution was extracted with AcOEt. The combined organic extracts were washed with brine, dried over $MgSO_4$ and concentrated *in vacuo*. The residue was purified by preparative TLC (EtOAc/toluene = 1/17, thrice) to give GO-Y170 (14.6 mg, 0.0296 mmol, 64% for 4 steps) as a yellow amorphous.

Yellow amorphous; mp 80–83 °C; IR ($CHCl_3$): 2956, 1680, 1590, 1440 cm^{-1}; ^{1}H-NMR (600 MHz, $CDCl_3$) δ7.78 (1H, d, J = 15.7 Hz), 7.33 (1H, dd, J = 15.7, 2.7 Hz), 7.08 (2H, d, J = 2.2 Hz), 6.98 (2H, d, J = 2.1 Hz), 6.89 (1H, d, $^3J_{HF}$ = 36.2 Hz), 6.82 (1H, t, J = 2.2 Hz), 6.79 (1H, t, J = 2.1 Hz), 5.20 (4H, s), 5.19 (4H, s), 3.50 (12H, s); ^{13}C-NMR (150 MHz, $CDCl_3$) δ 183.1 (d, $^2J_{CF}$ = 31.5 Hz), 158.6, 158.4, 155.0 (d, $^1J_{CF}$ = 275.1 Hz), 145.5, 136.5, 133.1 ($^3J_{CF}$ = 3.0 Hz), 120.1, 115.9 ($^2J_{CF}$ = 4.3 Hz), 112.0 (d, $^4J_{CF}$ = 8.6 Hz), 110.0, 107.6, 106.8, 94.6, 94.5, 56.18, 56.16; LR-MS (FAB) *m*/*z* 492 (M^+), 45 (100%); HR-MS (FAB) Calcd. for $C_{25}H_{29}O_9F$: 492.1796, found: 492.1800.

GO-Y171

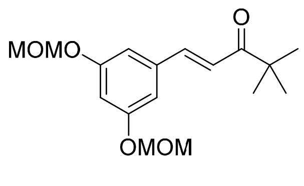

Colorless oil; IR ($CHCl_3$): 2965, 1684, 1610, 1589, 1477, 1283 cm^{-1}; ^{1}H-NMR (400 MHz, $CDCl_3$) δ 7.58 (1H, J = 15.6 Hz), 7.06 (1H, d, J = 15.6 Hz), 6.90 (2H, d, J = 2.0 Hz), 6.79–6.77 (1H, m), 5.18 (4H, s), 3.49 (6H, s), 1.22 (9H, s); ^{13}C-NMR (100 MHz, $CDCl_3$) δ 203.9, 158.4, 142.5, 136.9, 121.3, 109.5, 106.5, 94.4, 56.0, 43.1, 26.2; LR-MS (EI) *m*/*z* 308 (M^+), 45 (100%); HR-MS (EI) Calcd. for $C_{17}H_{24}O_5$: 308.1624, found: 308.1610.

GO-Y172

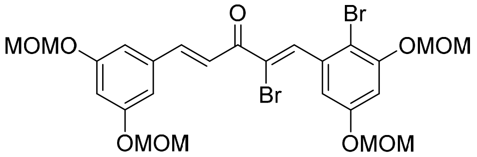

Yellow amorphous; mp 49–51 °C; IR ($CHCl_3$): 2956, 2827, 1666, 1588, 1436, 1398 cm^{-1}; ^1H-NMR (600 MHz, $CDCl_3$) δ 8.11 (1H, s), 7.73 (1H, d, J = 15.7 Hz), 7.42 (1H, d, J = 15.7 Hz), 7.23 (1H, d, J = 2.7 Hz), 6.97 (2H, d, J = 2.0 Hz), 6.93 (1H, d, J = 2.7 Hz), 6.81 (1H, t, J = 2.0 Hz), 5.26 (2H, s), 5.19 (4H, s), 5.18 (2H, s), 3.54 (3H, s), 3.49 (9H, s); ^{13}C-NMR (150 MHz, $CDCl_3$) δ 185.5, 158.6, 156.8, 154.7, 145.9, 139.1, 136.5, 136.4, 126.5, 121.7, 111.4, 109.9, 107.4, 107.0, 106.0, 95.3, 94.8, 94.5, 56.5, 56.20, 56.17; LR-MS (FAB) *m/z* 630 (M^+), 45 (100%), 153 (100%); HR-MS (FAB) Calcd. for $C_{25}H_{28}O_9Br_2$: 630.0100, found: 630.0110.

Compound Data (Other)

3,5-Bis-[2-(tetrahydro-pyran-2-yloxy)-ethoxy]-benzaldehyde (**S6**)

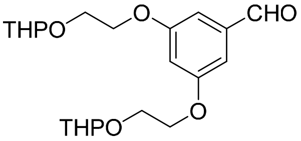

Colorless oil; IR (neat): 2941, 2872, 1698, 1593, 1449 cm^{-1}; ^1H-NMR (400 MHz, $CDCl_3$) δ 9.89 (1H, s), 7.05 (2H, d, J = 2 Hz), 6.80 (1H, t, J = 2.2 Hz), 4.71 (2H, t, J = 3.4 Hz), 4.21–4.18 (4H, m), 4.06 (2H, dt, J = 11.2, 5.1 Hz), 3.89–3.87 (2H, m), 3.85–3.81 (2H, m), 3.55–3.52 (2H, m), 1.87–1.79 (2H, m), 1.78–1.71 (2H, m), 1.67–1.51 (8H, m); ^{13}C-NMR (100 MHz, $CDCl_3$) δ 191.9, 160.5, 138.2, 108.5, 108.0, 99.0, 67.8, 65.6, 62.2, 30.5, 25.4, 19.3; LR-MS (EI) *m/z* 394 (M^+), 85 (100%); HR-MS (EI) Calcd. for $C_{22}H_{30}O_2$: 394.1992, found: 394.1971.

3,5-Bis-(2-methoxyethoxy)-benzaldehyde (**S7**)

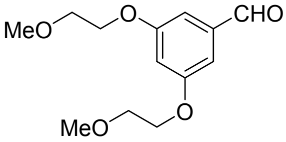

Colorless oil; IR ($CHCl_3$): 2930, 2730, 1697, 1593, 1448 cm^{-1}; ^1H-NMR (400 MHz, $CDCl_3$) δ 9.88 (1H, s), 7.04 (2H, d, J = 2.4 Hz), 6.79 (1H, t, J = 1.8 Hz), 4.15 (4H, t, J = 4.6 Hz), 3.76 (4H, t, J = 4.6 Hz), 3.45 (6H, s); ^{13}C-NMR (100 MHz, $CDCl_3$) δ 191.8, 160.3, 138.3, 77.0, 70.7, 67.6, 59.2; LR-MS (EI) *m/z* 254 (M^+), 58 (100%); HR-MS (EI) Calcd. for $C_{13}H_{18}O_5$: 254.1154, found: 254.1172.

4-(3,5-Bis-methoxymethoxy-phenyl)-but-3-en-2-one (**6**)

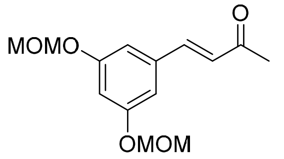

Colourless oil; IR ($CHCl_3$): 2927, 1652, 1590, 1441 cm^{-1}; ^{1}H-NMR (400 MHz, $CDCl_3$) δ 7.42 (1H, d, J = 16.8 Hz), 6.90 (2H, d, J = 2.4 Hz), 6.77 (1H, t, J = 2.2 Hz), 6.67 (1H, J = 16 Hz), 5.18 (4H, s), 3.49 (6H, s), 2.37 (3H, s); ^{13}C-NMR (100 MHz, $CDCl_3$) δ 198.3, 158.6, 143.1, 136.5, 127.7, 109.4, 107.1, 94.5, 56.1, 27.4; LR-MS (EI) *m*/*z* 266 (M^+, 100%); HR-MS (EI) Calcd. for $C_{14}H_{18}O_5$: 266.1153 found: 266.1154.

1-[2-(3,5-Bis-methoxymethoxy-phenyl)-cyclopropyl]-ethanone (**7**)

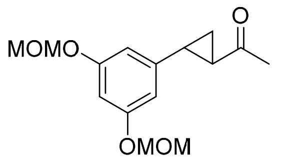

Colorless oil; IR ($CHCl_3$): 2998, 2955, 2904, 2827, 1698, 1594, 1463, 1439 cm^{-1}; ^{1}H-NMR (400 MHz, $CDCl_3$) δ 6.60–6.59 (1H, t, J = 2.0 Hz), 6.43 (2H, d, J = 2.0 Hz), 5.13 (4H, s), 3.47 (6H, s), 2.48–2.43 (1H, m), 2.29 (3H, s), 2.22–2.18 (1H, m), 1.66–1.61 (1H, m), 1.37–1.32 (1H, m); ^{13}C-NMR (100 MHz, $CDCl_3$) δ 206.5, 158.4, 142.9, 107.5, 102.9, 94.4, 56.0, 32.7, 30.7, 28.9, 19.0; LR-MS (EI) *m*/*z* 280 (M^+), 235 (100%); HR-MS (EI) Calcd. for $C_{15}H_{20}O_5$: 266.1311 found: 266.1287.

1-(Azidomethyl)-3,5-bis(methoxymethoxy)benzene (**S4**)

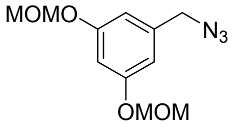

Colorless oil; IR ($CHCl_3$): 2956, 2827, 2102, 1598, 1460 cm^{-1}; ^{1}H-NMR (400 MHz, $CDCl_3$) δ 6.70 (1H, t, J = 2.1 Hz), 6.66 (2H, d, J = 2.1 Hz), 5.16 (4H, s), 4.27 (2H, s), 3.48 (6H, s); ^{13}C-NMR (100 MHz, $CDCl_3$) δ 158.6, 137.7, 109.3, 104.6, 94.5, 56.0, 54.6; LR-MS (EI) *m*/*z* 253 (M^+), 45 (100%); HR-MS (EI) Calcd. for $C_{11}H_{15}N_3O_4$: 253.1063 found: 253.1067.

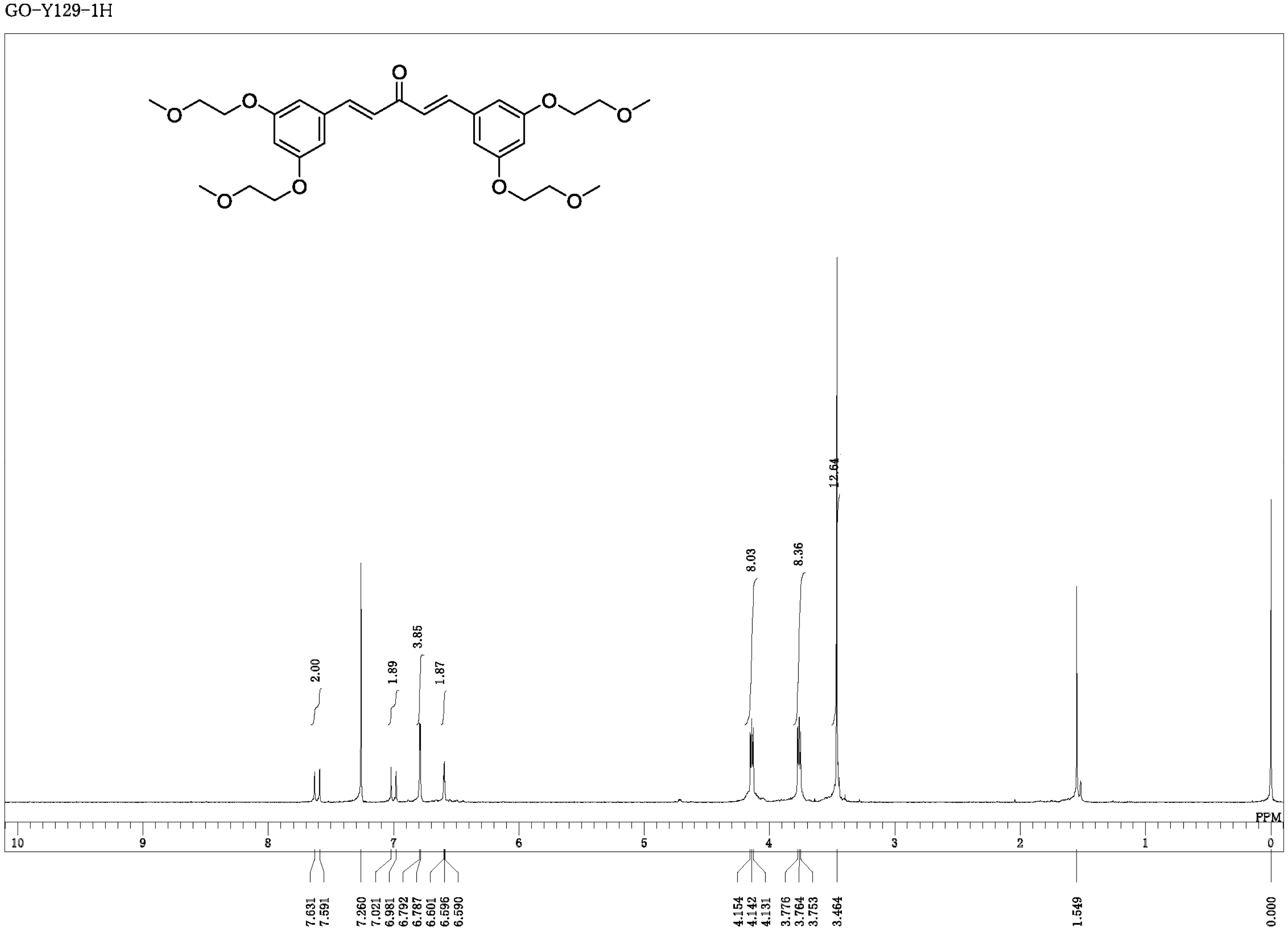

Figure S2. ^{1}H-NMR spectra of GO-Y129.

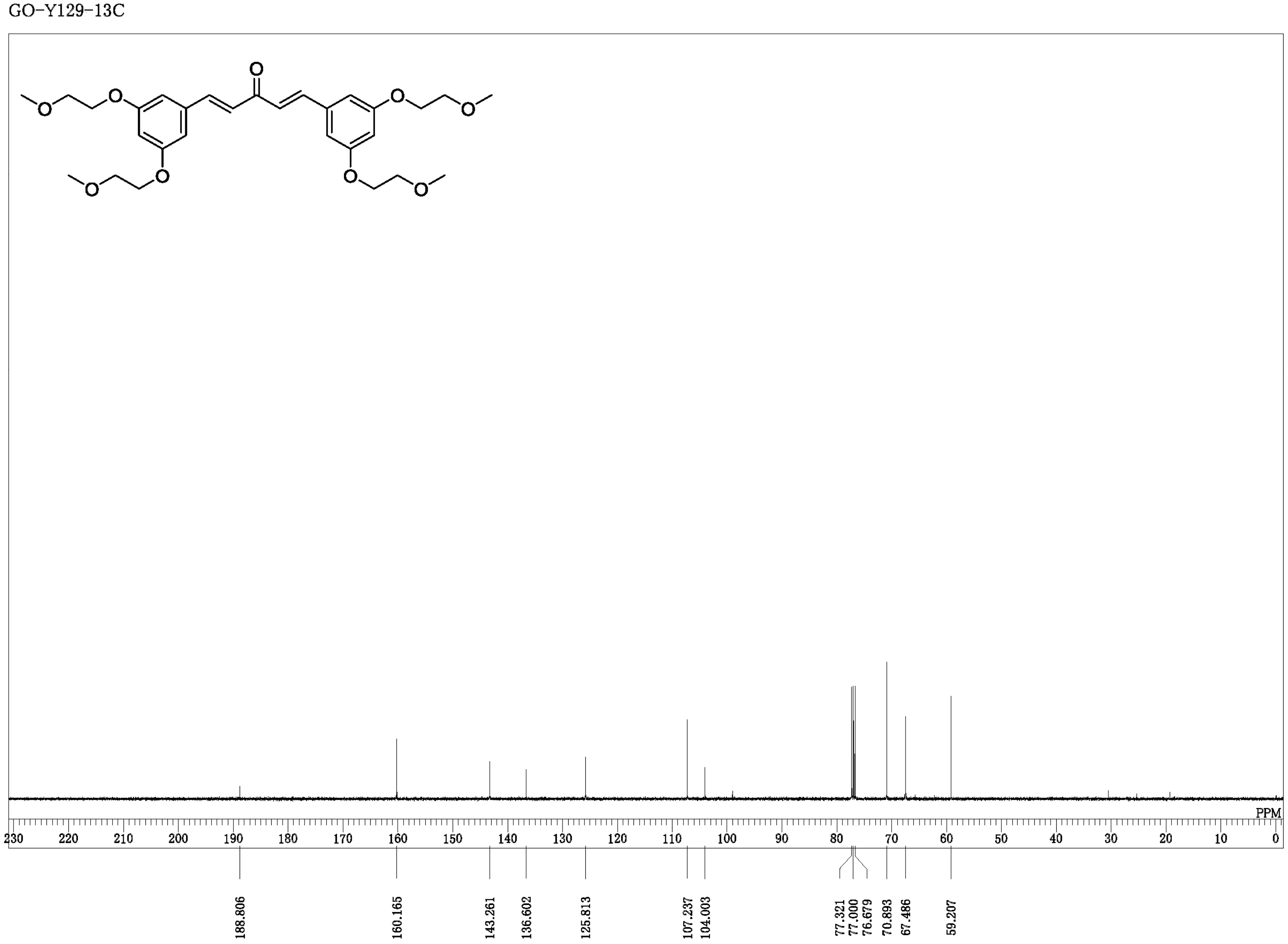

Figure S3. ^{13}C-NMR spectra of GO-Y129.

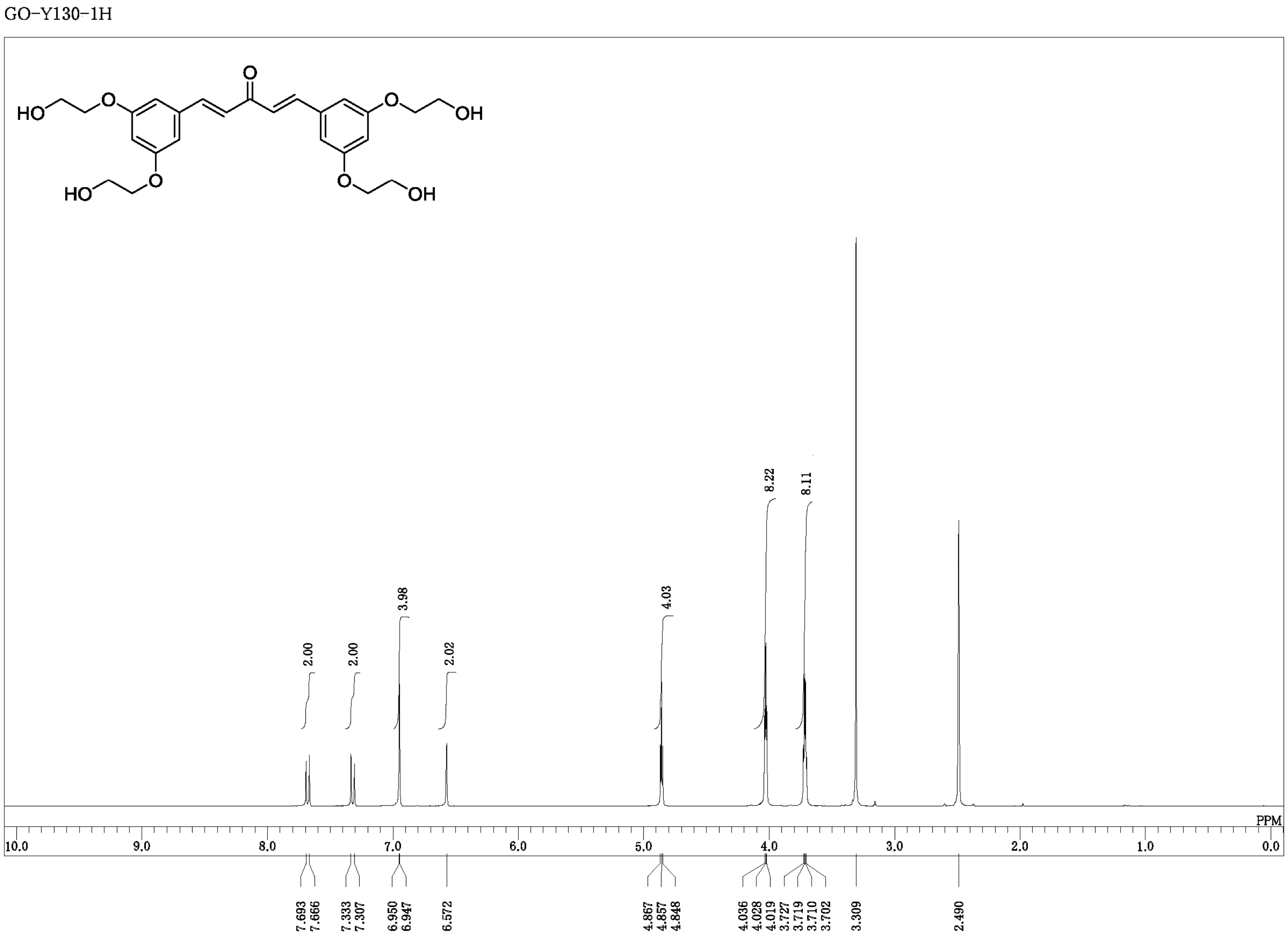

Figure S4. ^{1}H-NMR spectra of GO-Y130.

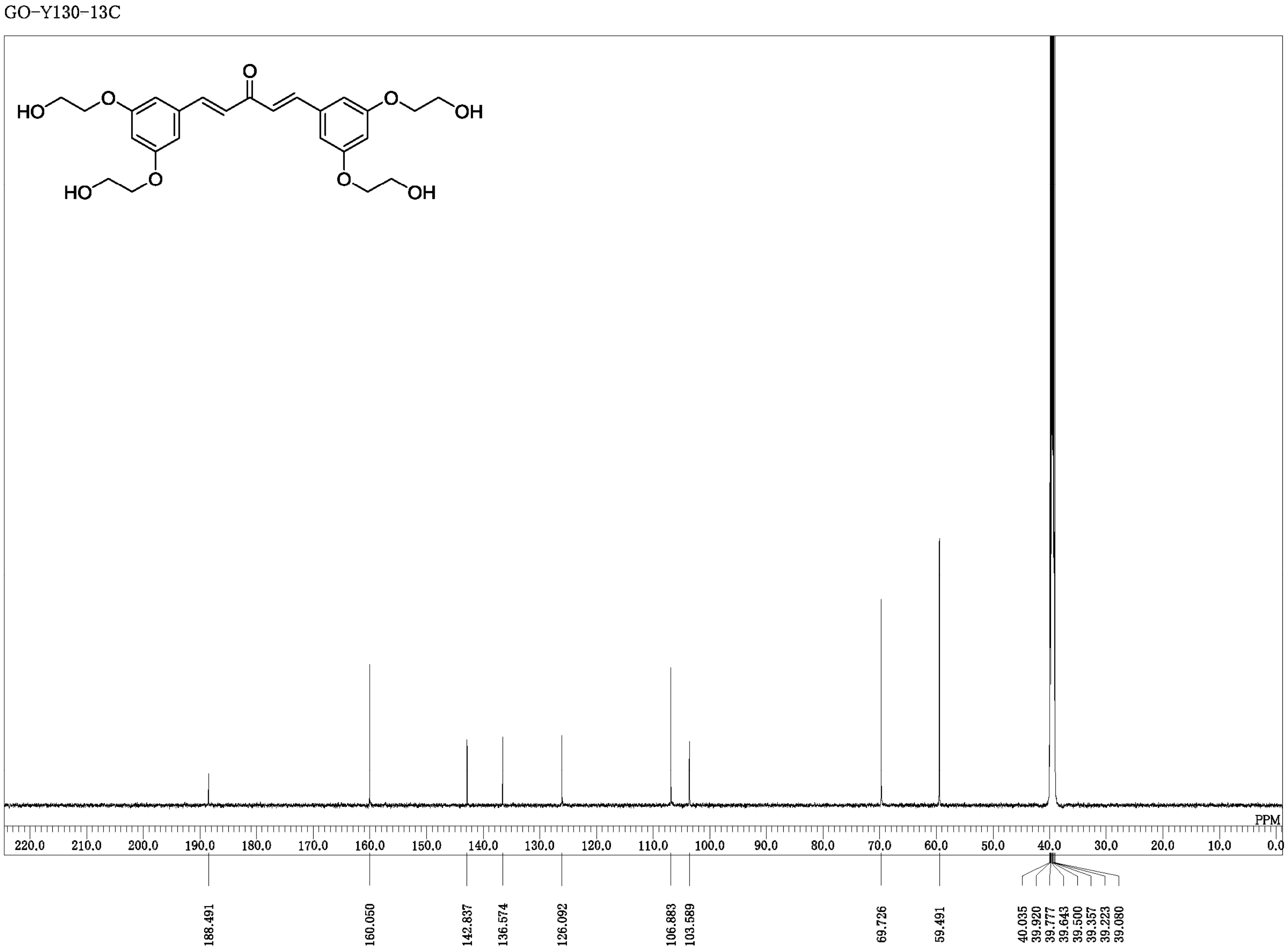

Figure S5. ^{13}C-NMR spectra of GO-Y130

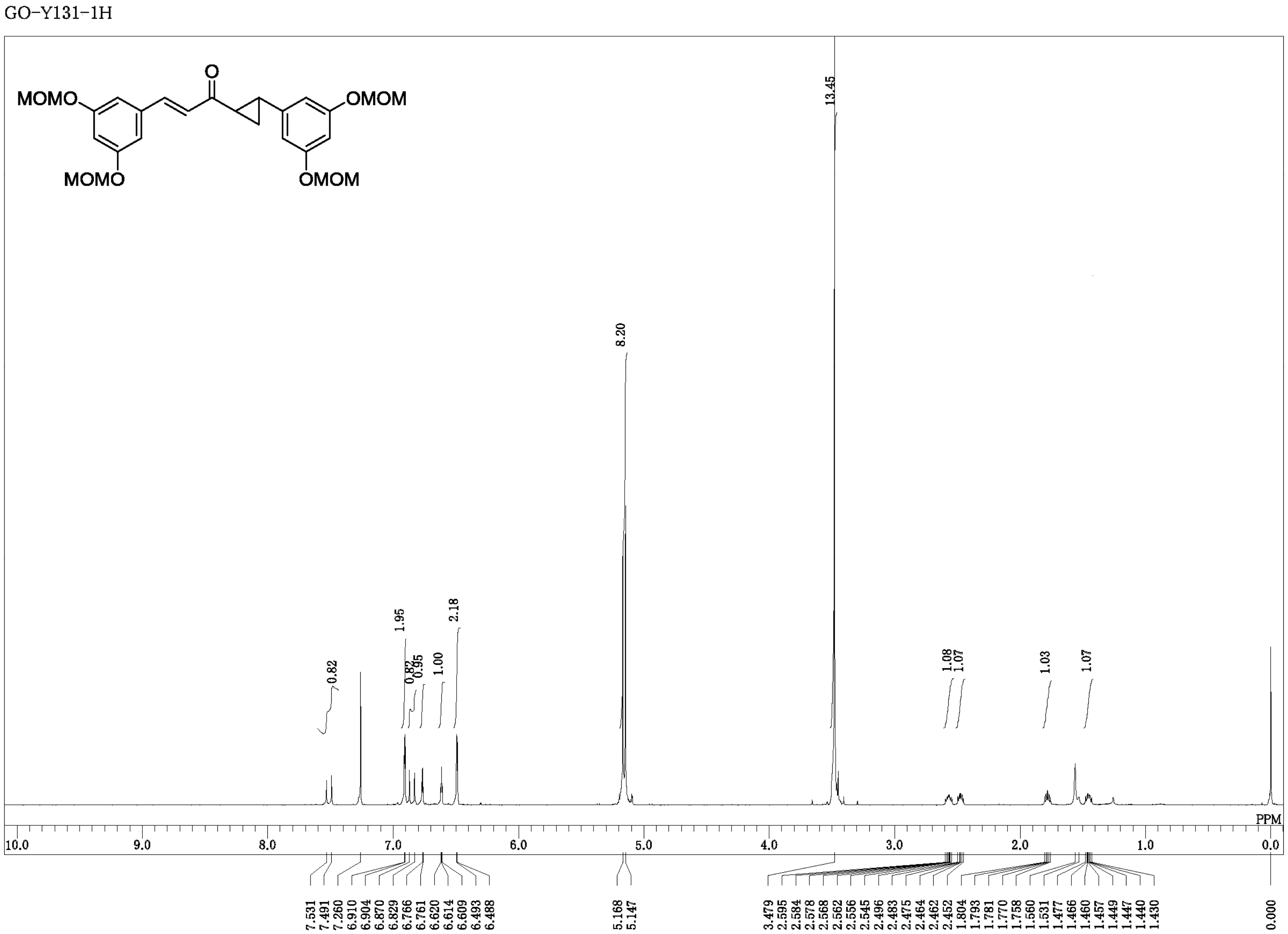

Figure S6. ^{1}H-NMR spectra of GO-Y131.

GO-Y131-13C

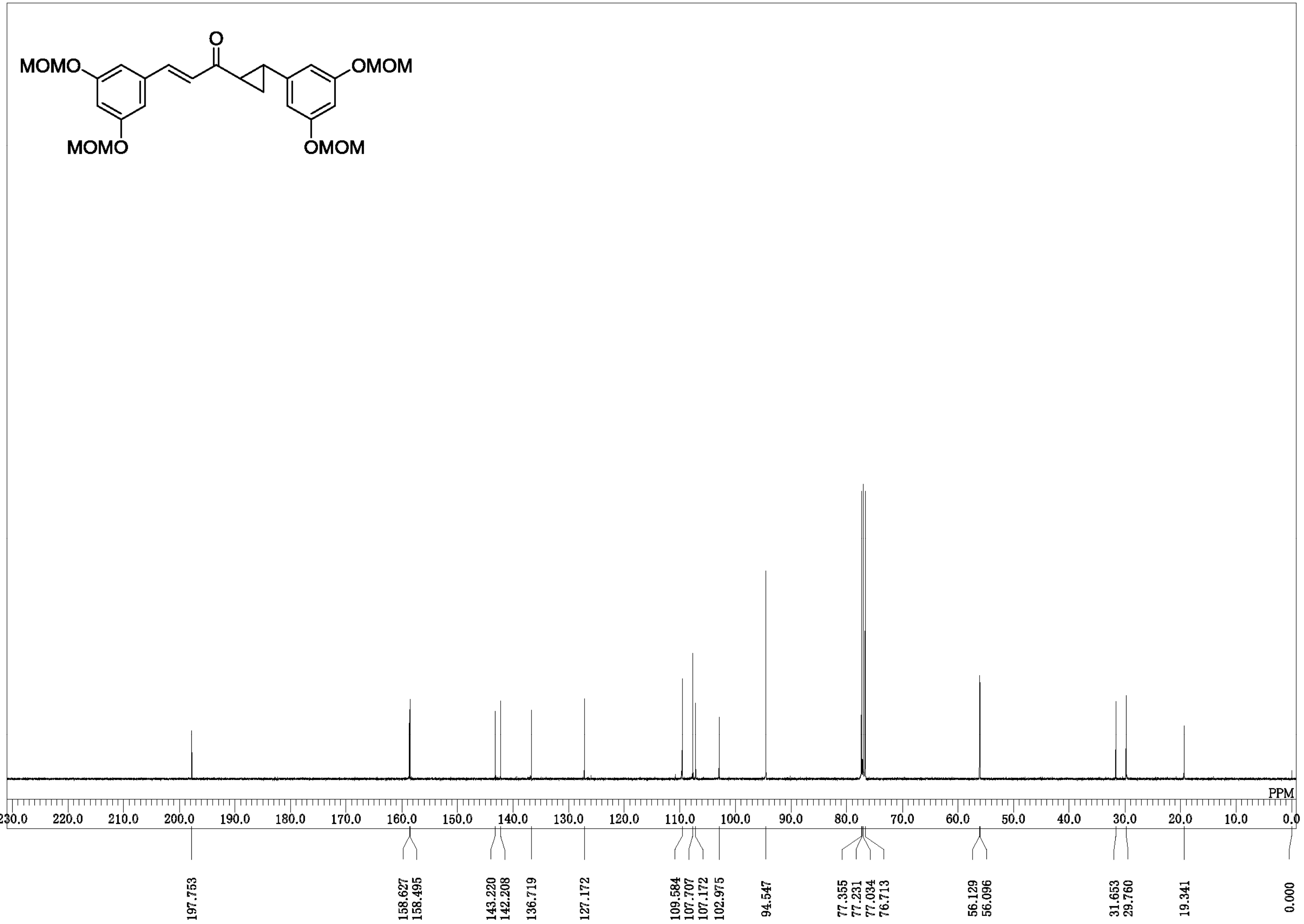

Figure S7. ^{13}C-NMR spectra of GO-Y131.

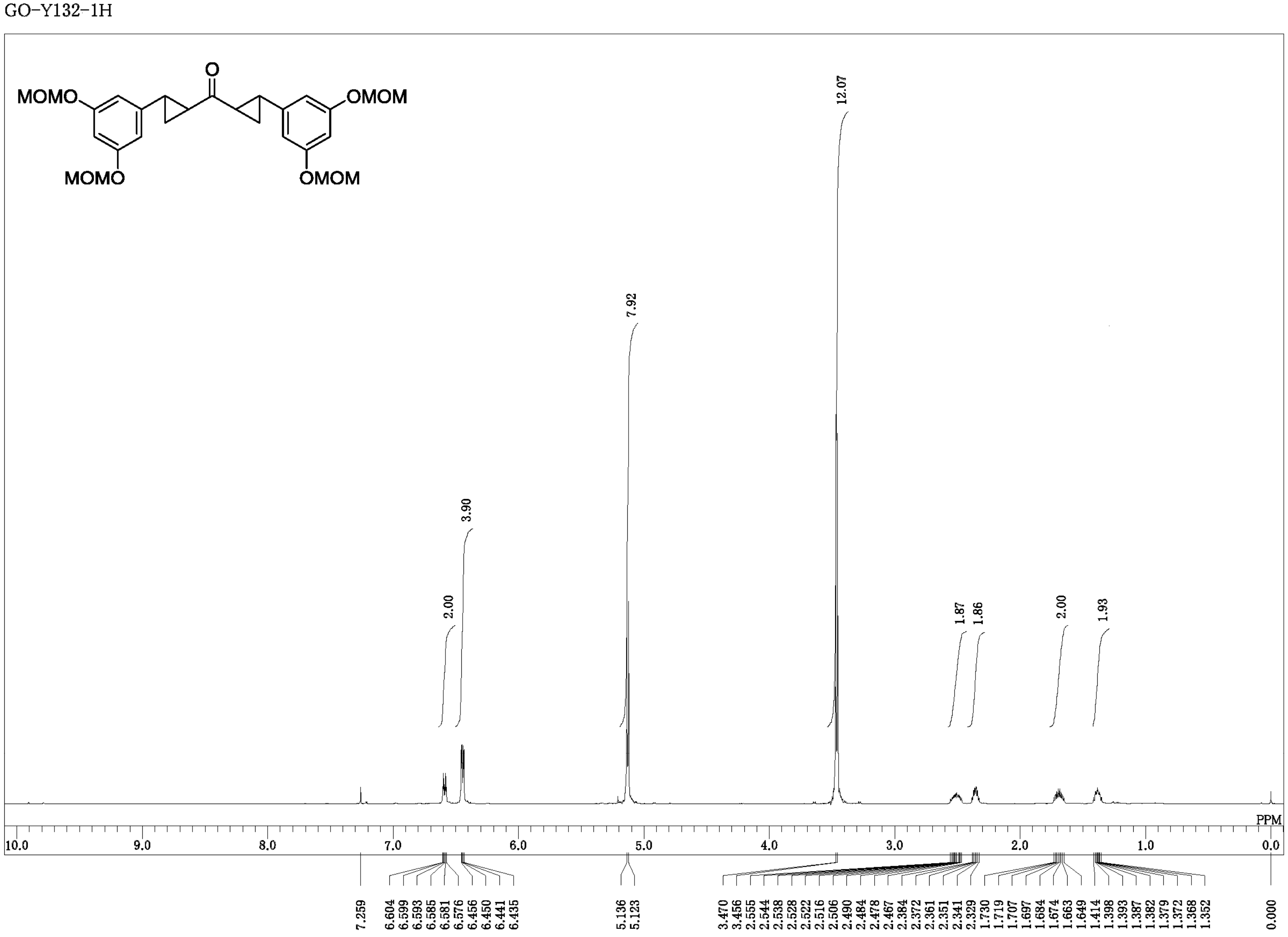

Figure S8. ^{1}H-NMR spectra of GO-Y132.

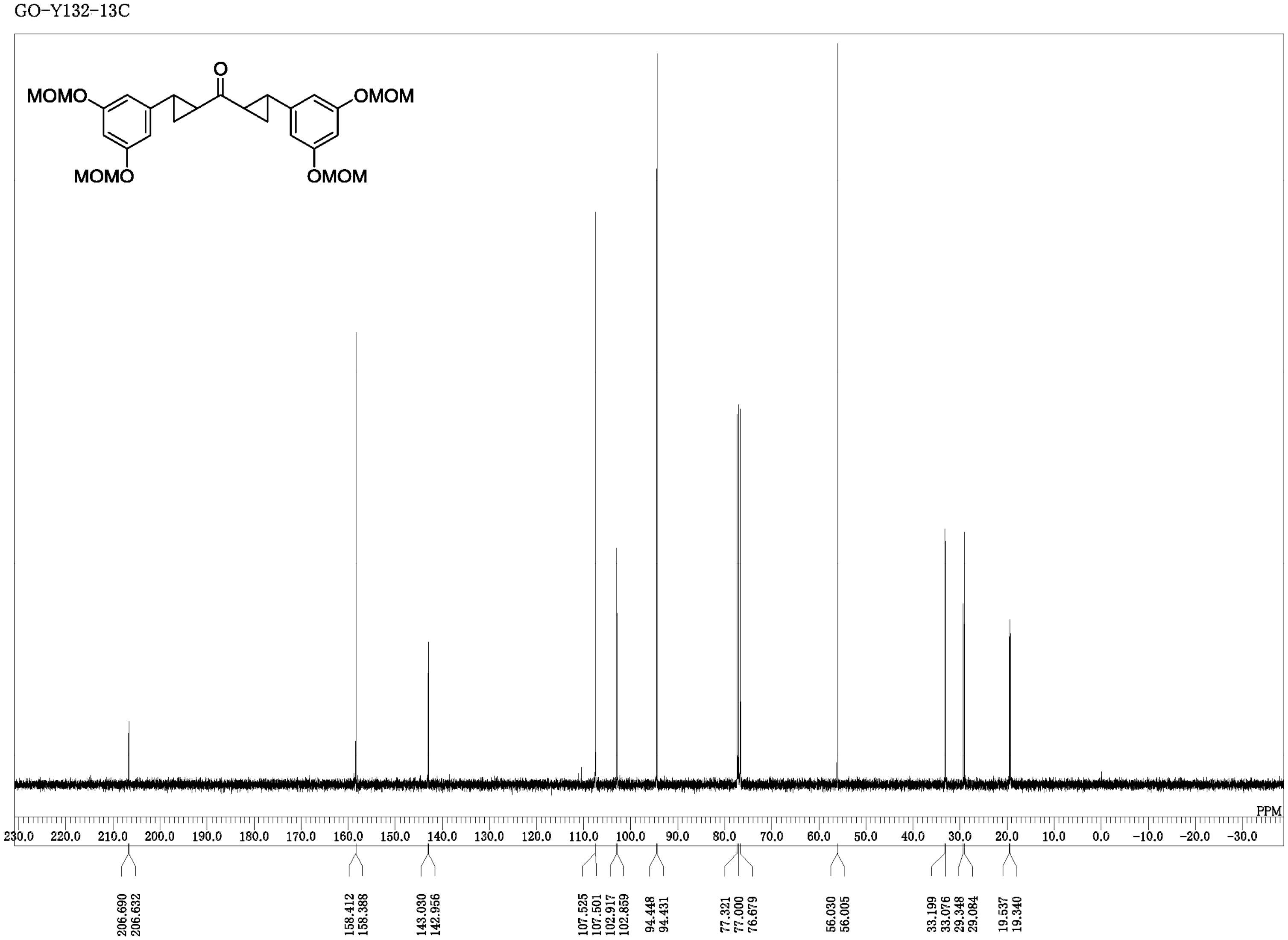

Figure S9. ^{13}C-NMR spectra of GO-Y132.

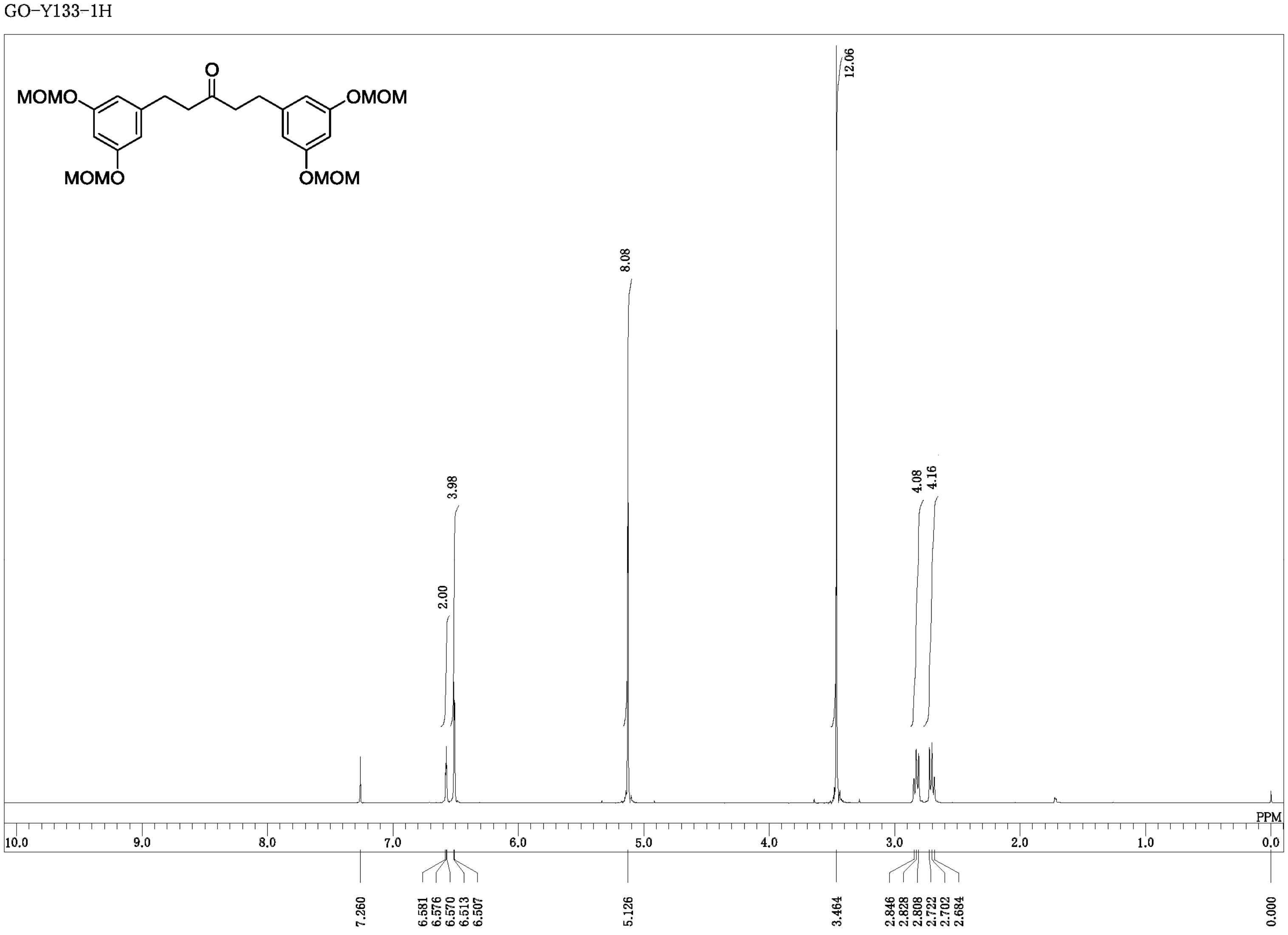

Figure S10. ^{1}H-NMR spectra of GO-Y133.

GO-Y133-13C

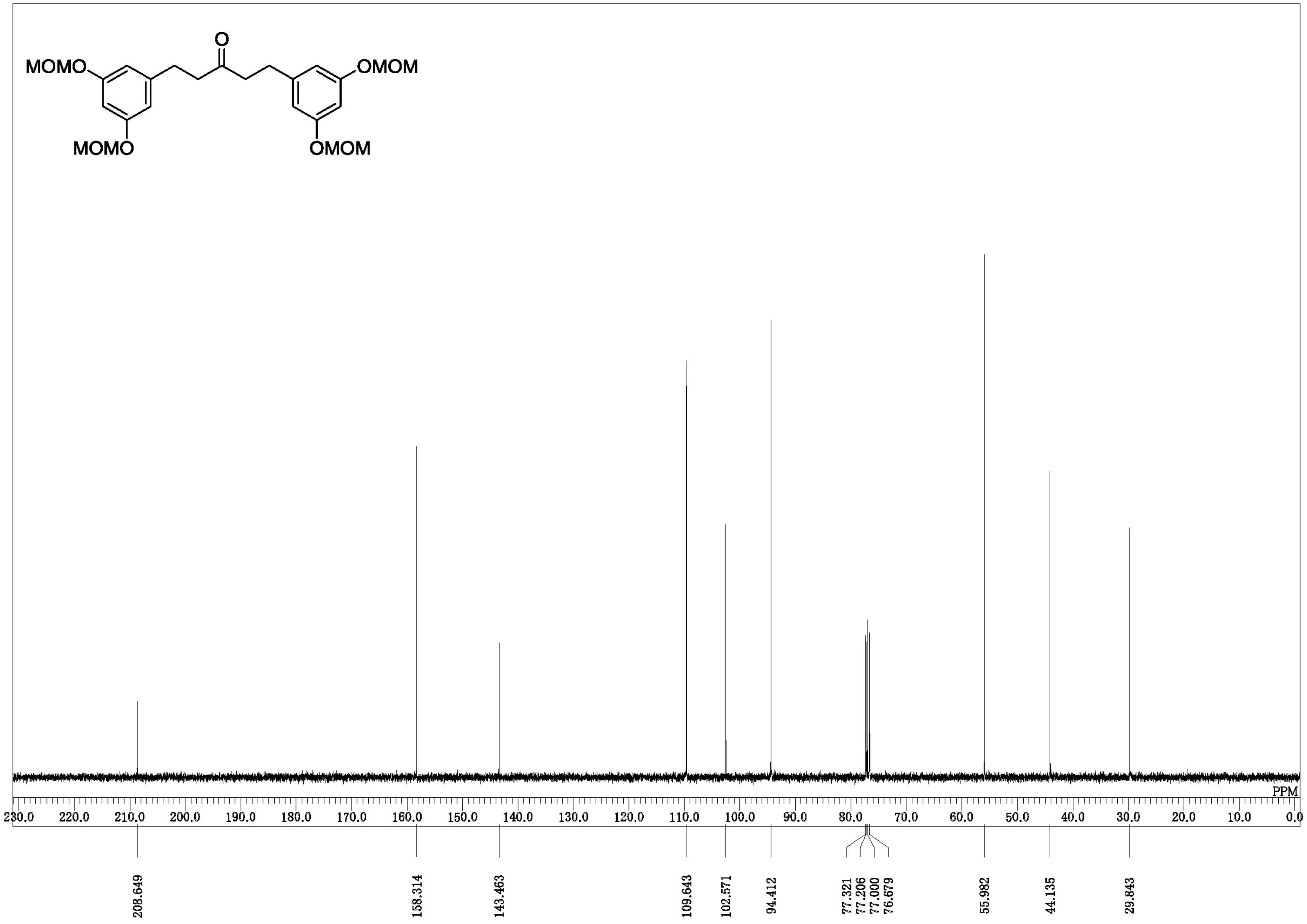

Figure S11. ^{13}C-NMR spectra of GO-Y133.

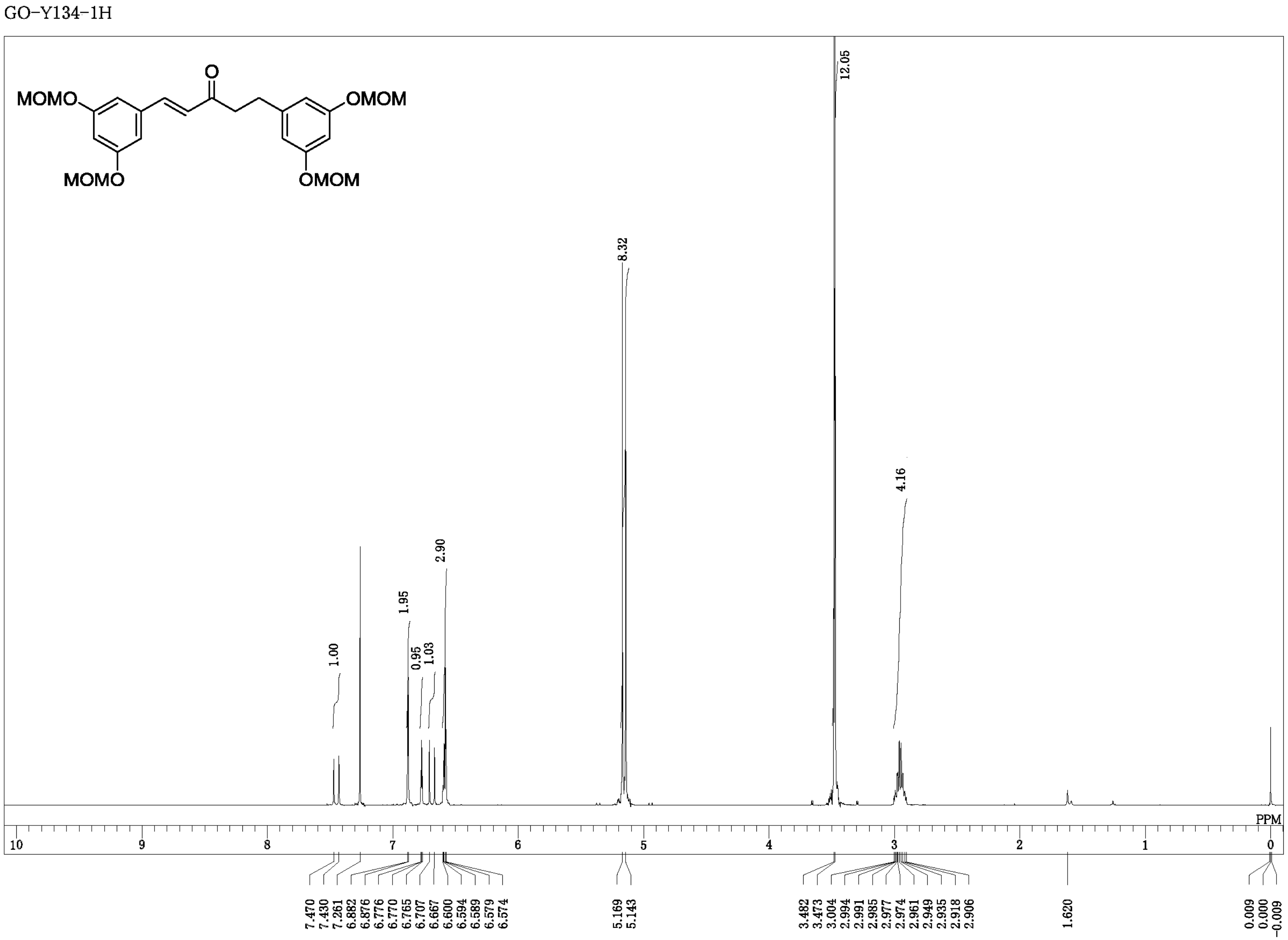

Figure S12. ^{1}H-NMR spectra of GO-Y134.

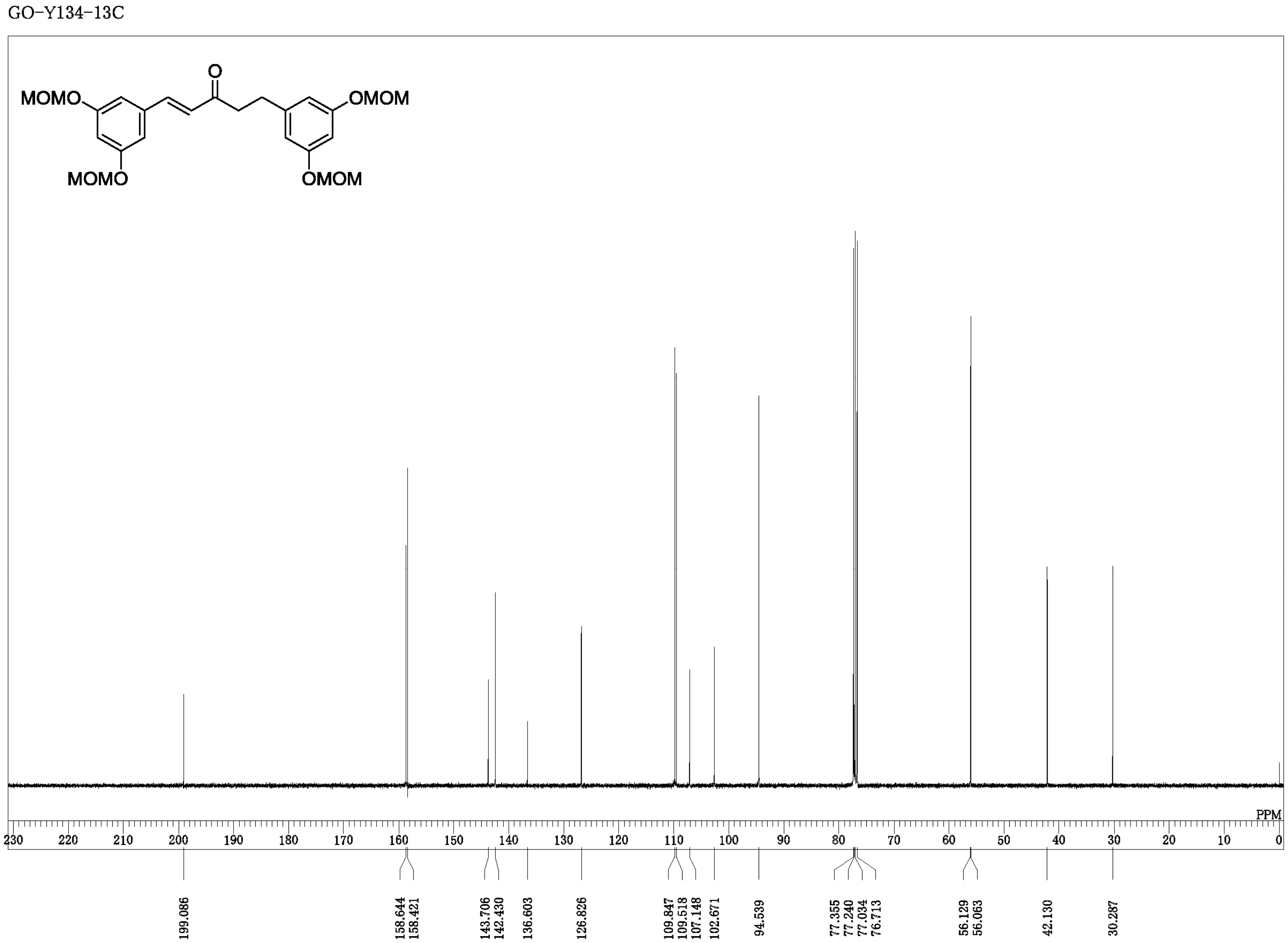

Figure S13. ^{13}C-NMR spectra of GO-Y134.

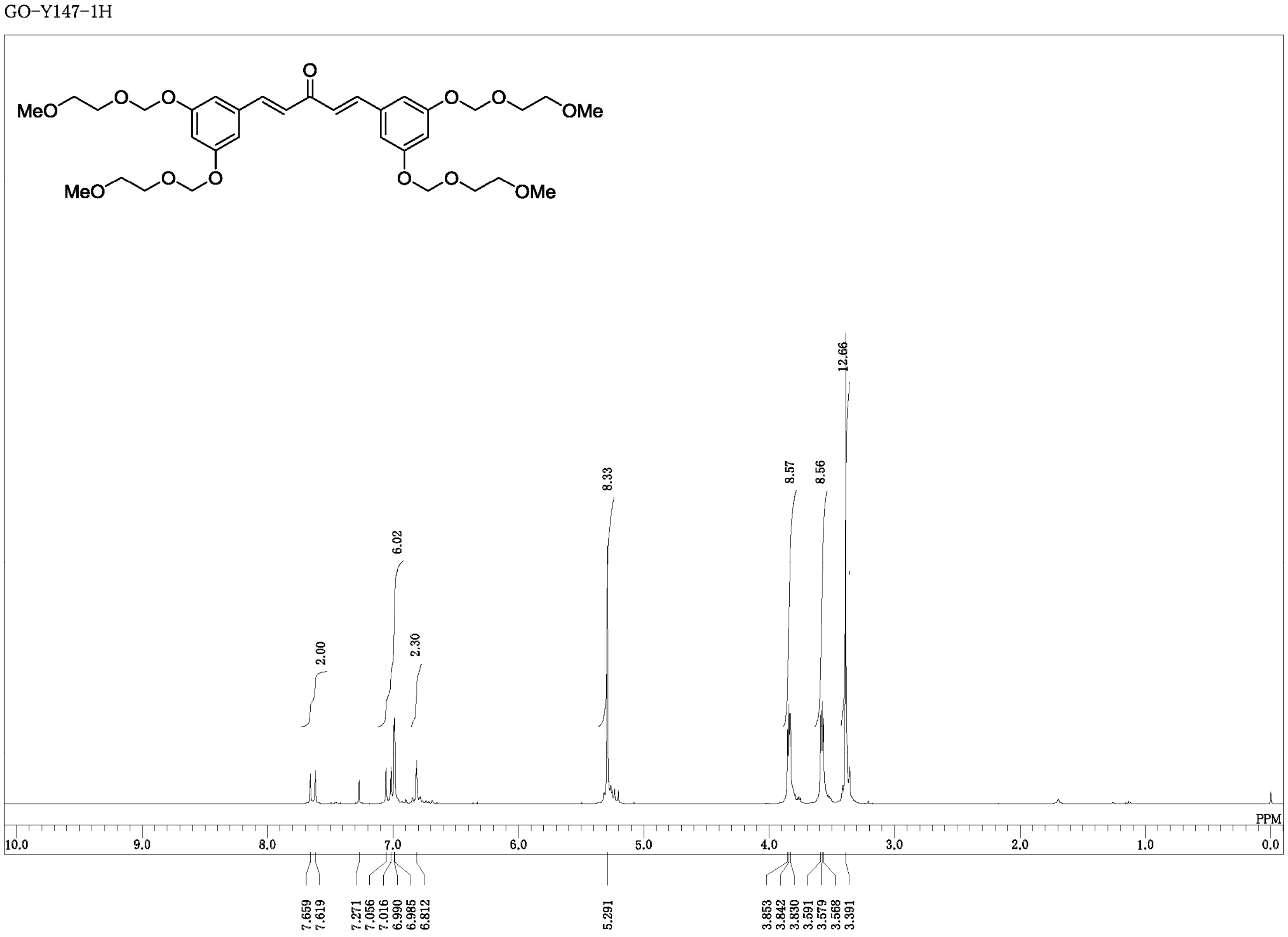

Figure S14. ^{1}H-NMR spectra of GO-Y147.

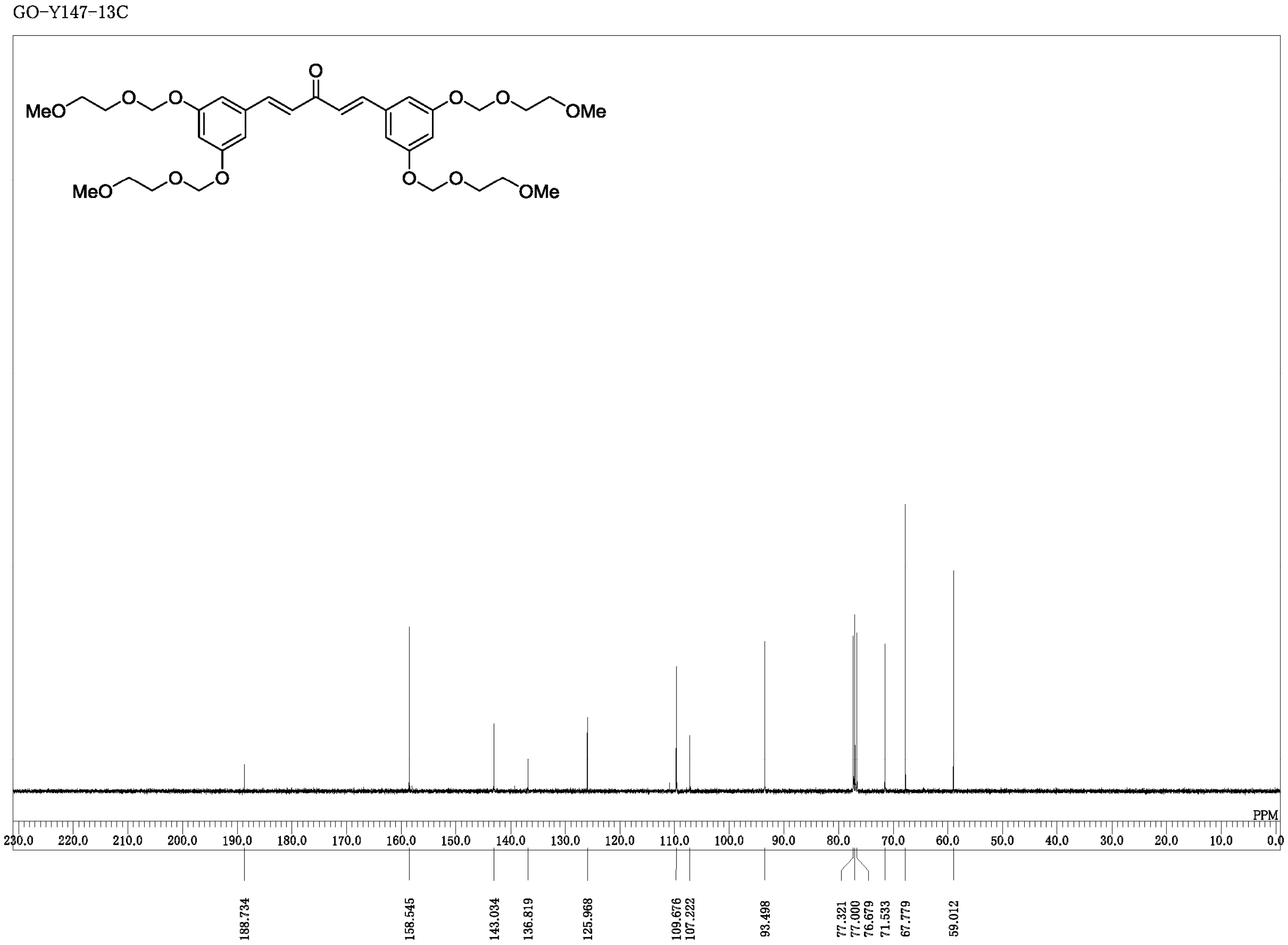

Figure S15. ^{13}C-NMR spectra of GO-Y147.

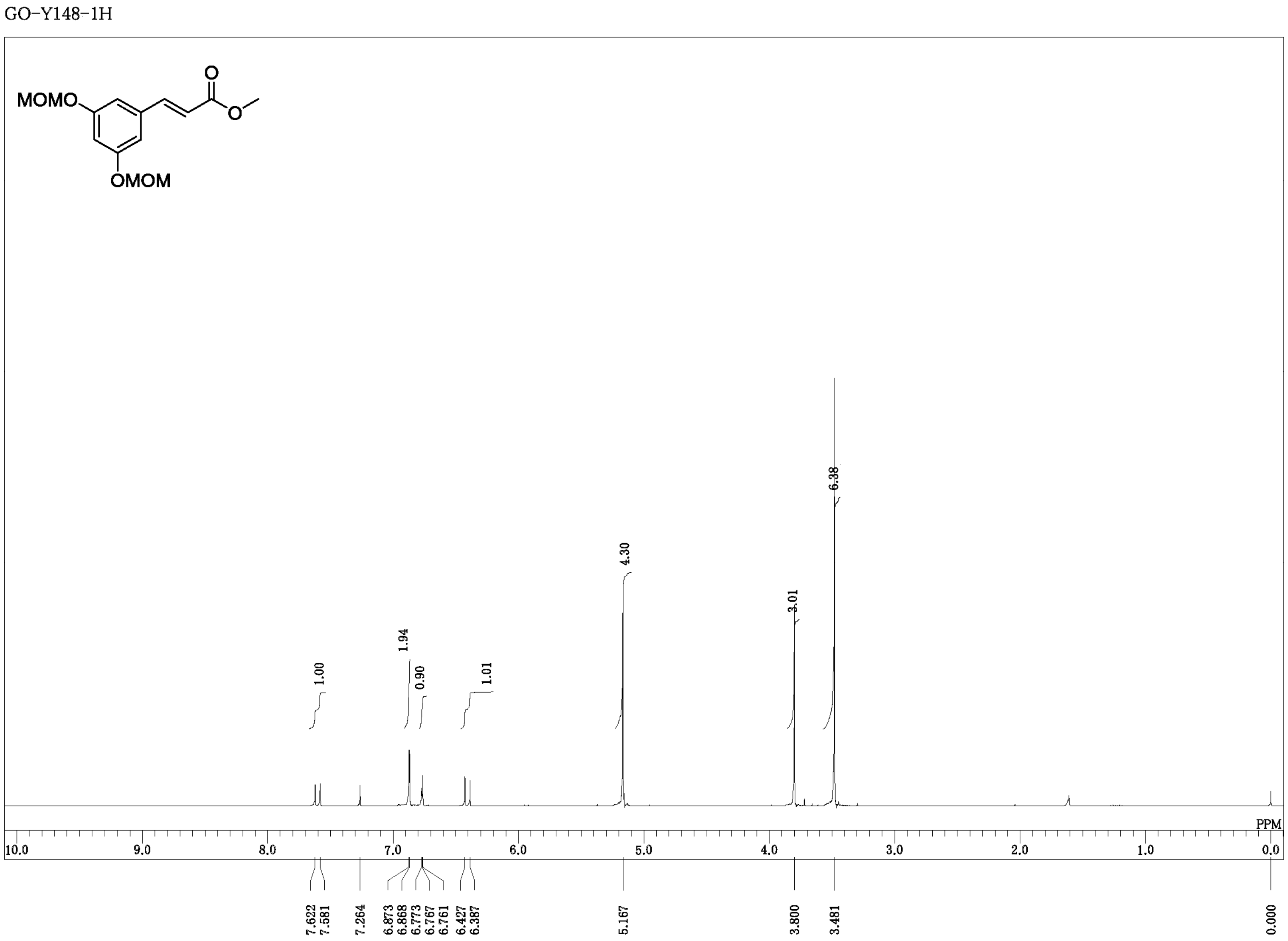

Figure S16. ^{1}H-NMR spectra of GO-Y148.

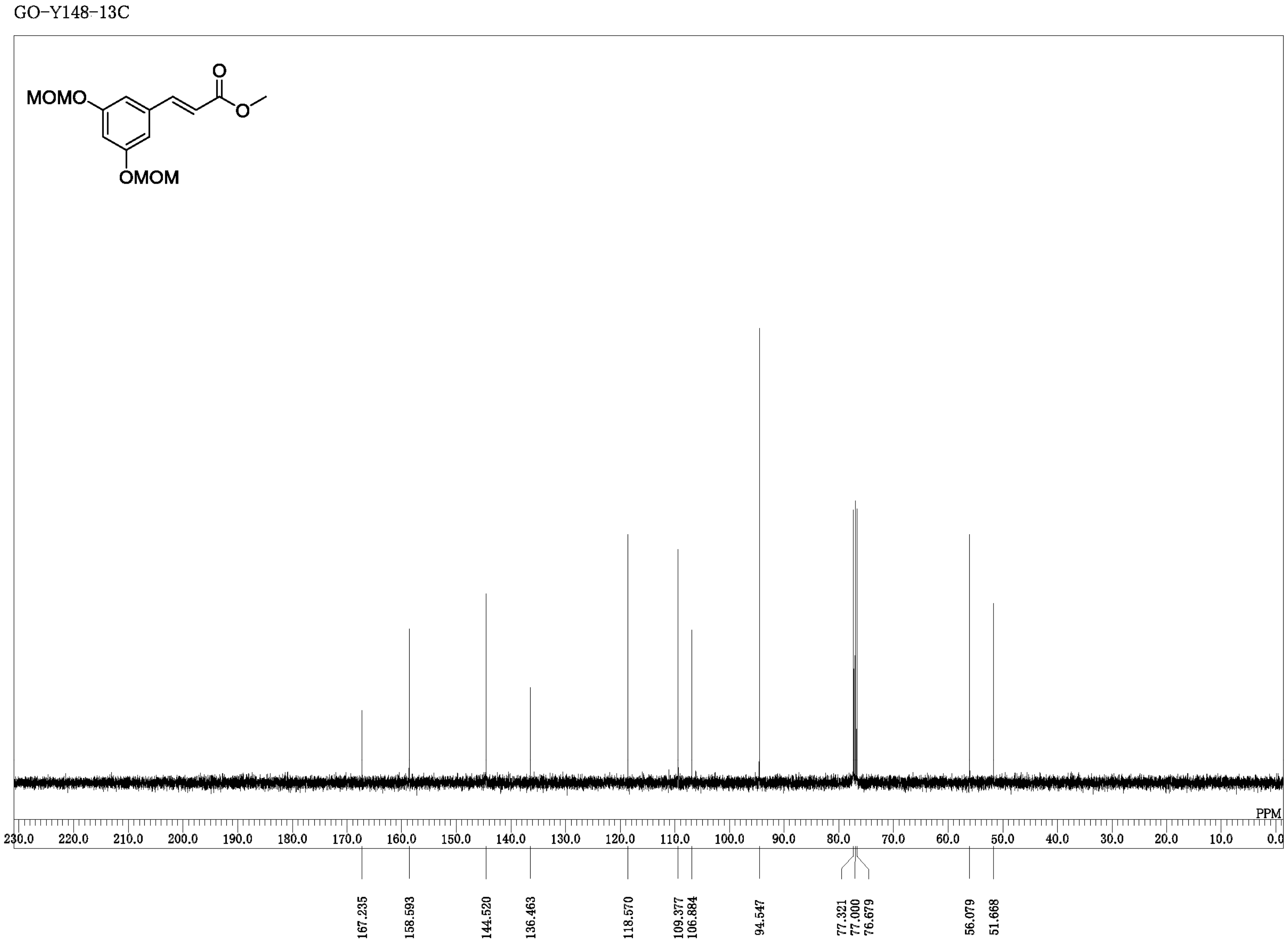

Figure S17. ^{13}C-NMR spectra of GO-Y148.

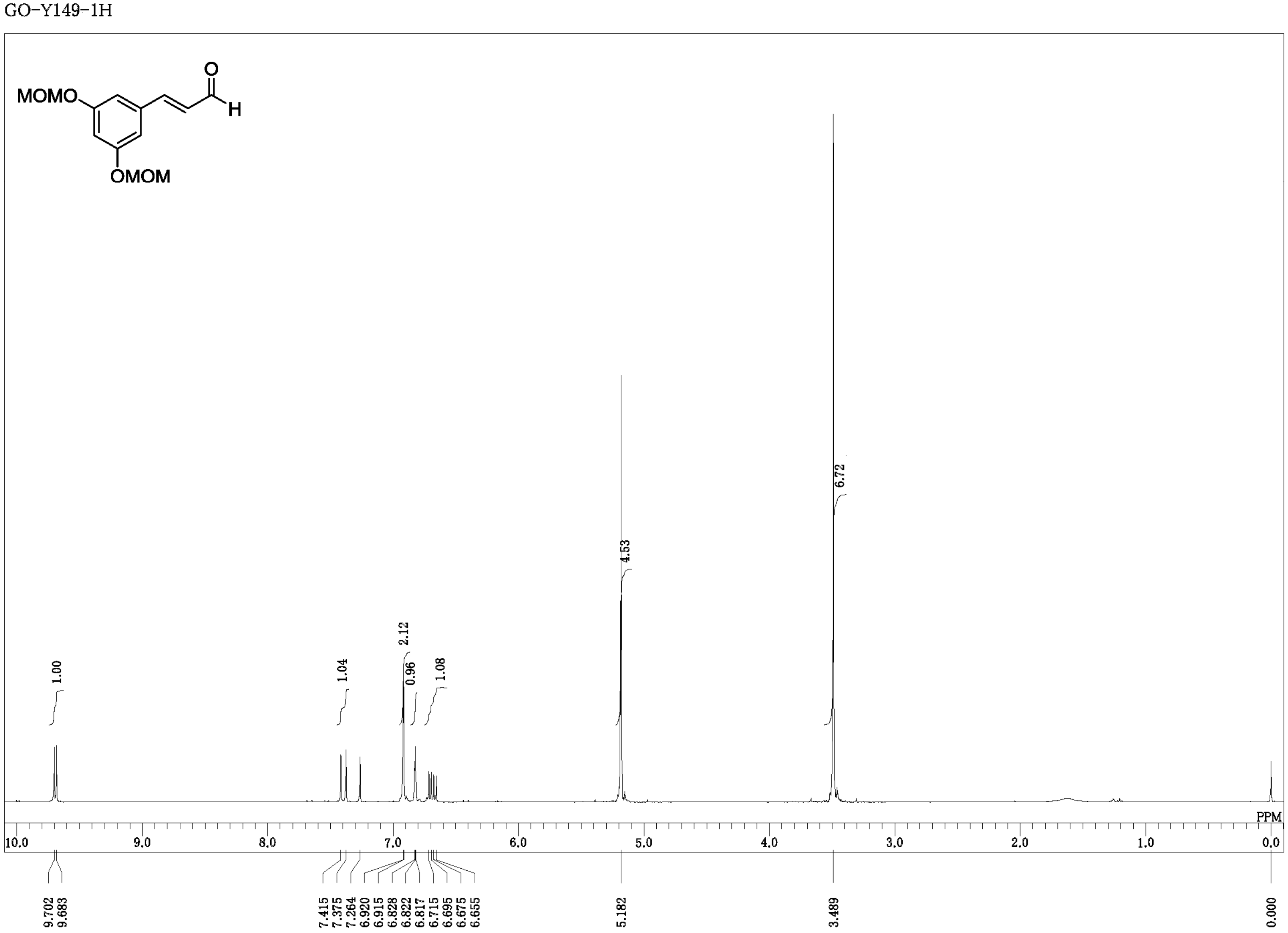

Figure S18. ^{1}H-NMR spectra of GO-Y149.

GO-Y149-13C

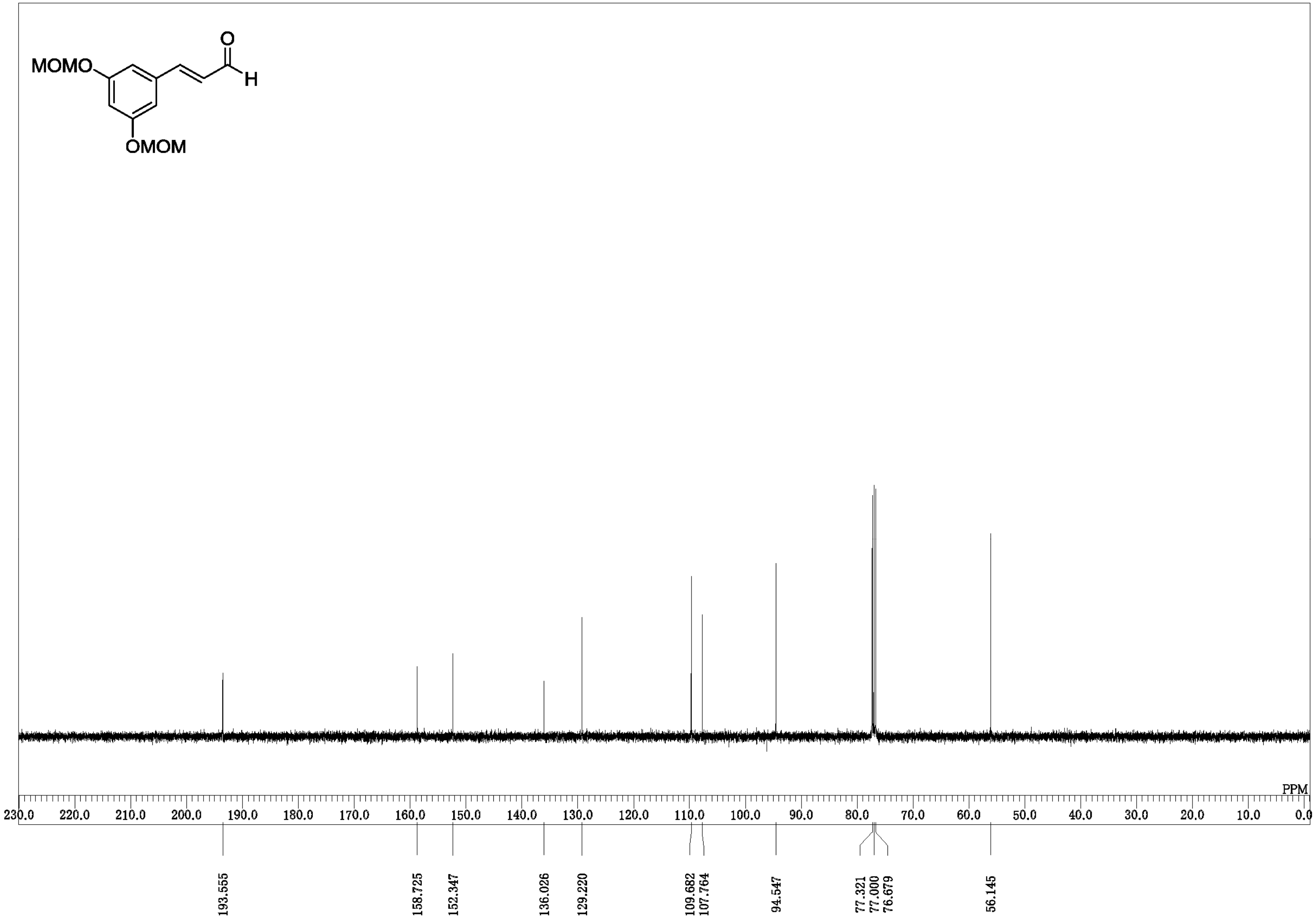

Figure S19. ^{13}C-NMR spectra of GO-Y149.

GO-Y150-1H

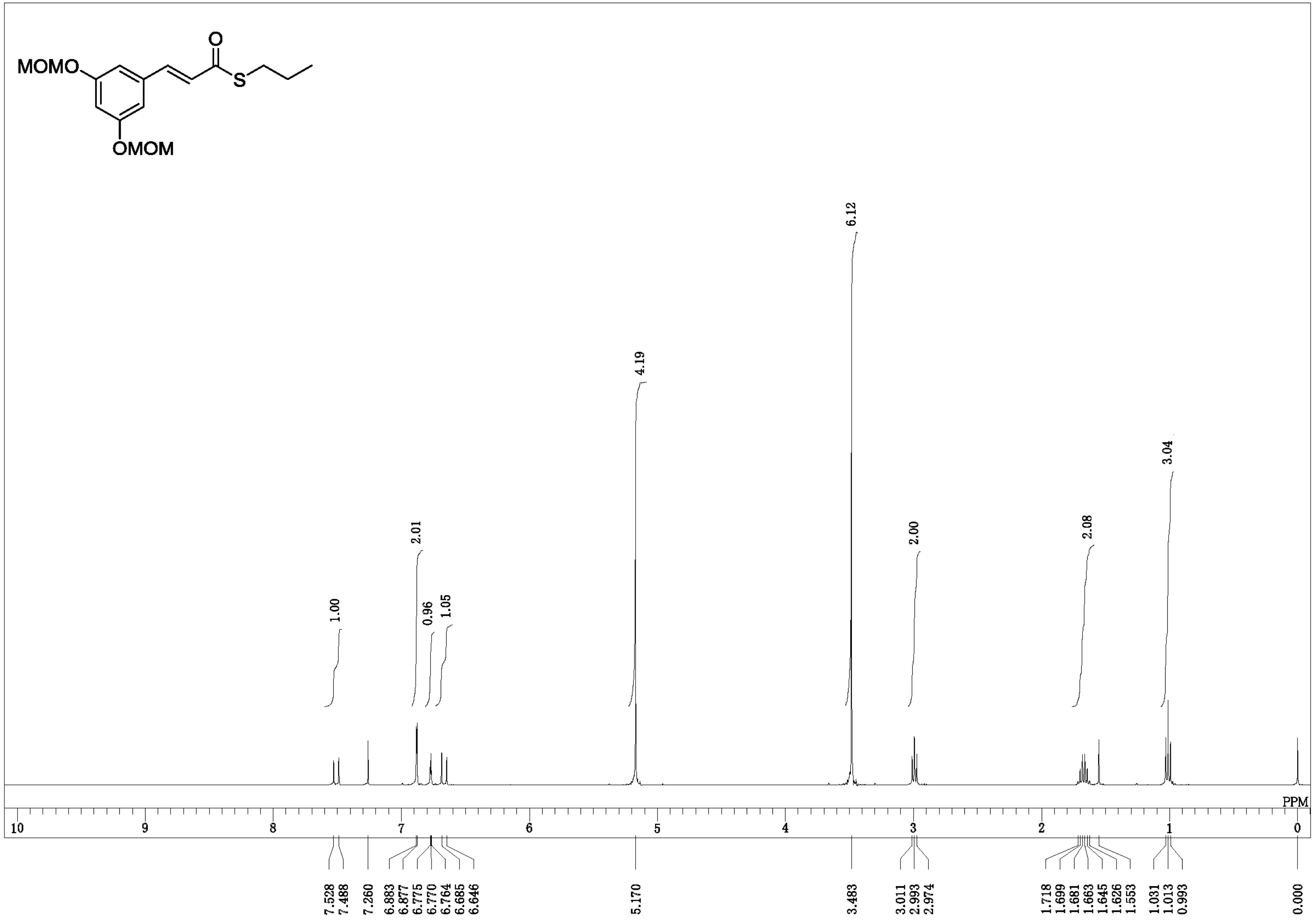

Figure S20. ^{1}H-NMR spectra of GO-Y150.

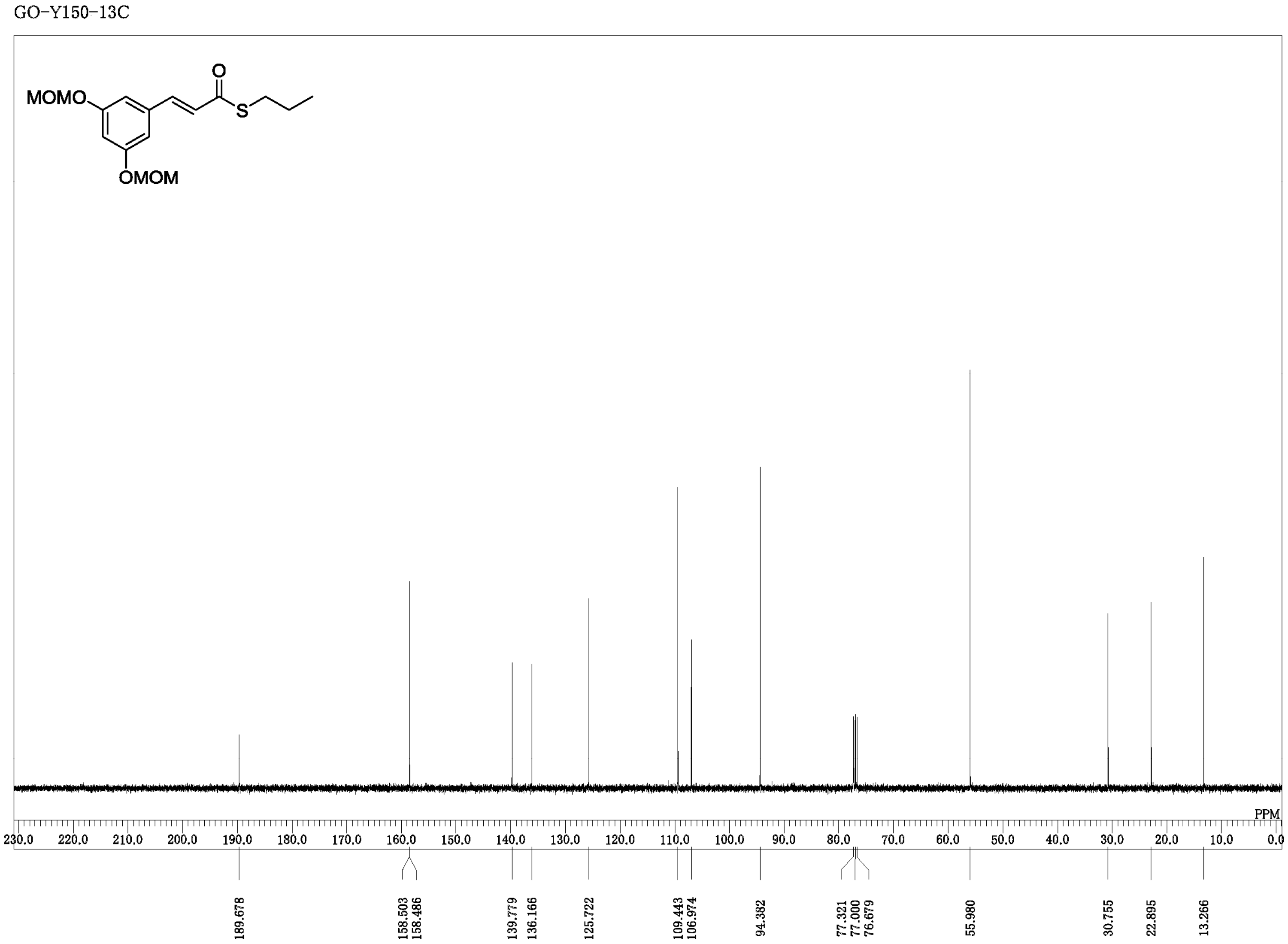

Figure S21. ^{13}C-NMR spectra of GO-Y150.

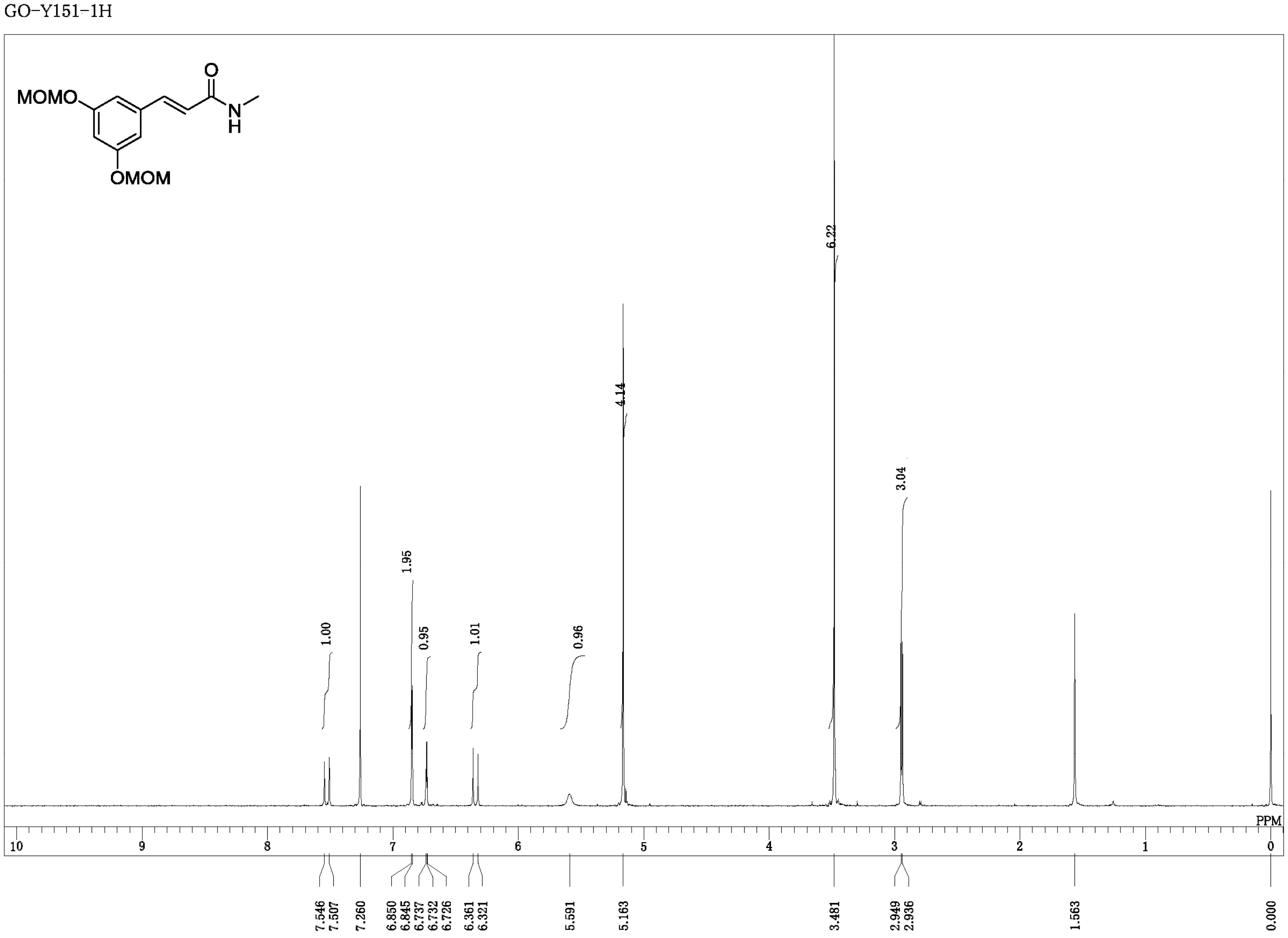

Figure S22. ^{1}H-NMR spectra of GO-Y151.

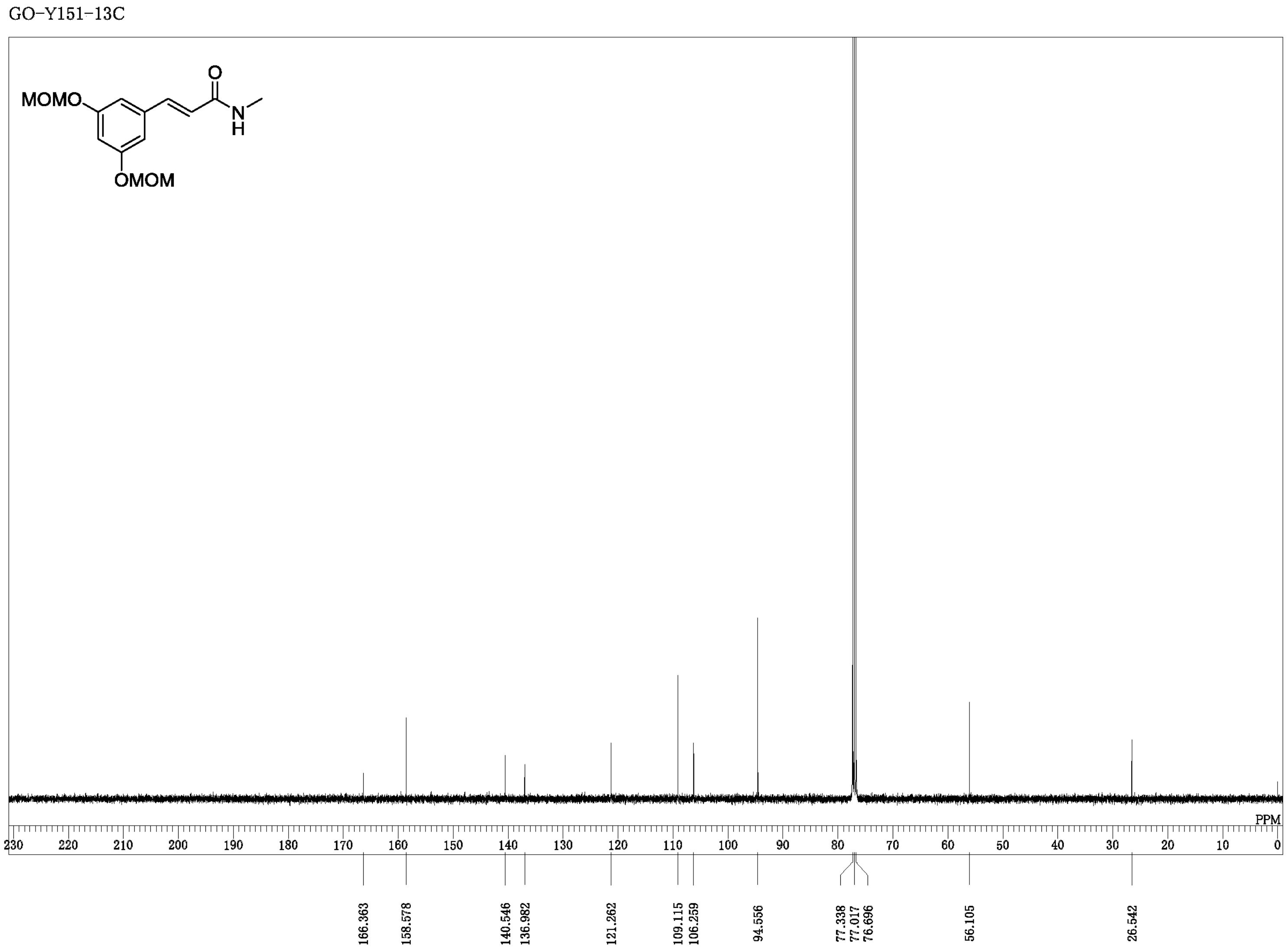

Figure S23. ^{13}C-NMR spectra of GO-Y151.

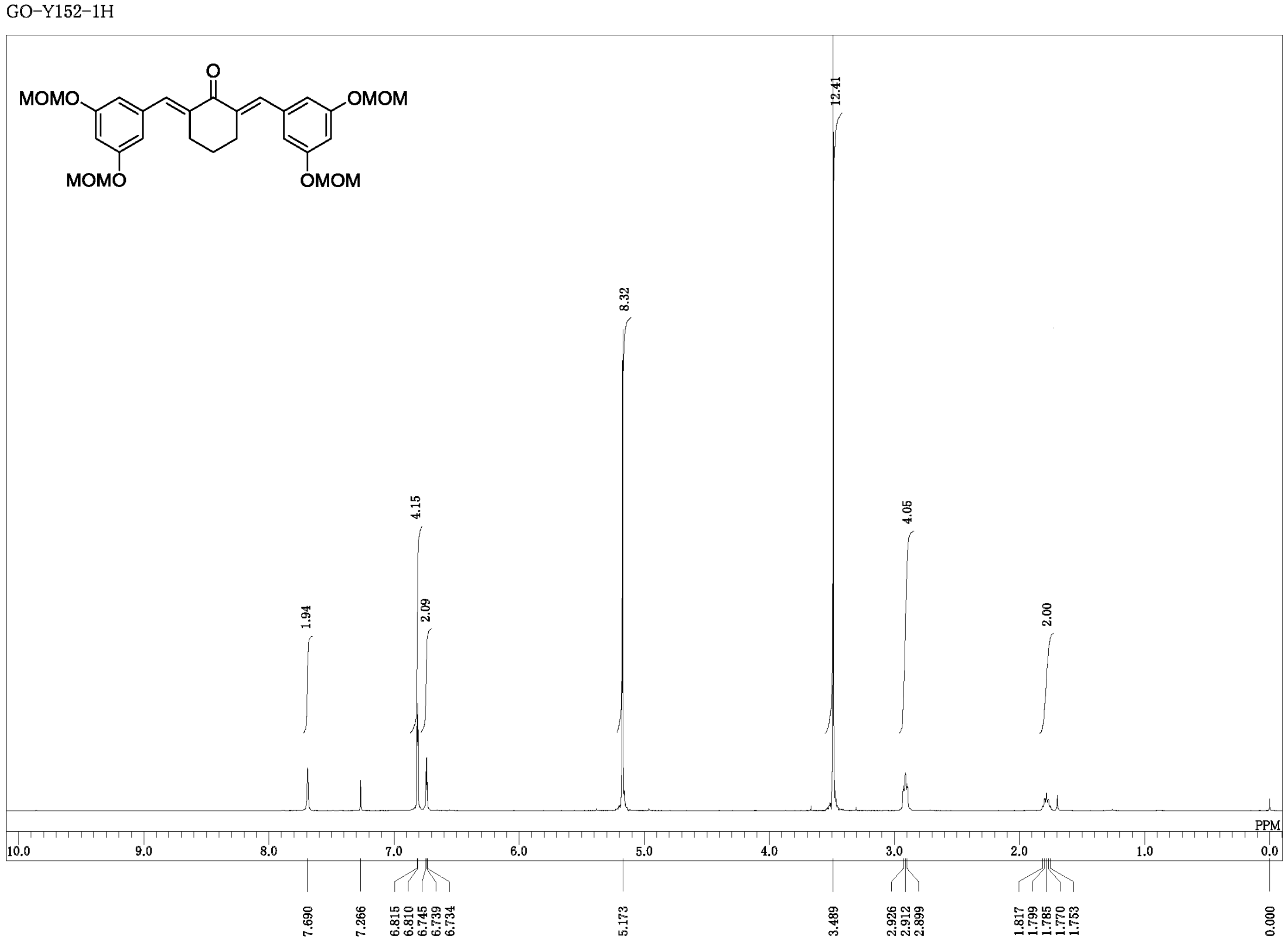

Figure S24. ^{1}H-NMR spectra of GO-Y152.

GO-Y152-13C

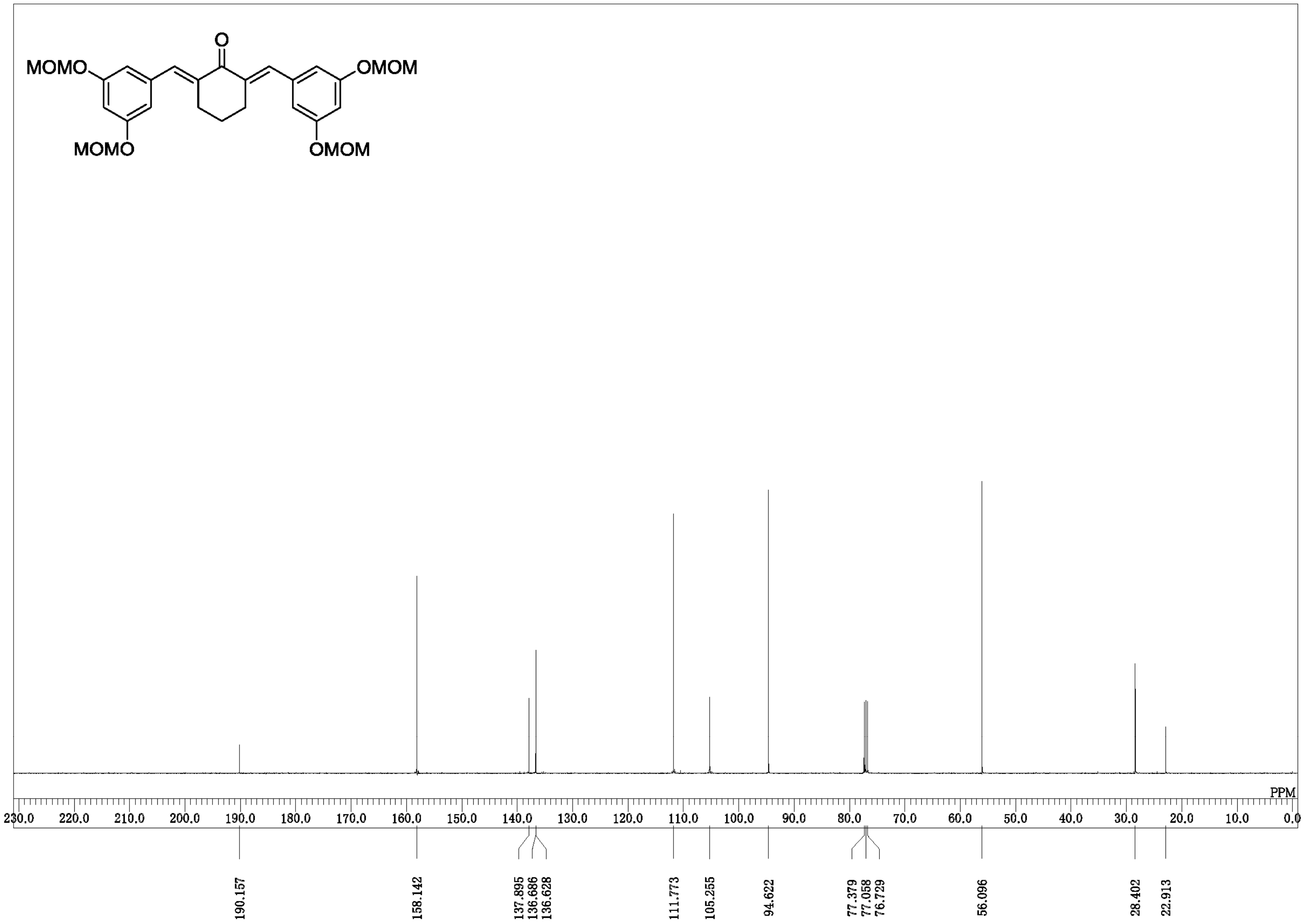

Figure S25. ^{13}C-NMR spectra of GO-Y152.

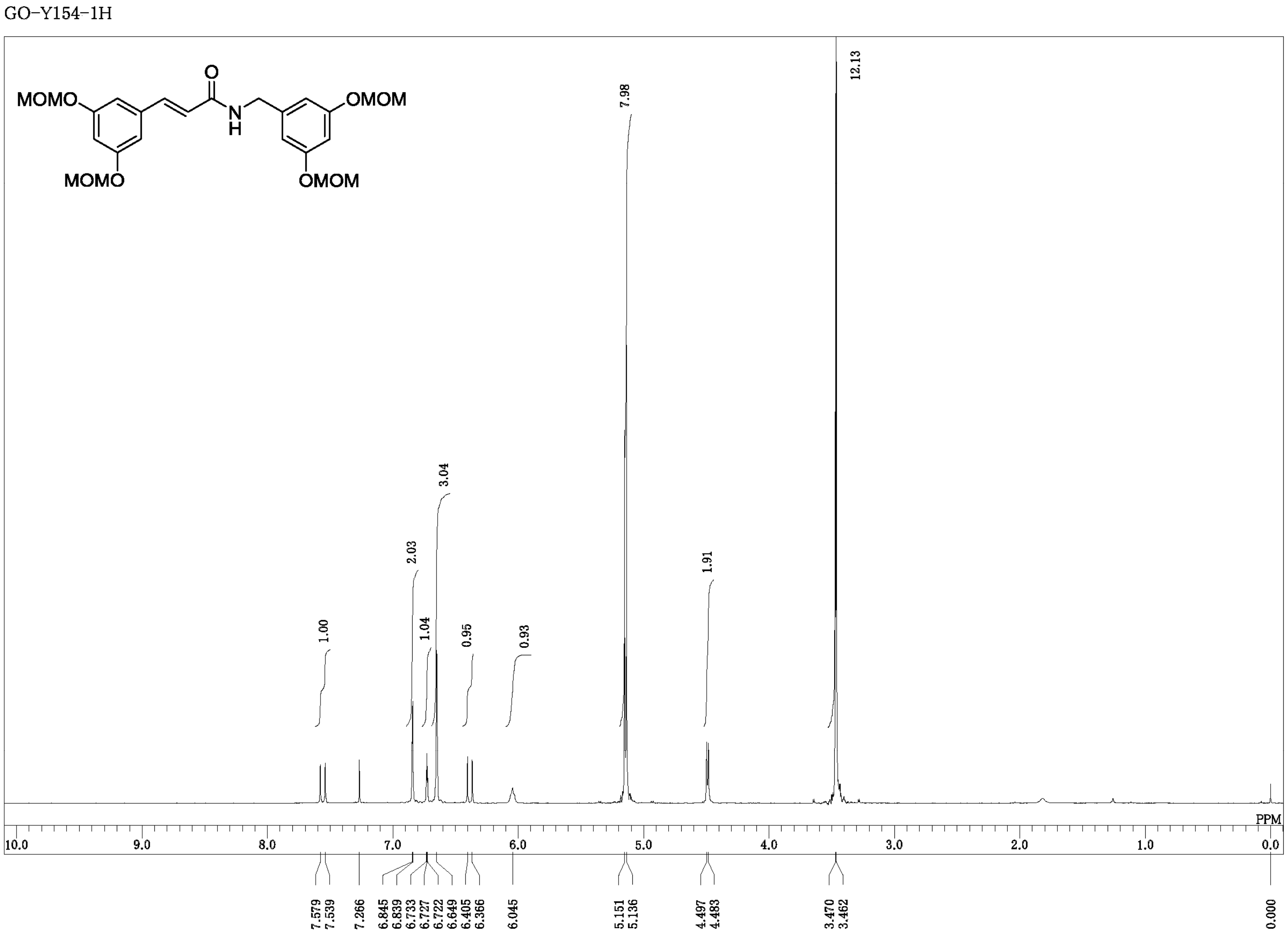

Figure S26. ^{1}H-NMR spectra of GO-Y154.

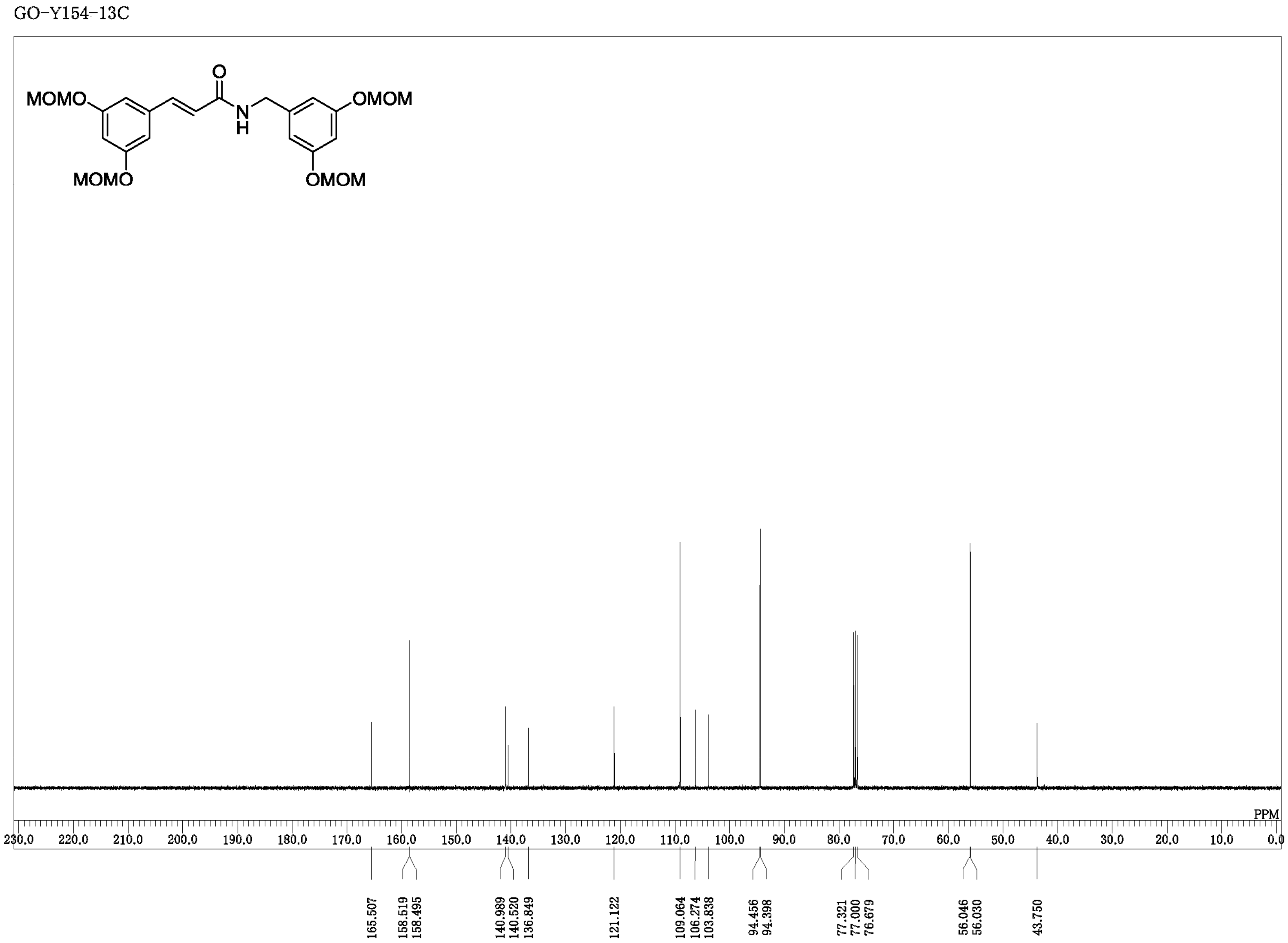

Figure S27. ^{13}C-NMR spectra of GO-Y154.

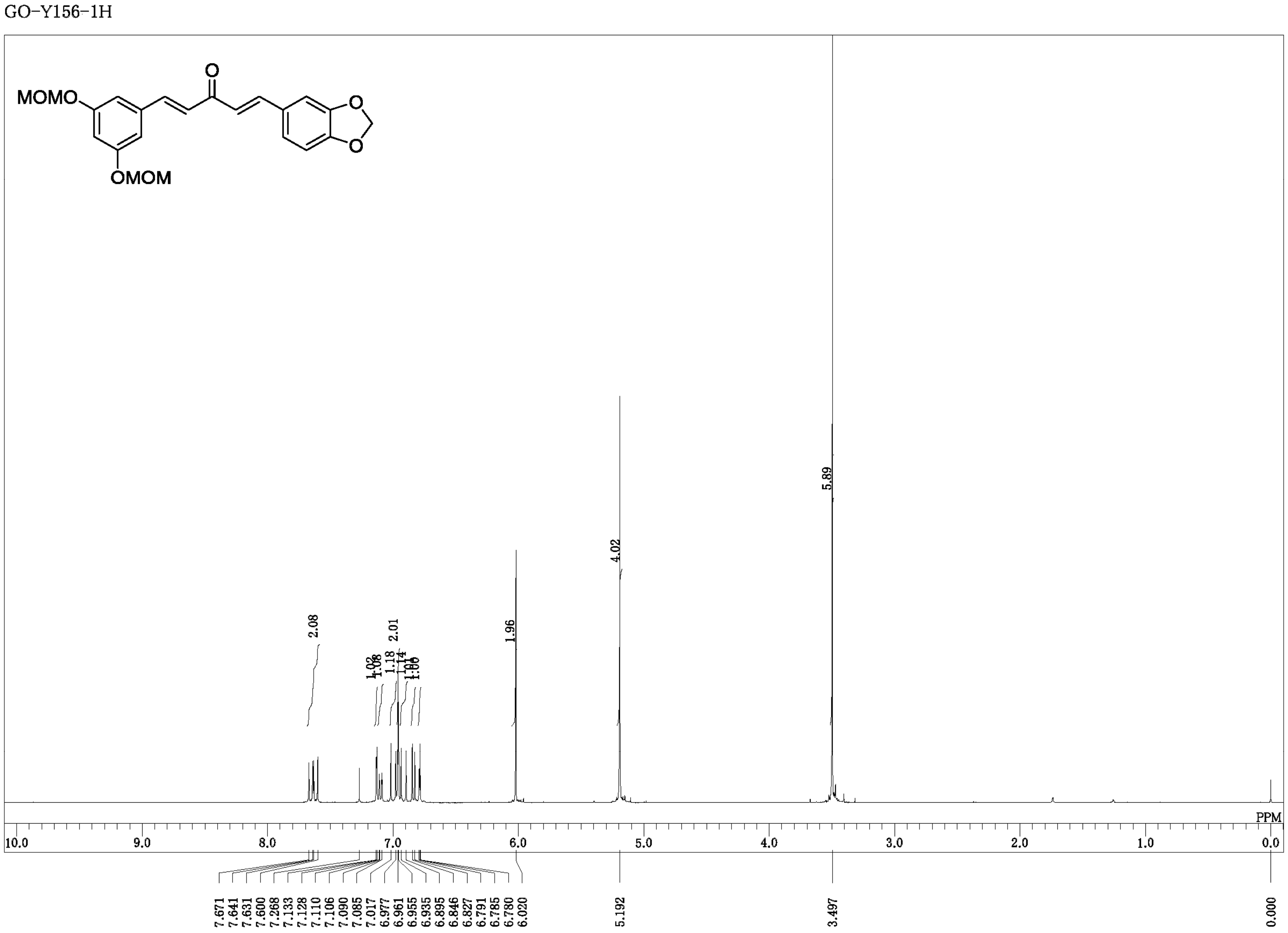

Figure S28. ^{1}H-NMR spectra of GO-Y156.

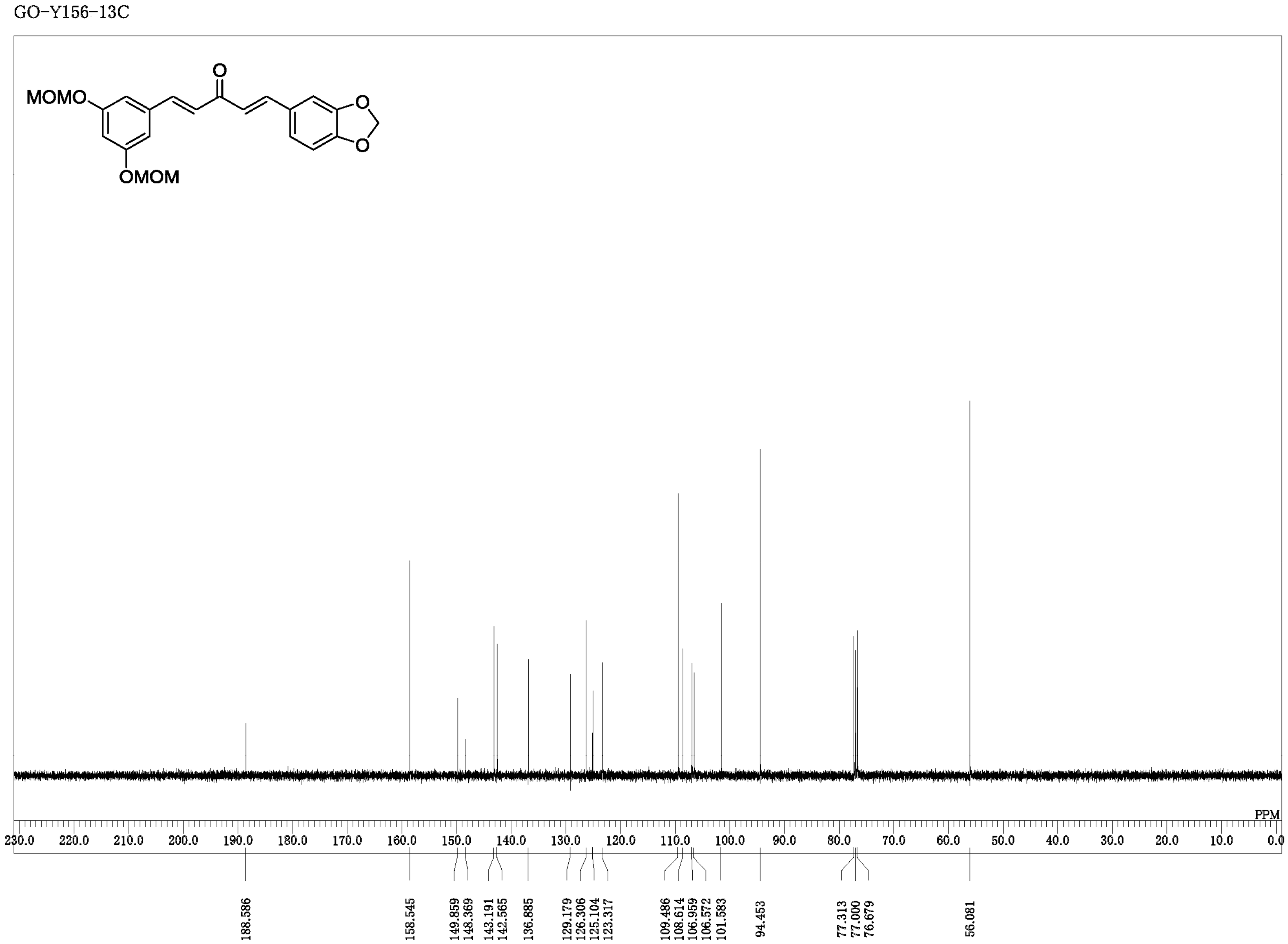

Figure S29. ^{13}C-NMR spectra of GO-Y156.

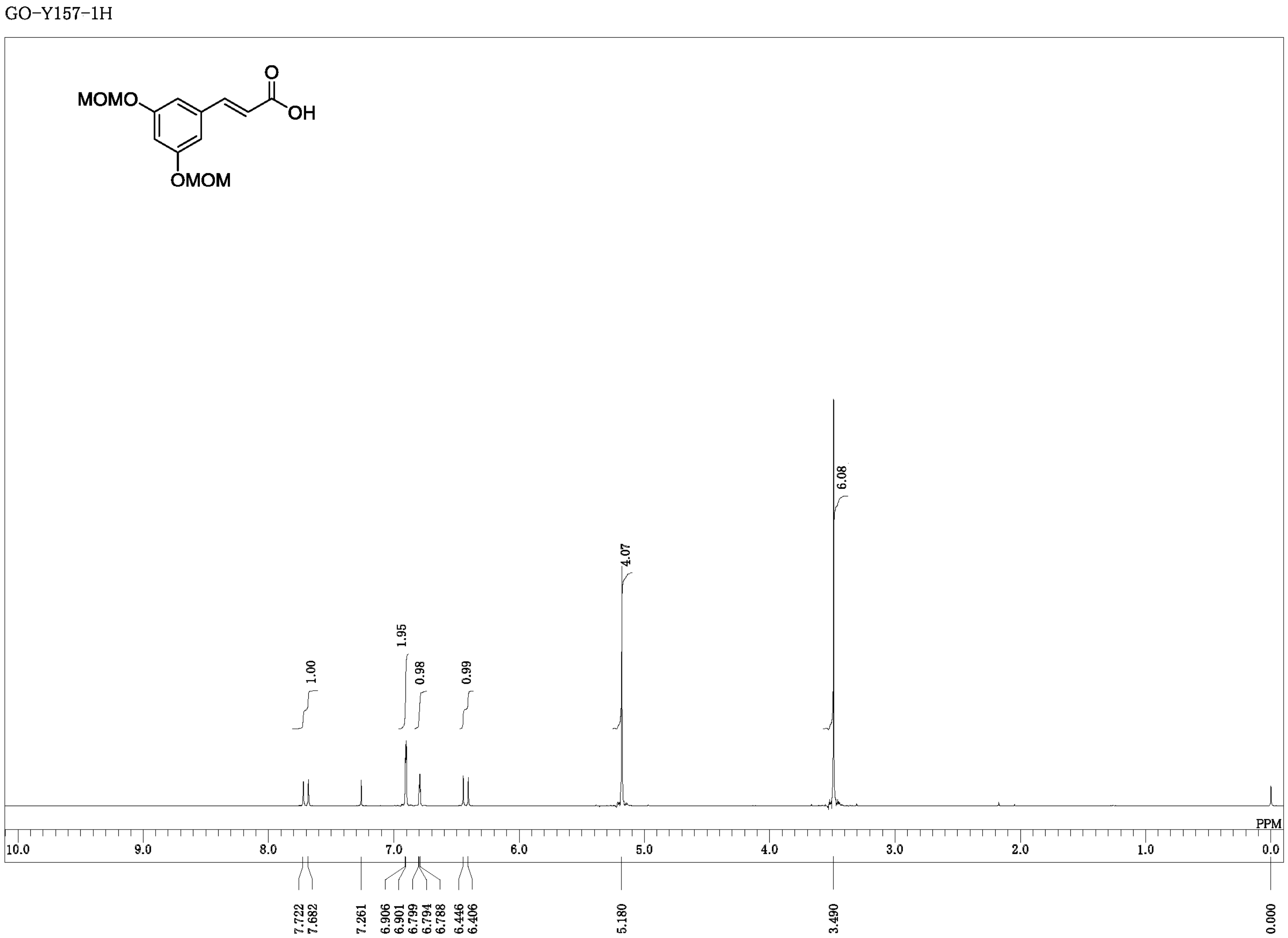

Figure S30. ^{1}H-NMR spectra of GO-Y157.

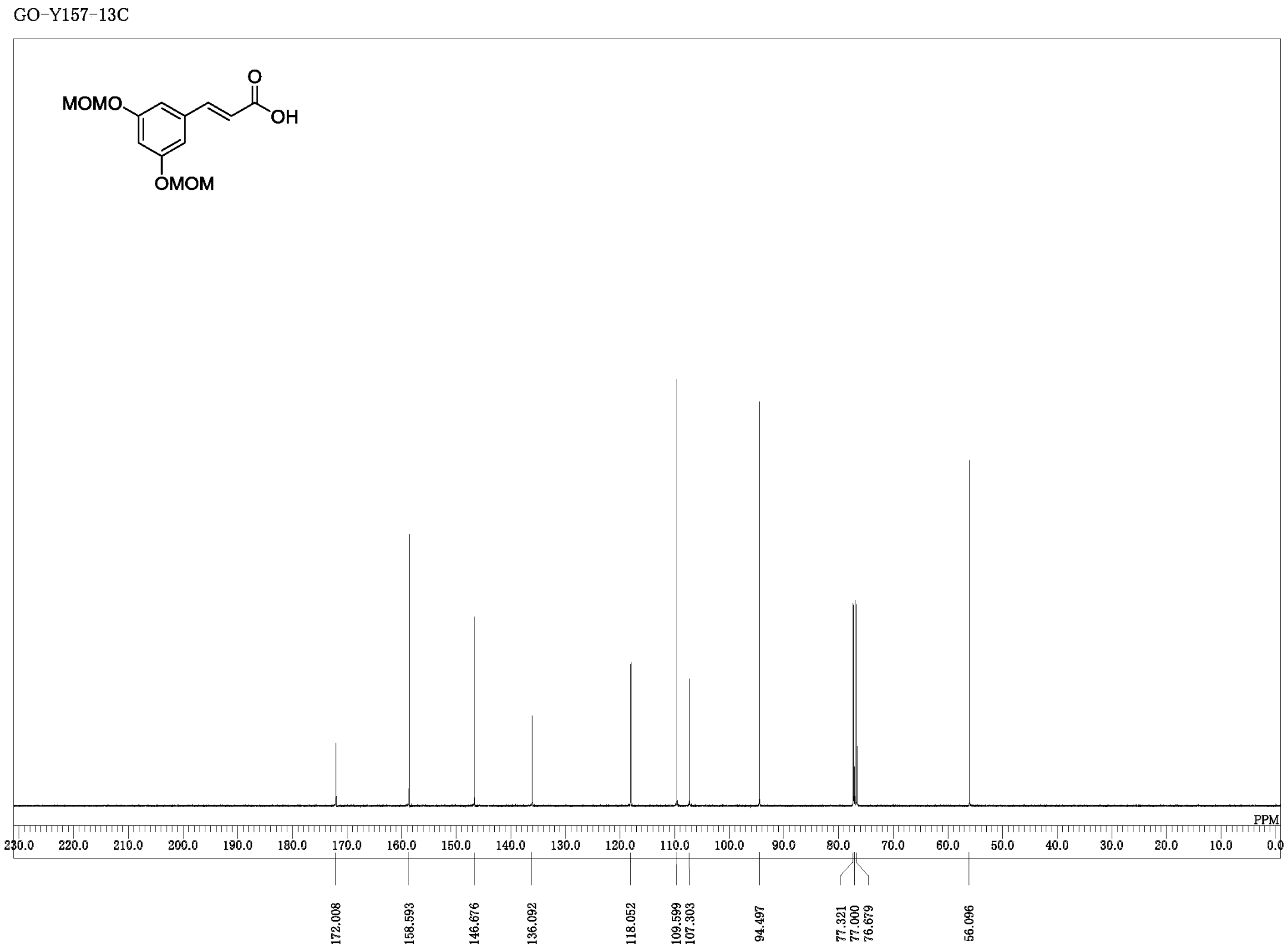

Figure S31. ^{13}C-NMR spectra of GO-Y157.

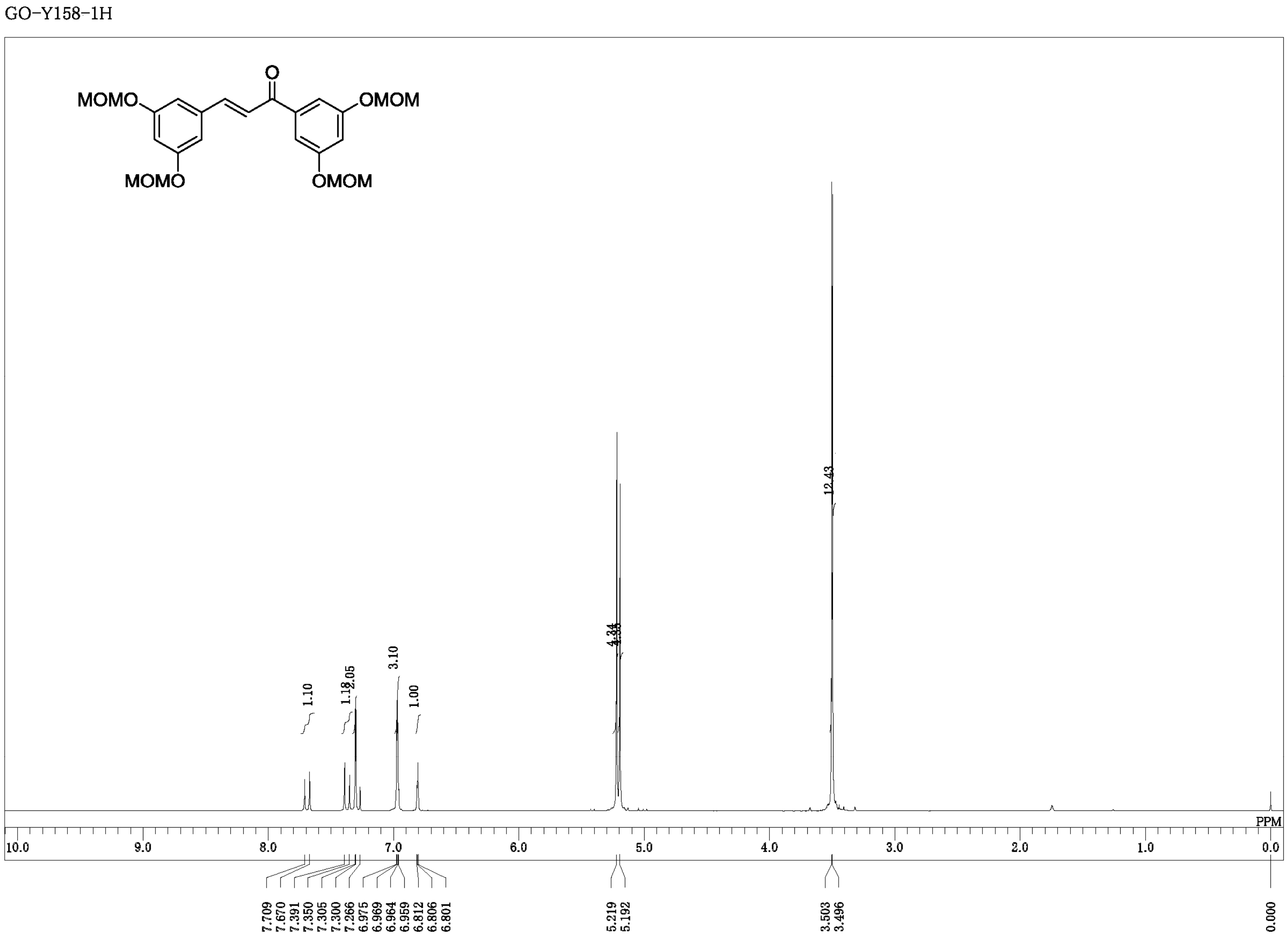

Figure S32. ^{1}H-NMR spectra of GO-Y158.

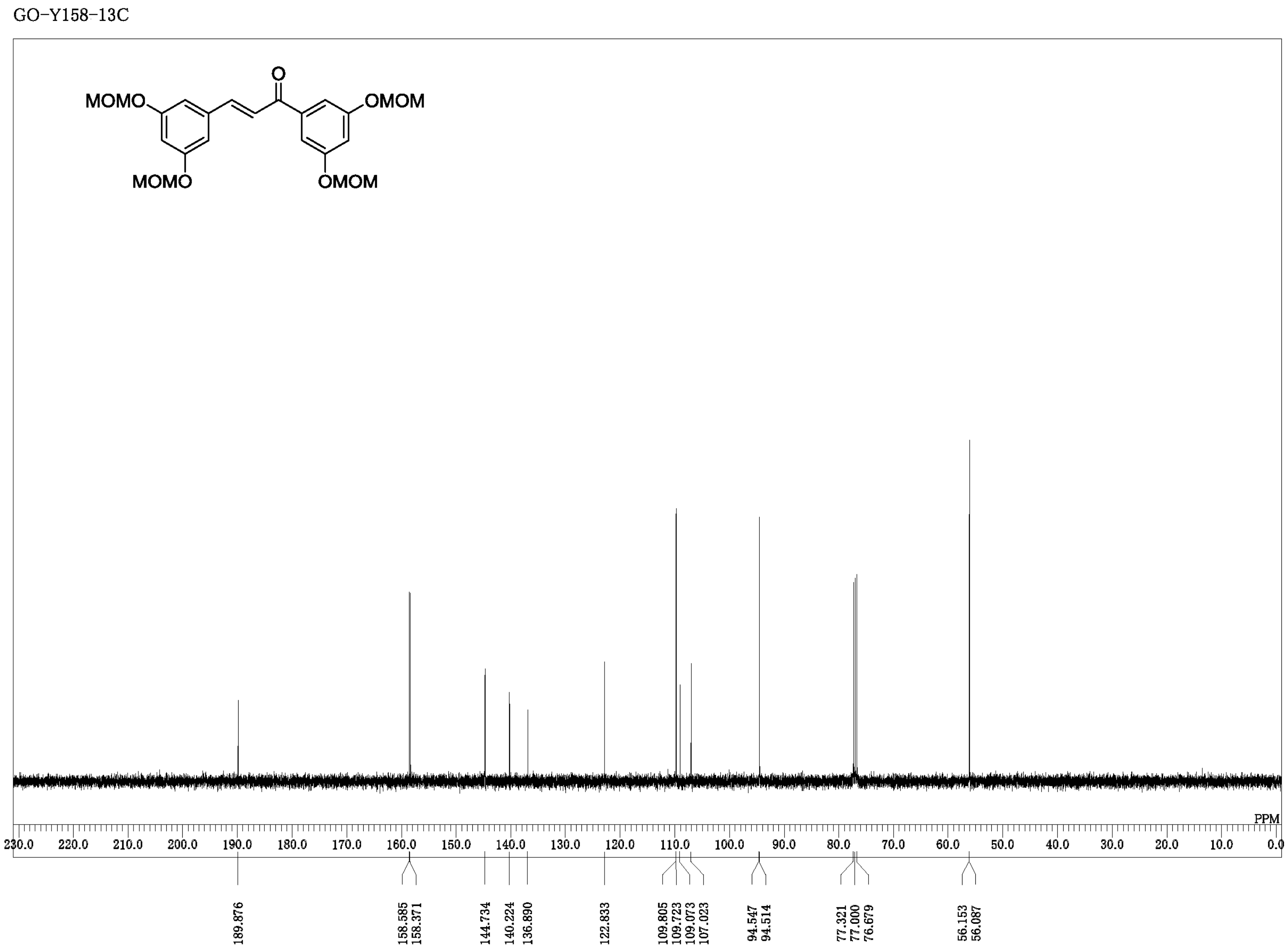

Figure S33. ^{13}C-NMR spectra of GO-Y158.

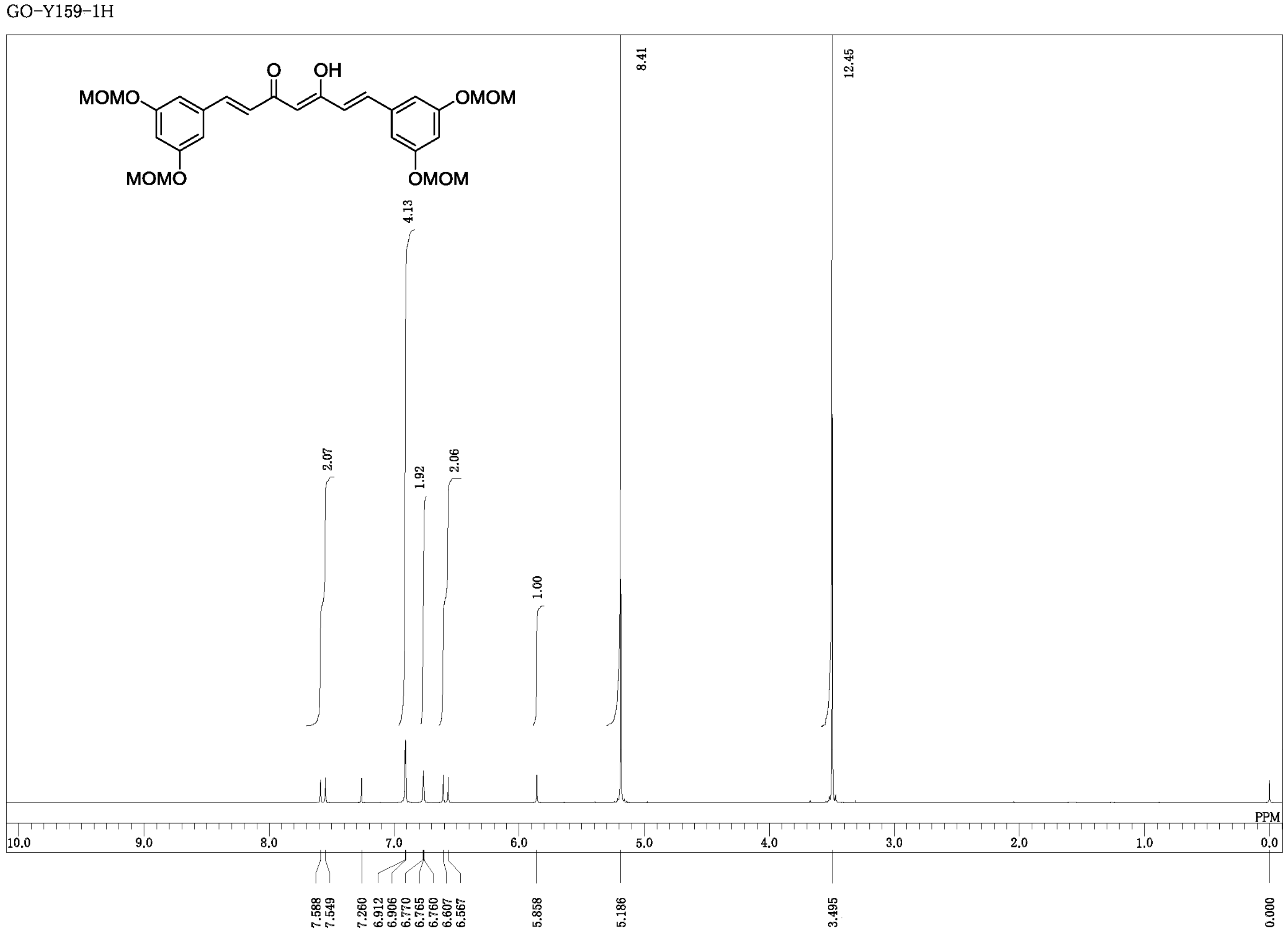

Figure S34. ^{1}H-NMR spectra of GO-Y159.

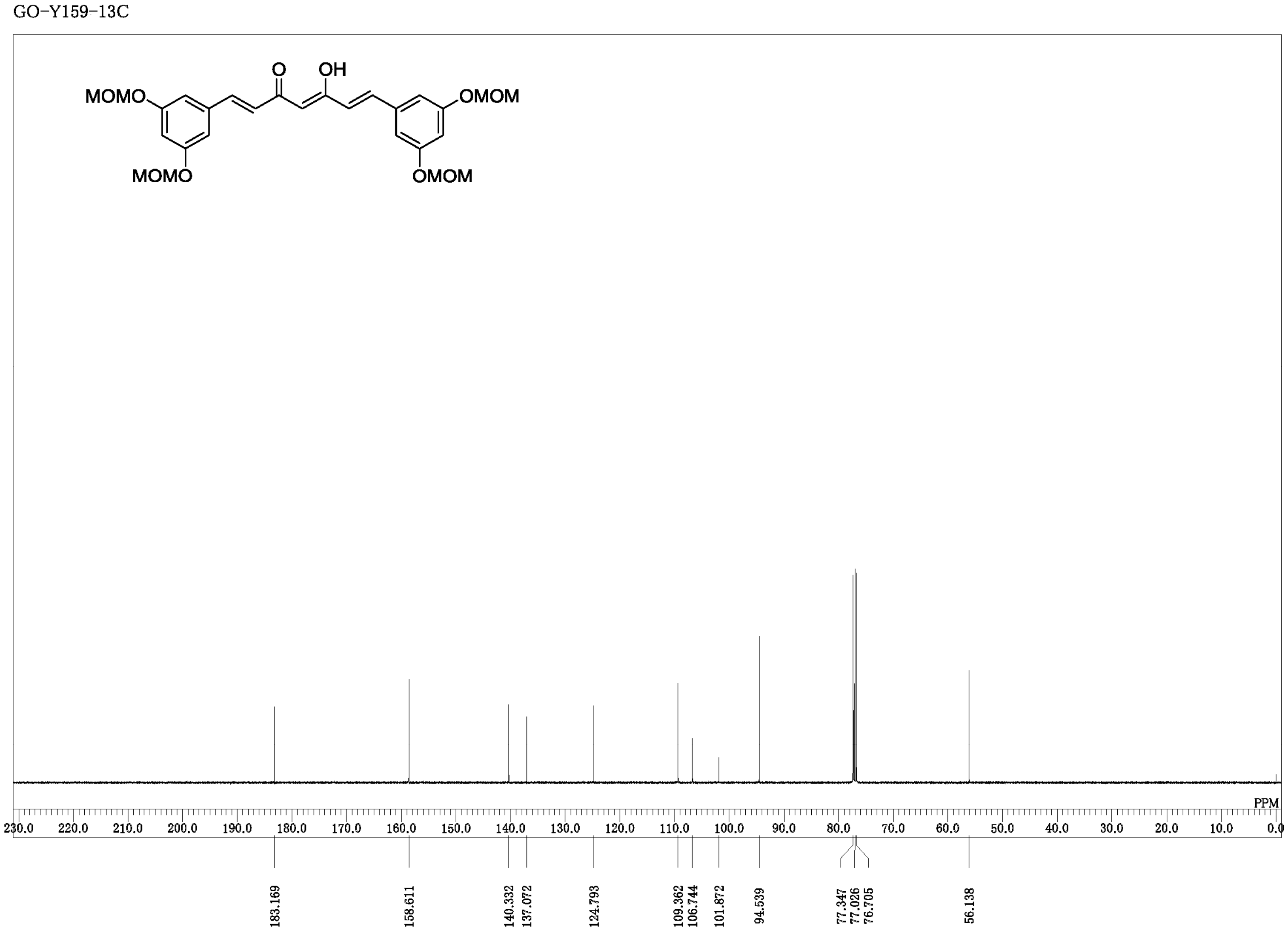

Figure S35. ^{13}C-NMR spectra of GO-Y159.

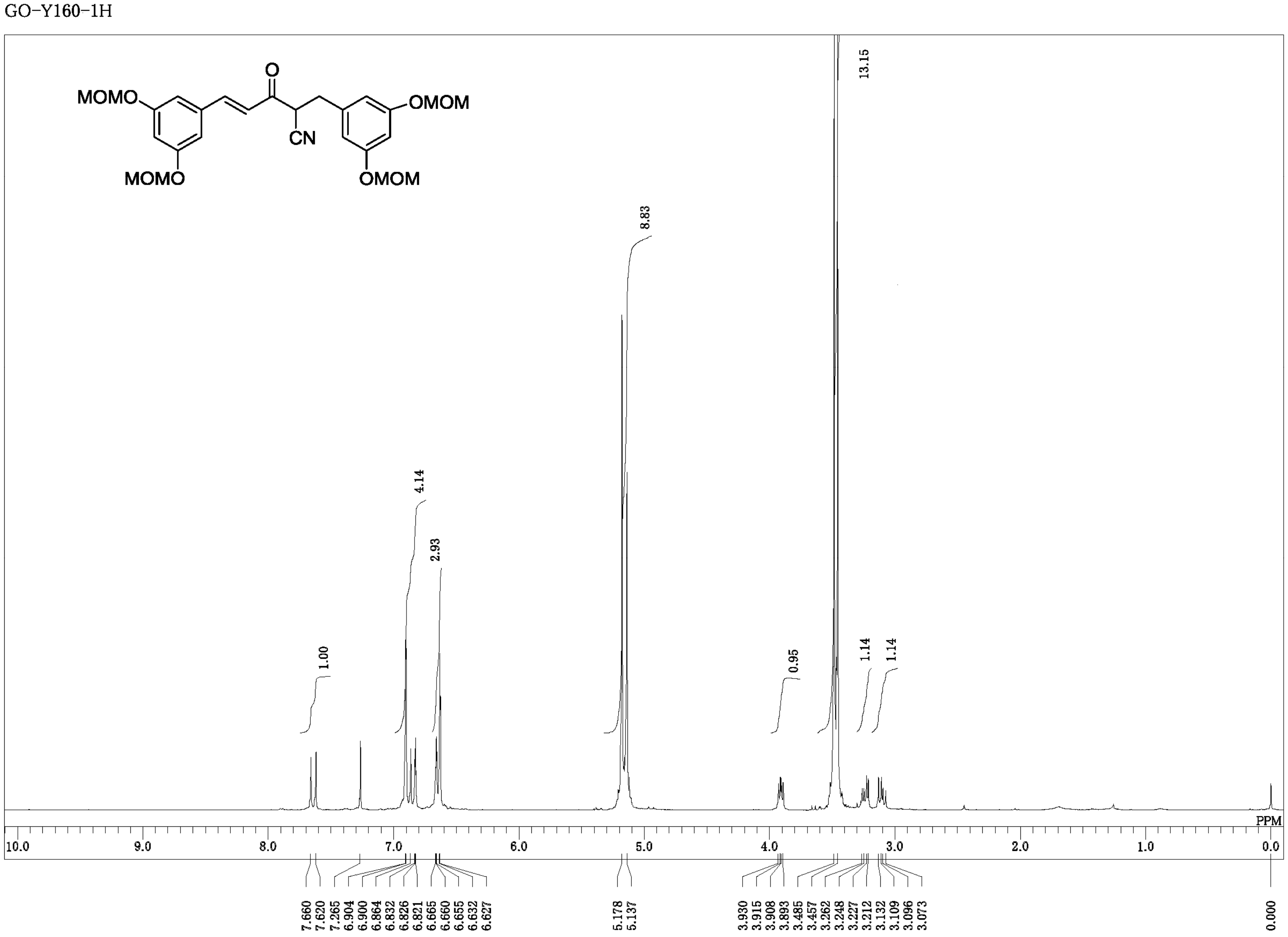

Figure S36. ^{1}H-NMR spectra of GO-Y160.

GO-Y160-13C

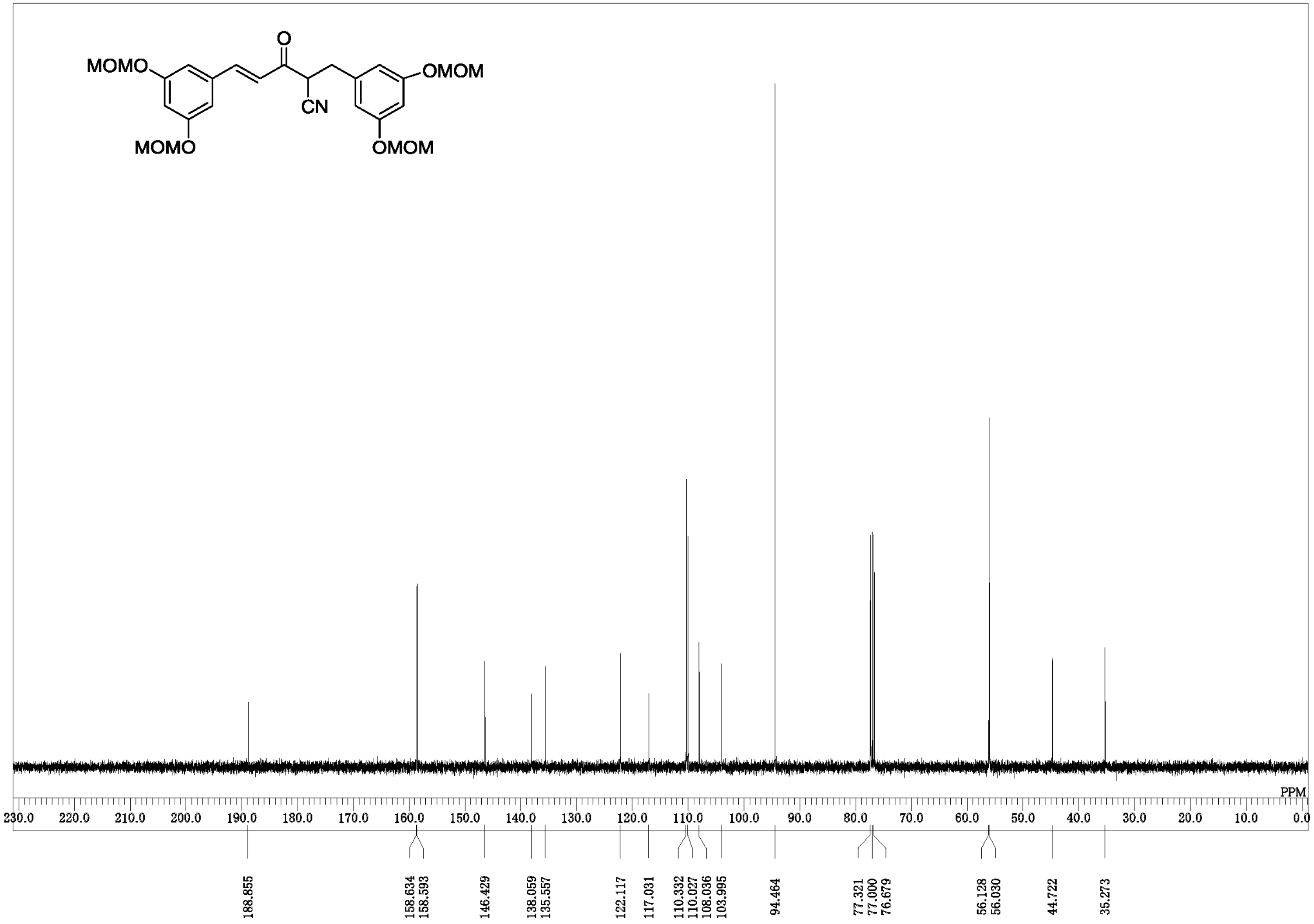

Figure S37. ^{13}C-NMR spectra of GO-Y160.

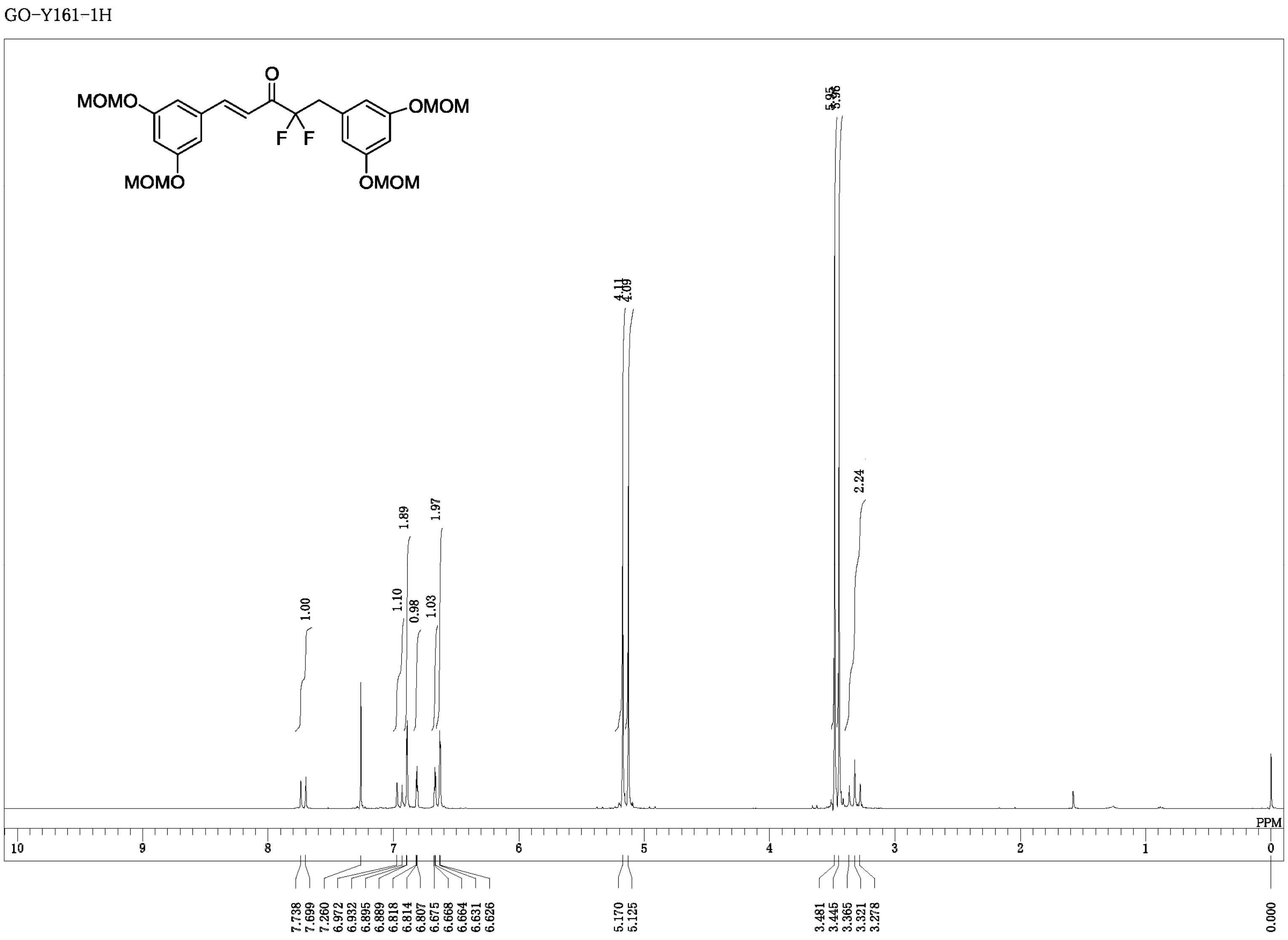

Figure S38. ^{1}H-NMR spectra of GO-Y161.

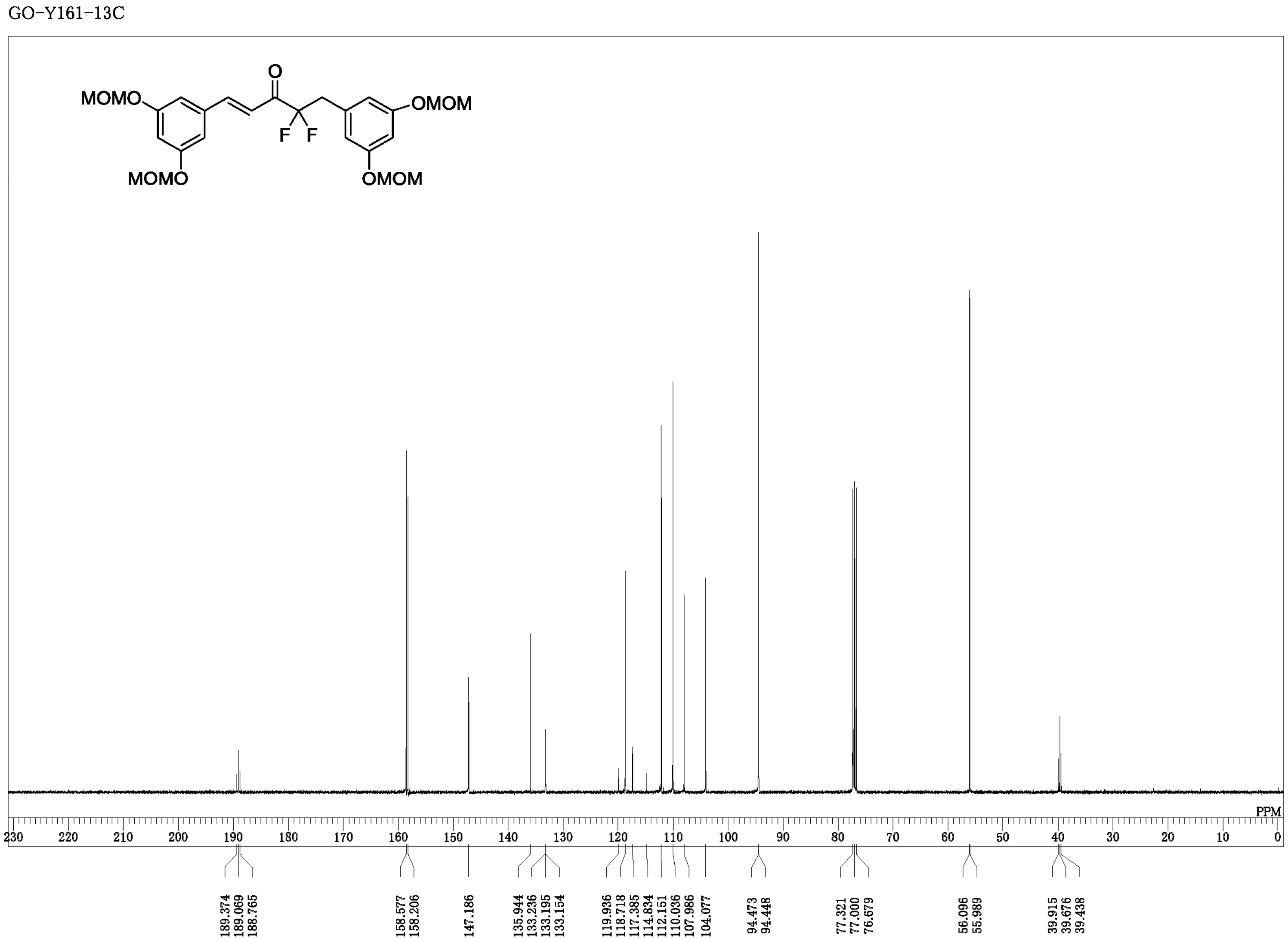

Figure S39. ^{13}C-NMR spectra of GO-Y161.

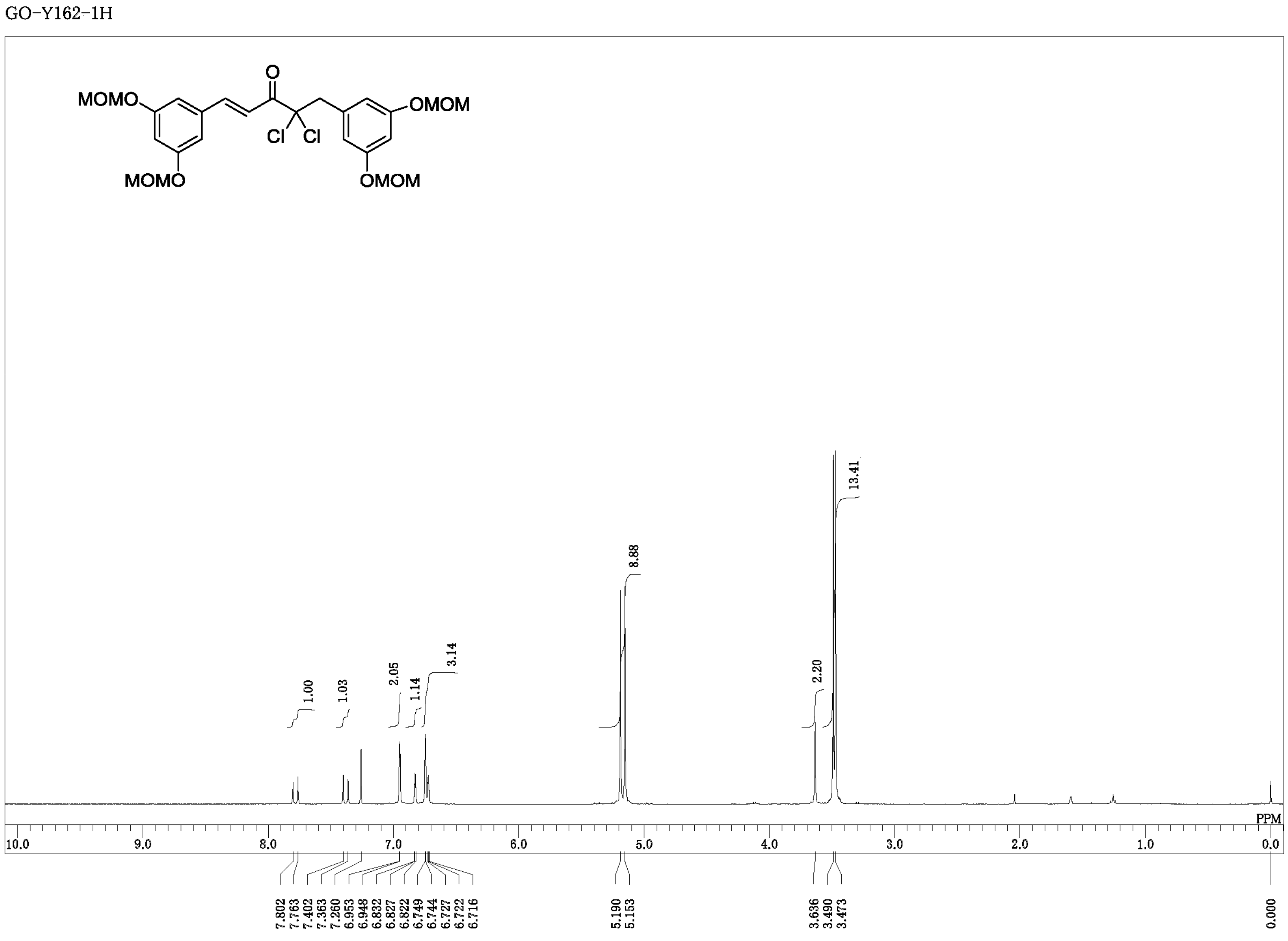

Figure S40. ^{1}H-NMR spectra of GO-Y162.

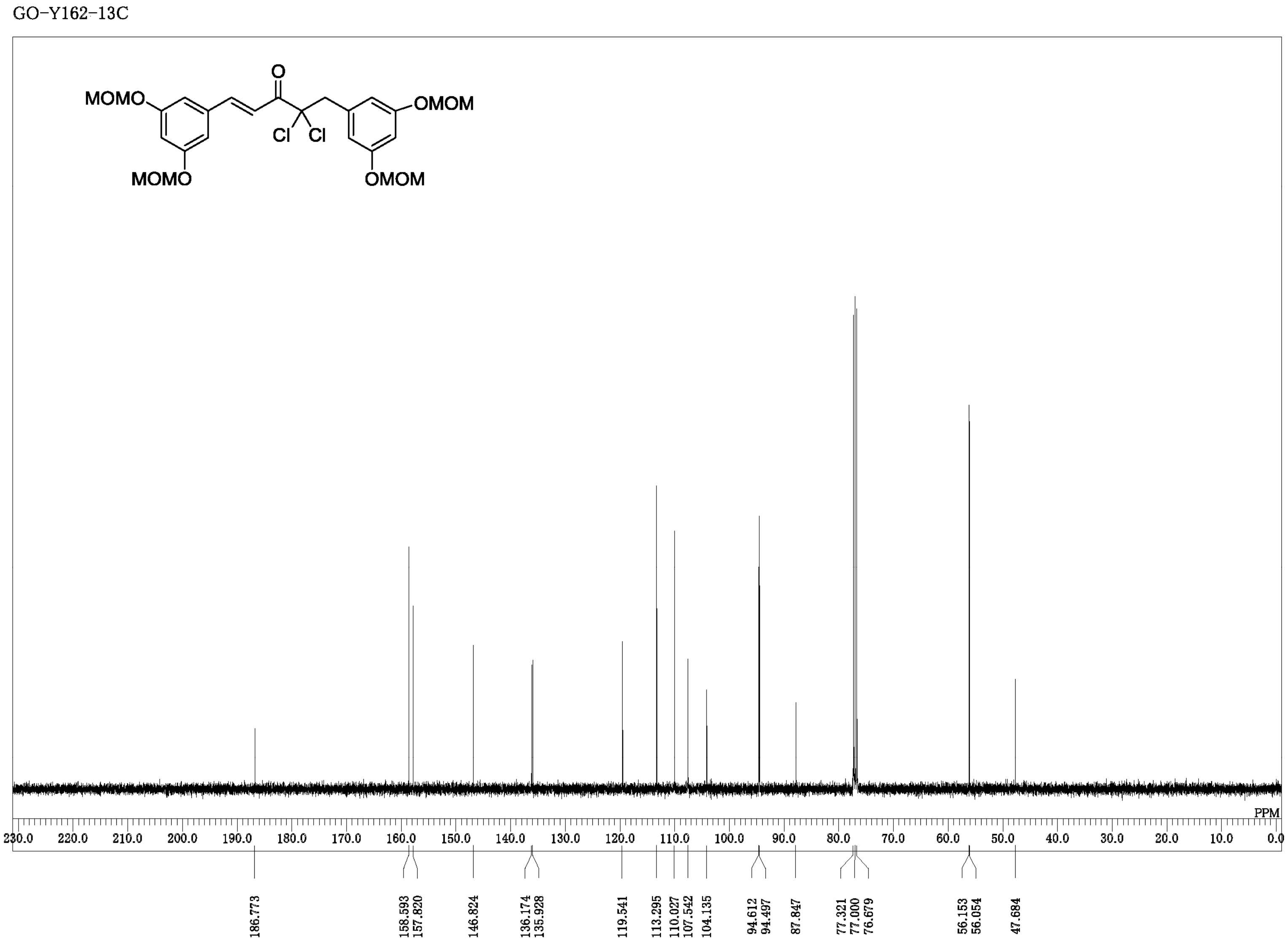

Figure S41. ^{13}C-NMR spectra of GO-Y162.

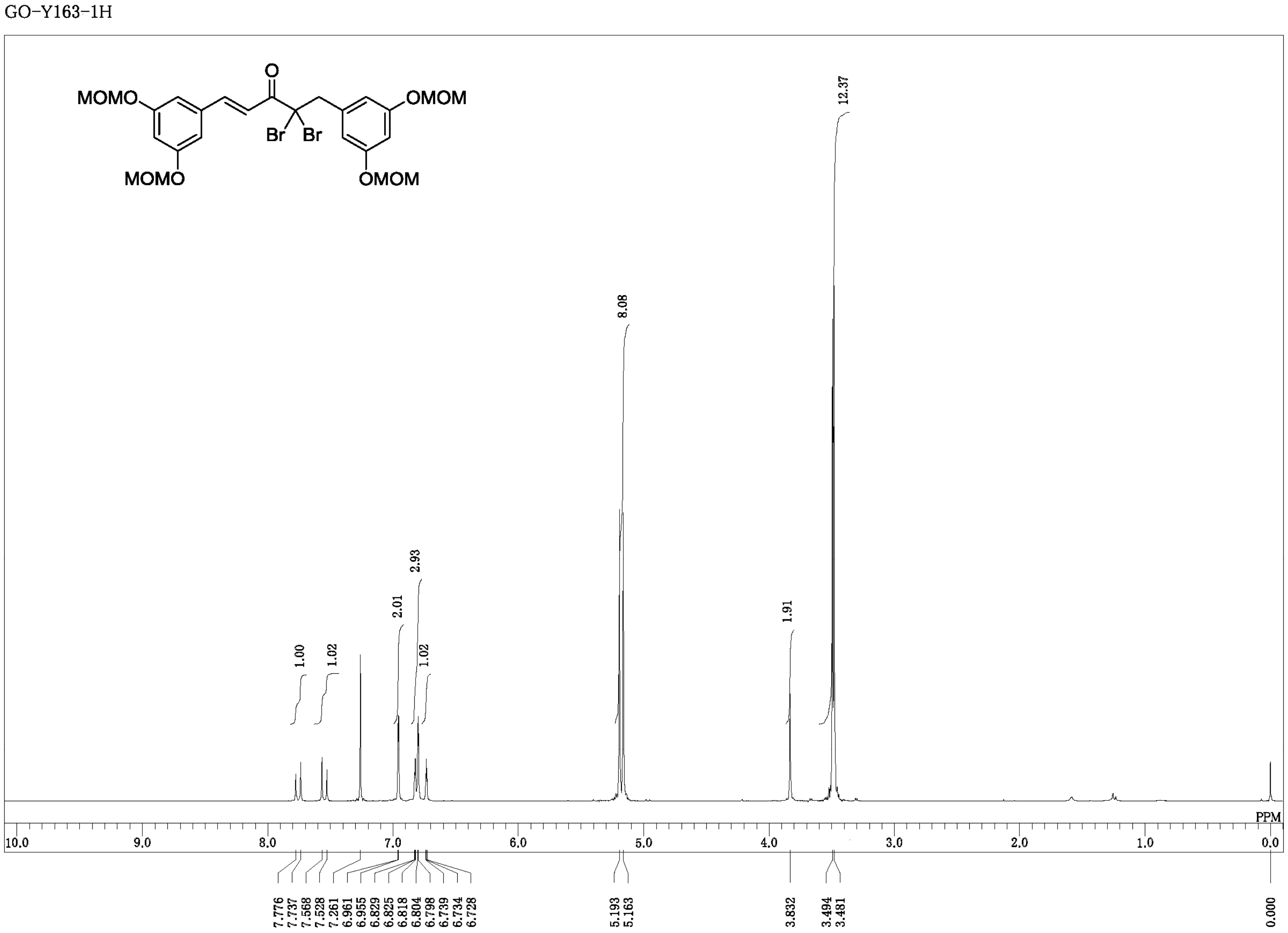

Figure S42. ^{1}H-NMR spectra of GO-Y163.

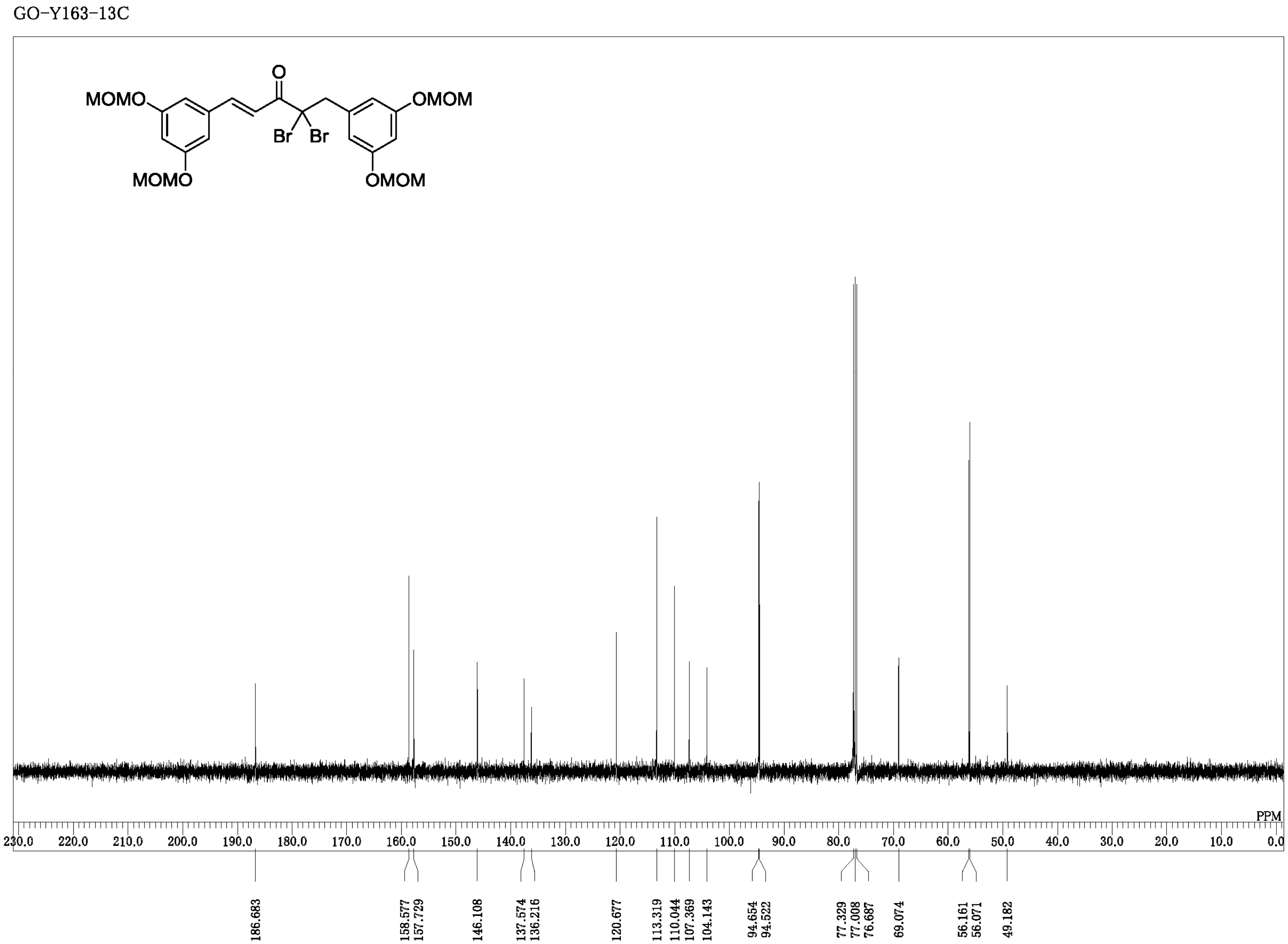

Figure S43. ^{13}C-NMR spectra of GO-Y163.

GO-Y164-1H

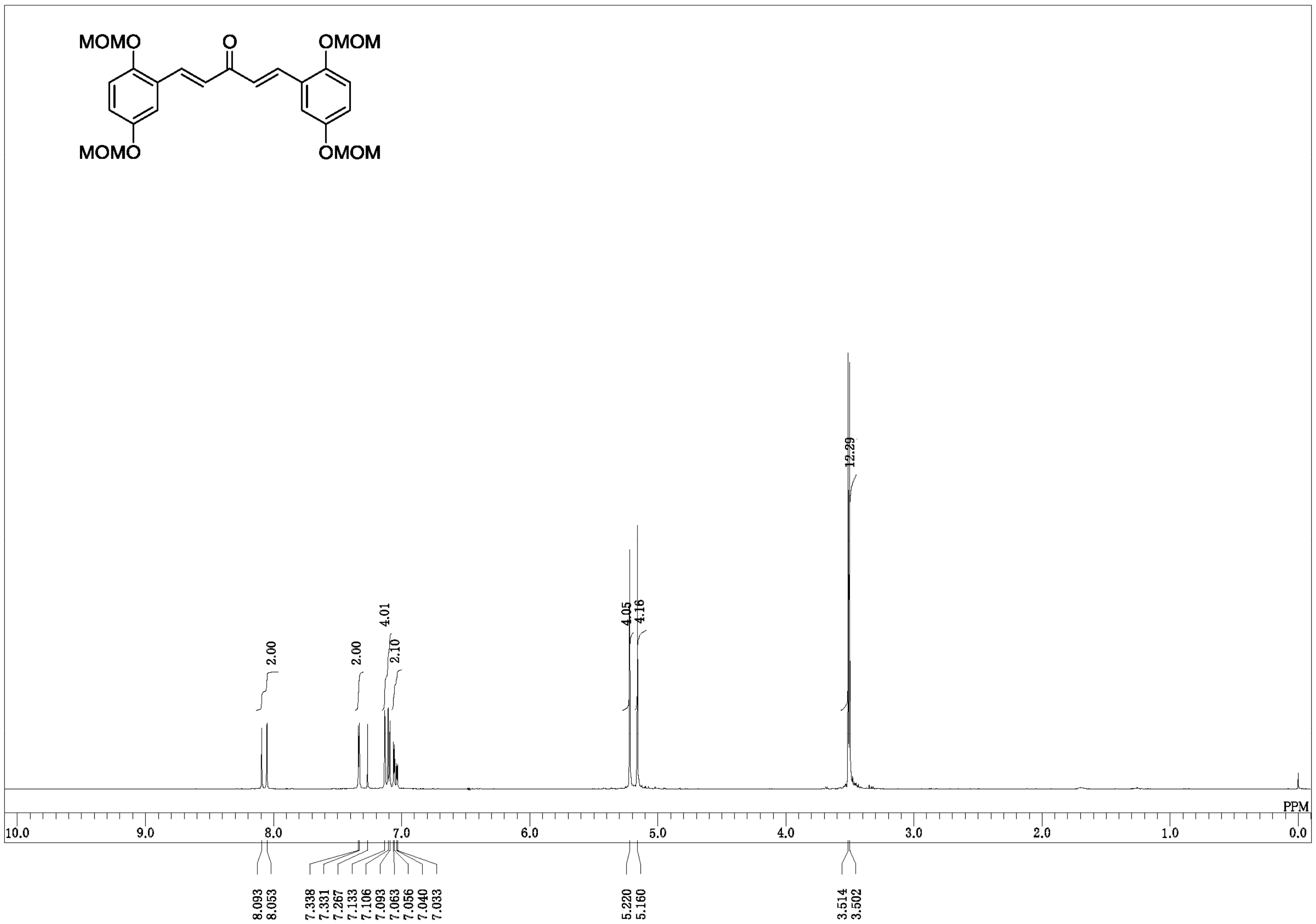

Figure S44. ^{1}H-NMR spectra of GO-Y164.

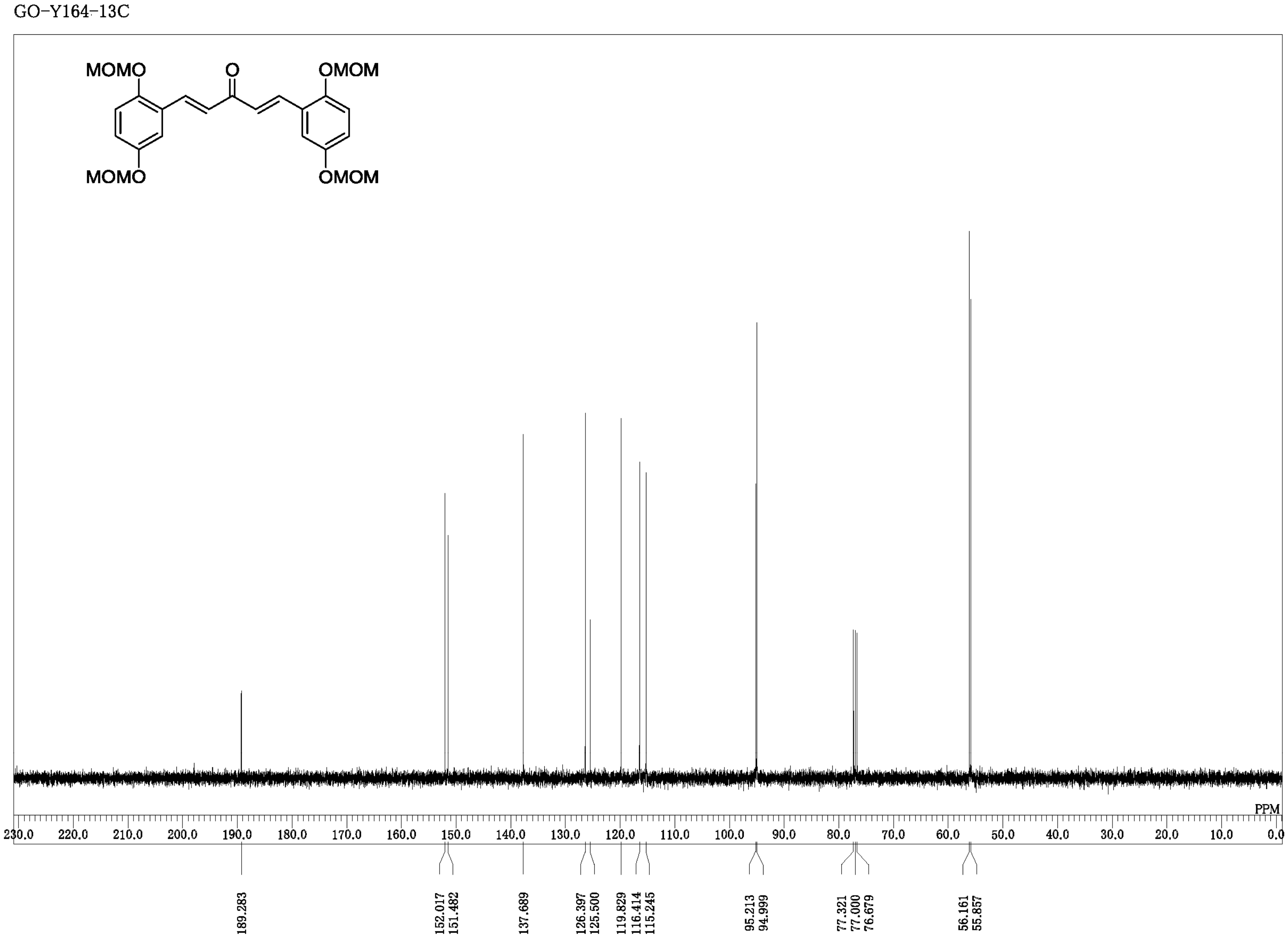

Figure S45. ^{13}C-NMR spectra of GO-Y164.

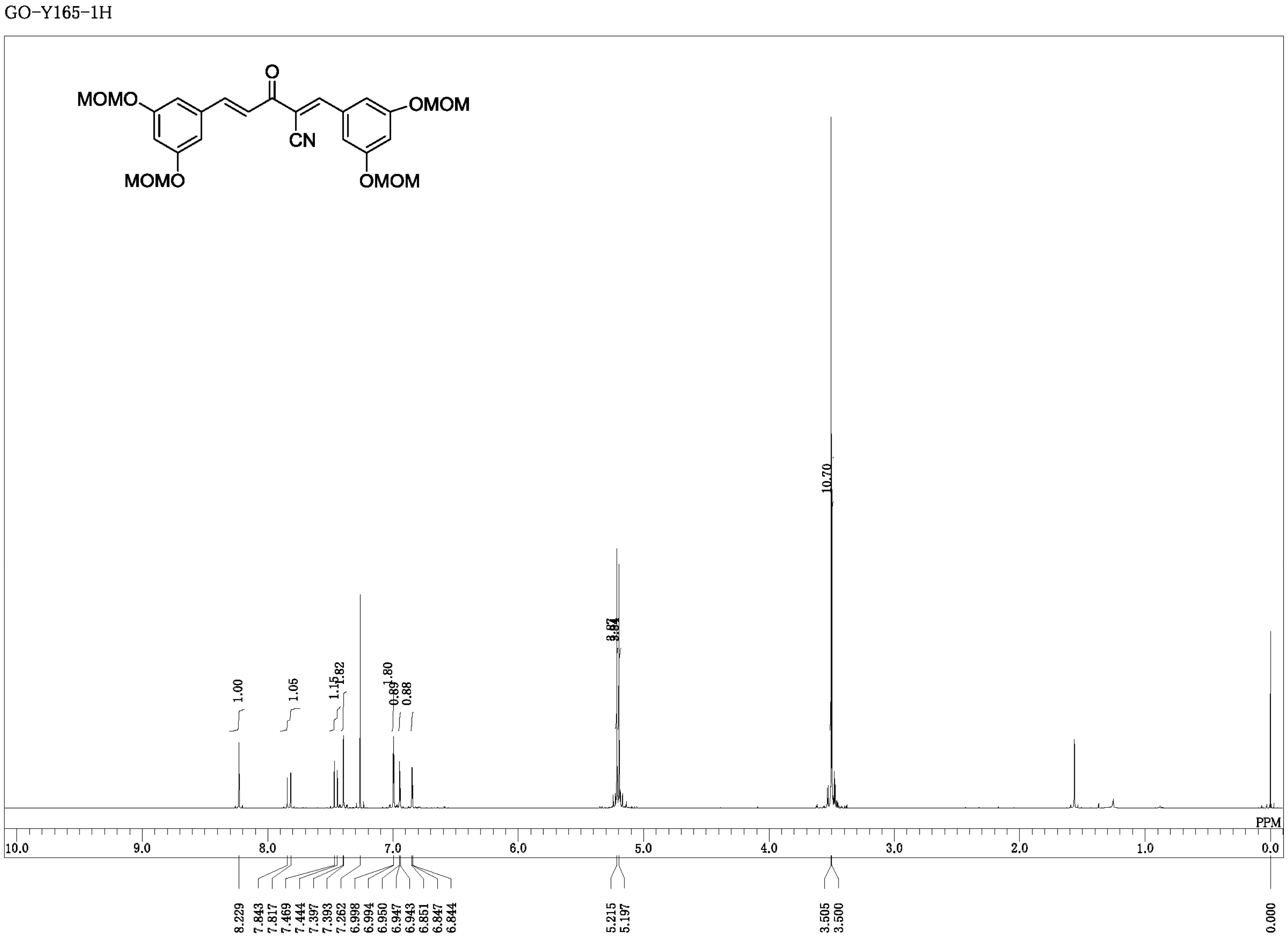

Figure S46. ^{1}H-NMR spectra of GO-Y165.

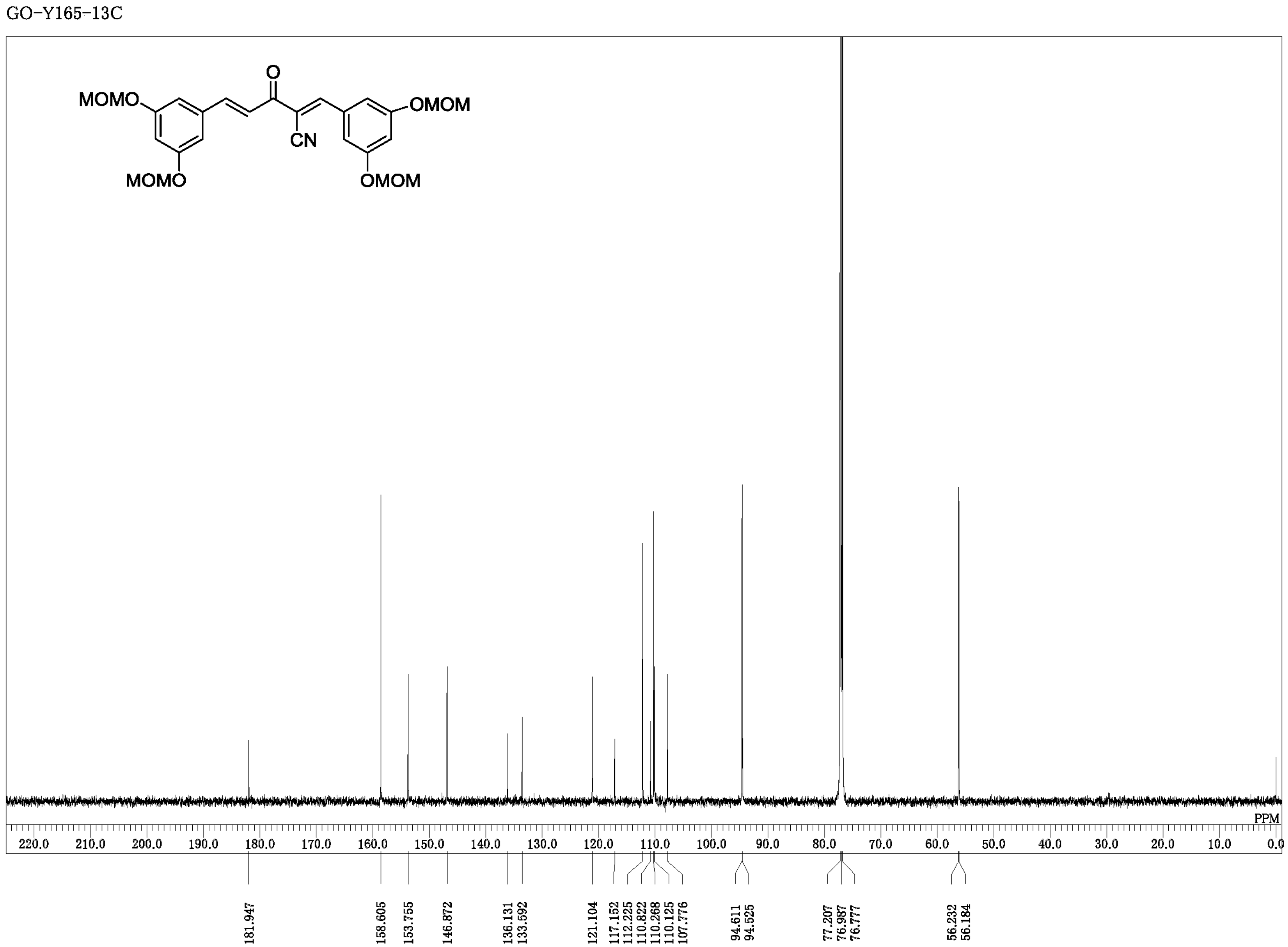

Figure S47. ^{13}C-NMR spectra of GO-Y165.

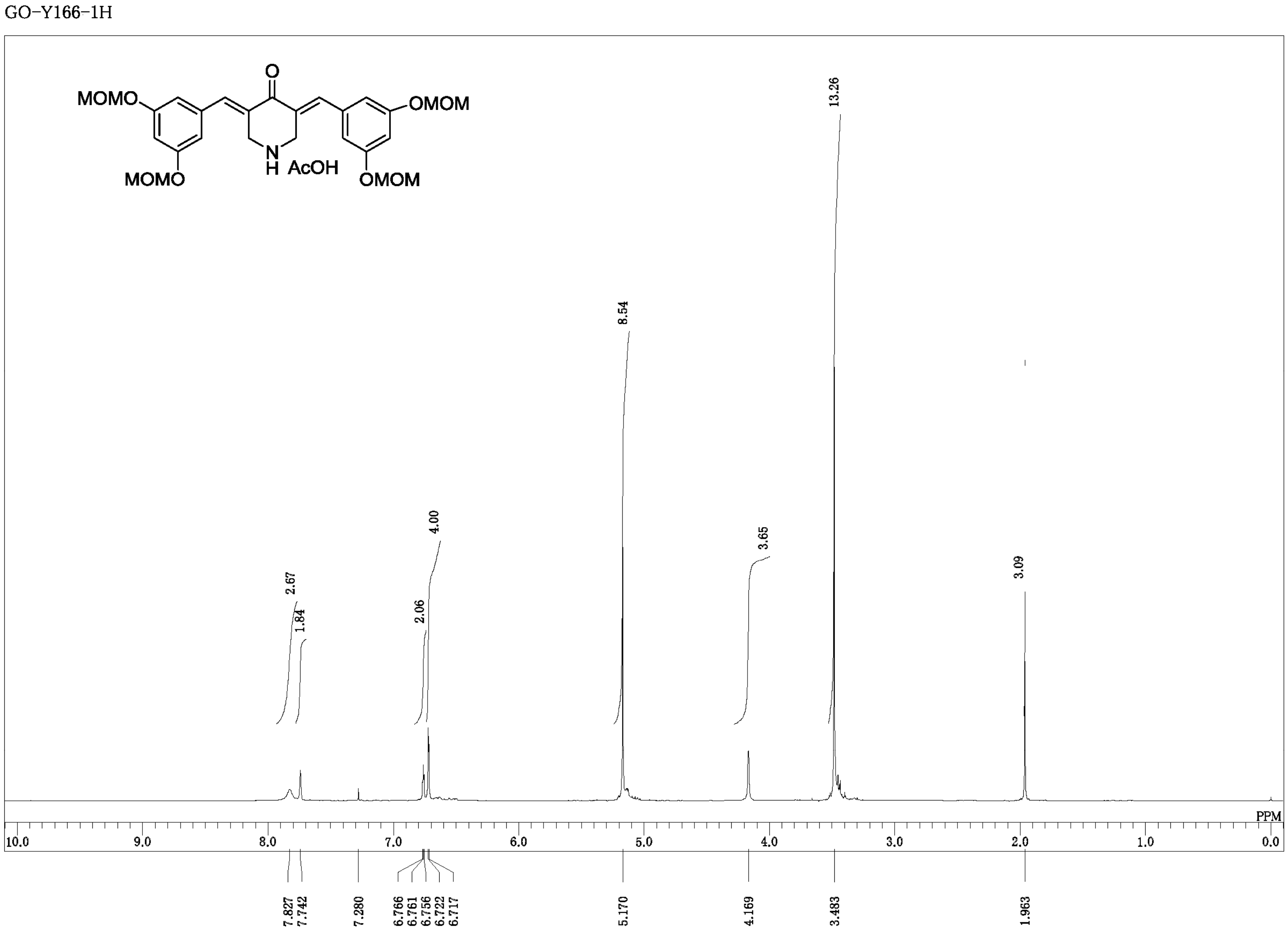

Figure S48. ^{1}H-NMR spectra of GO-Y166.

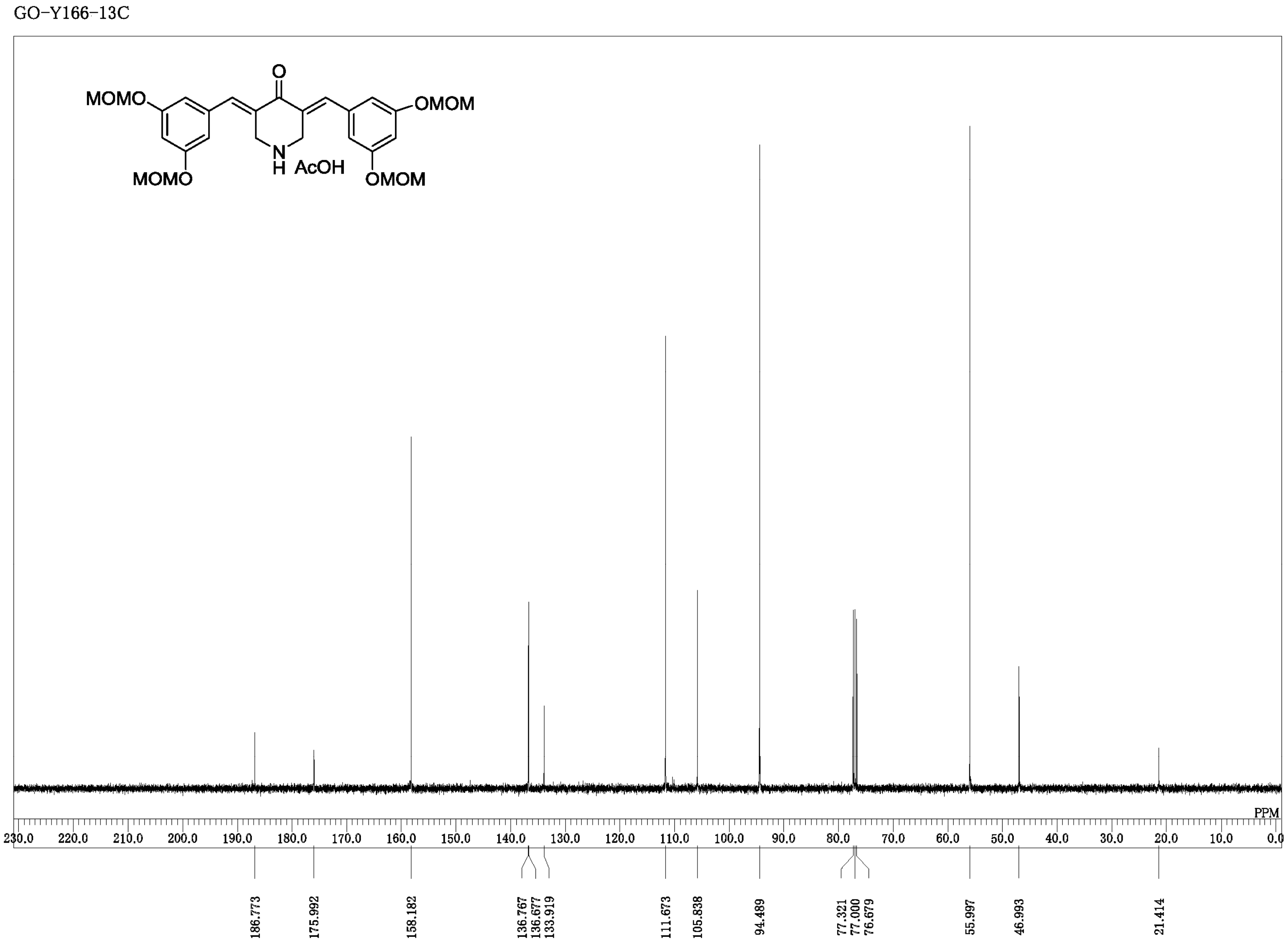

Figure S49. ^{13}C-NMR spectra of GO-Y166.

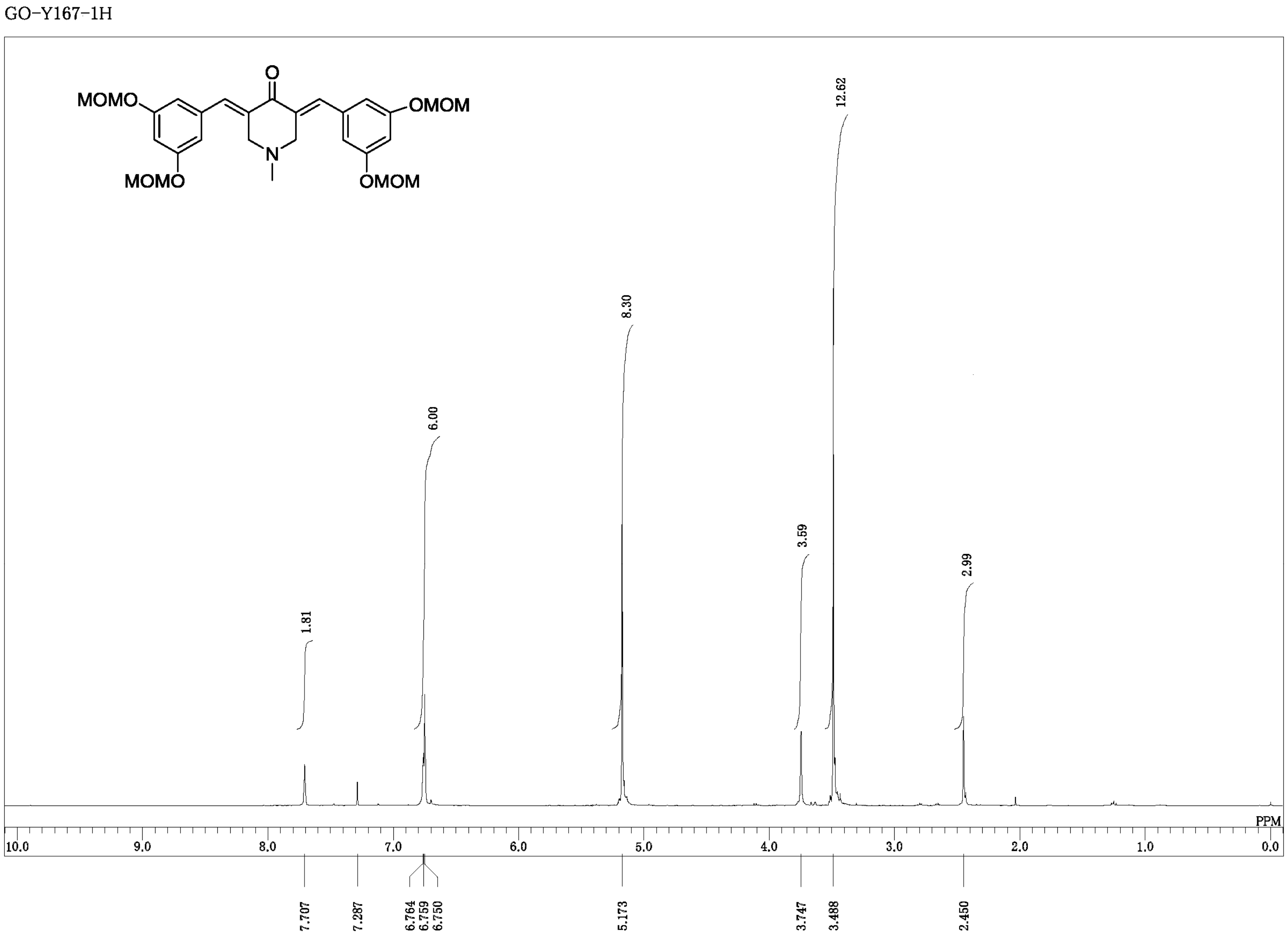

Figure S50. ^{1}H-NMR spectra of GO-Y167.

GO-Y167-13C

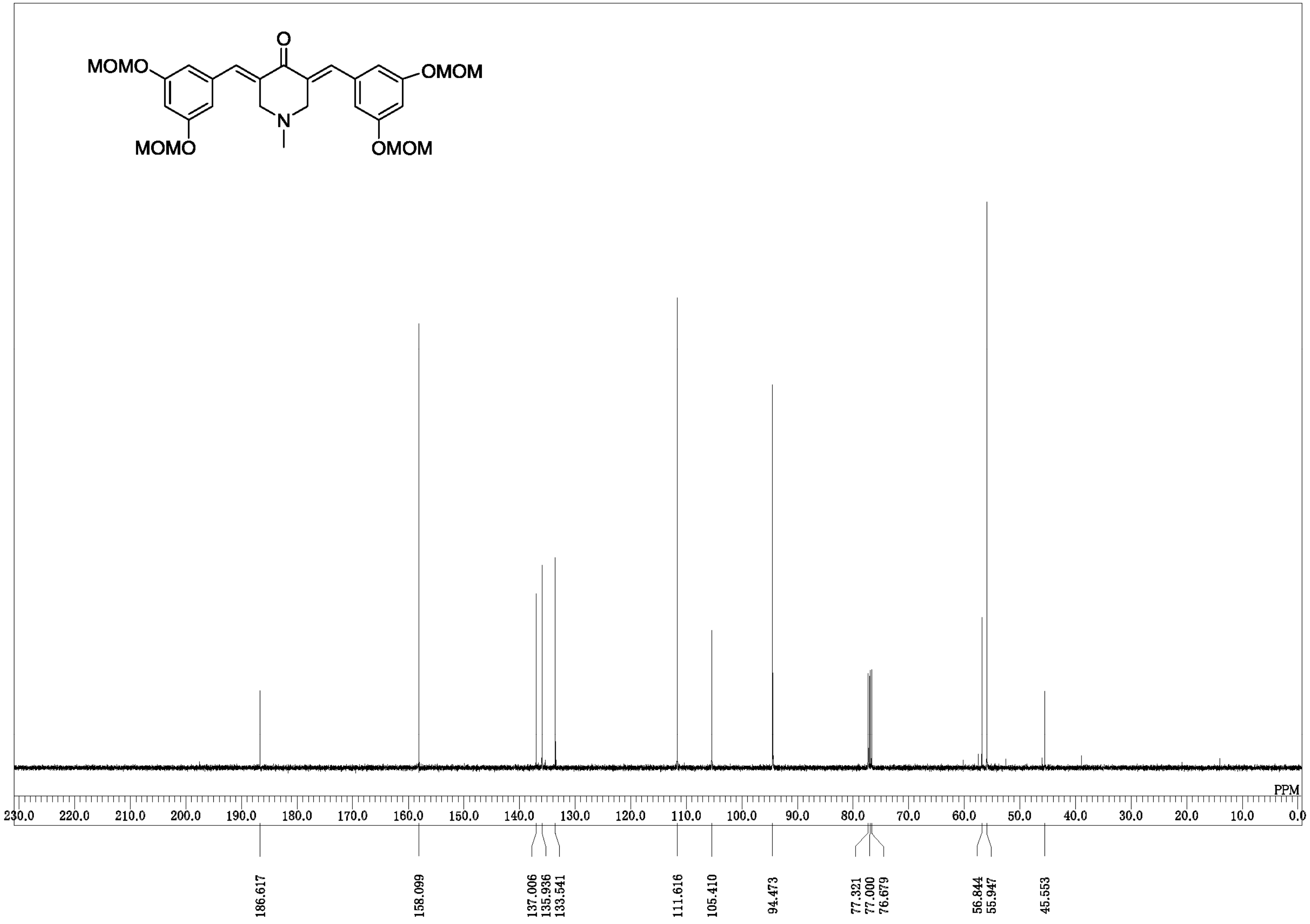

Figure S51. ^{13}C-NMR spectra of GO-Y167.

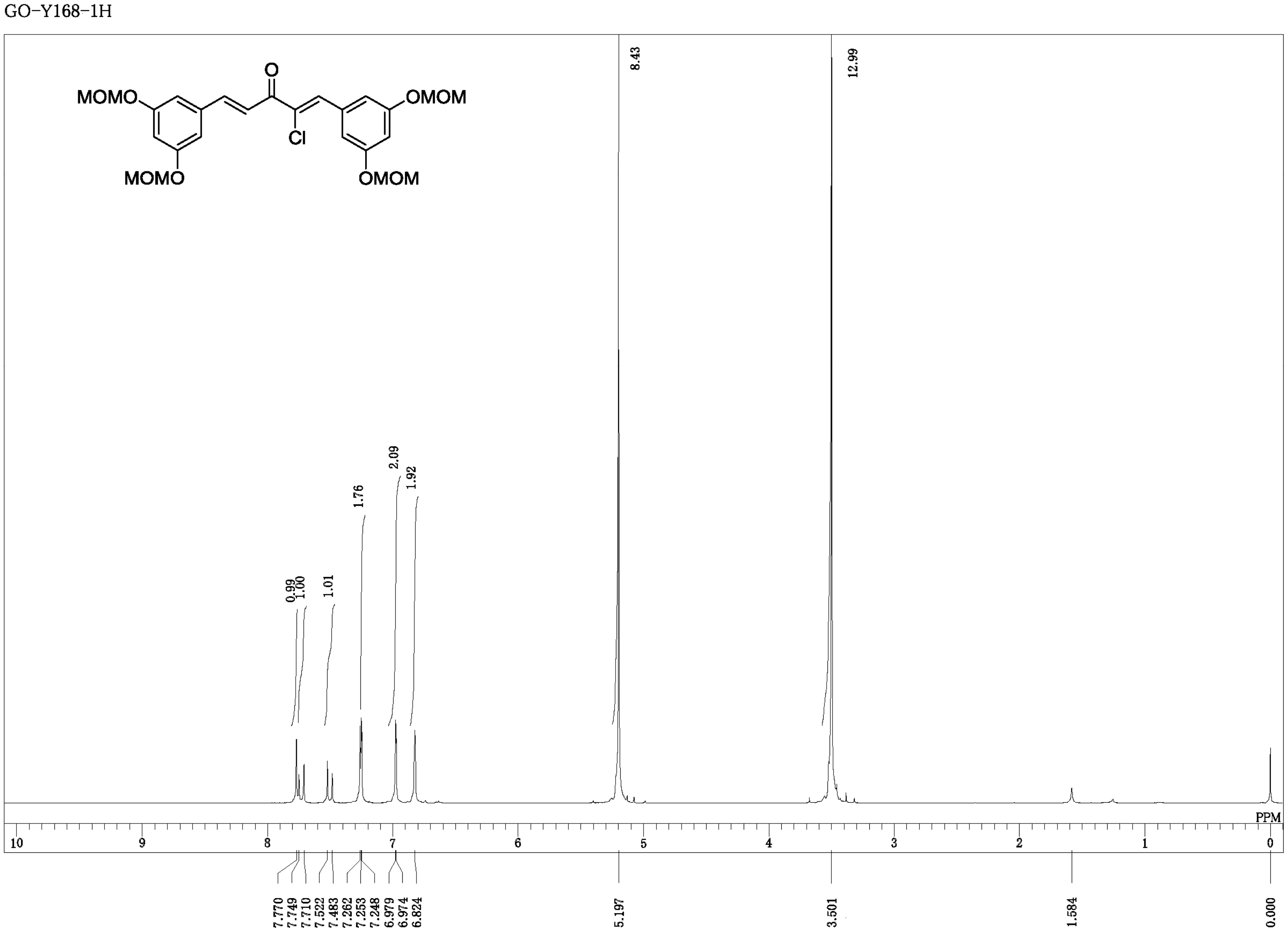

Figure S52. ^{1}H-NMR spectra of GO-Y168.

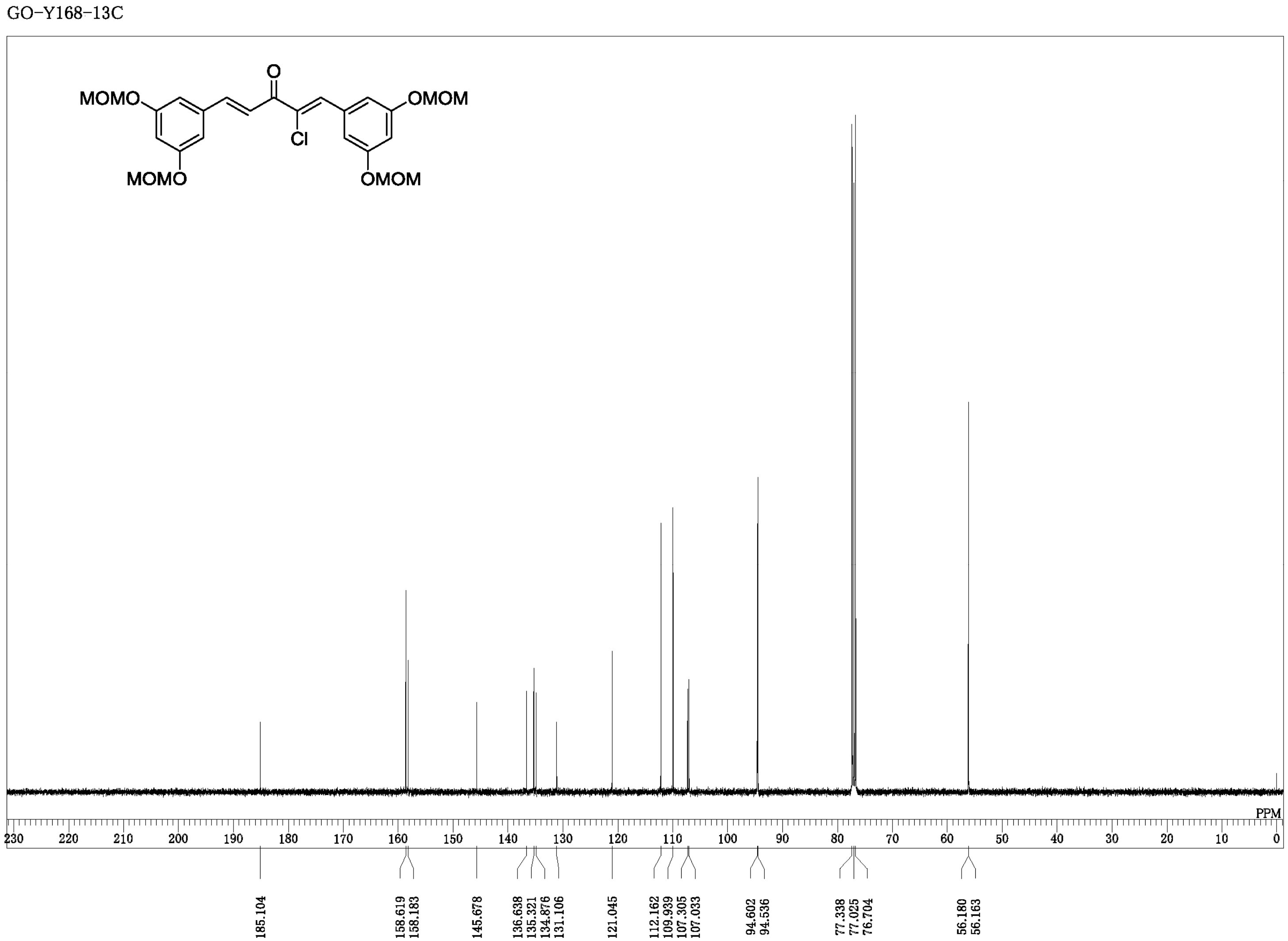

Figure S53. ^{13}C-NMR spectra of GO-Y168.

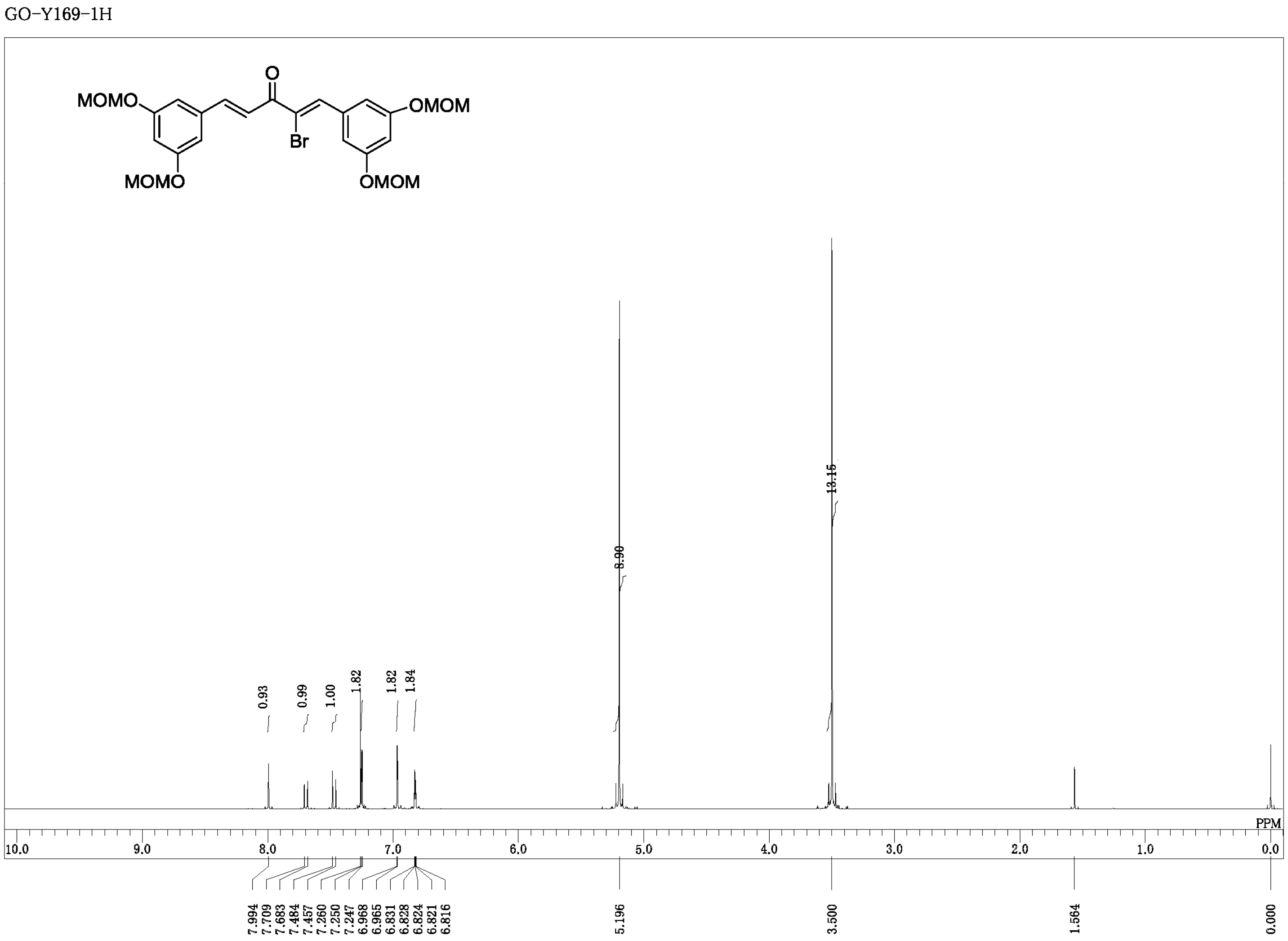

Figure S54. ^{1}H-NMR spectra of GO-Y169.

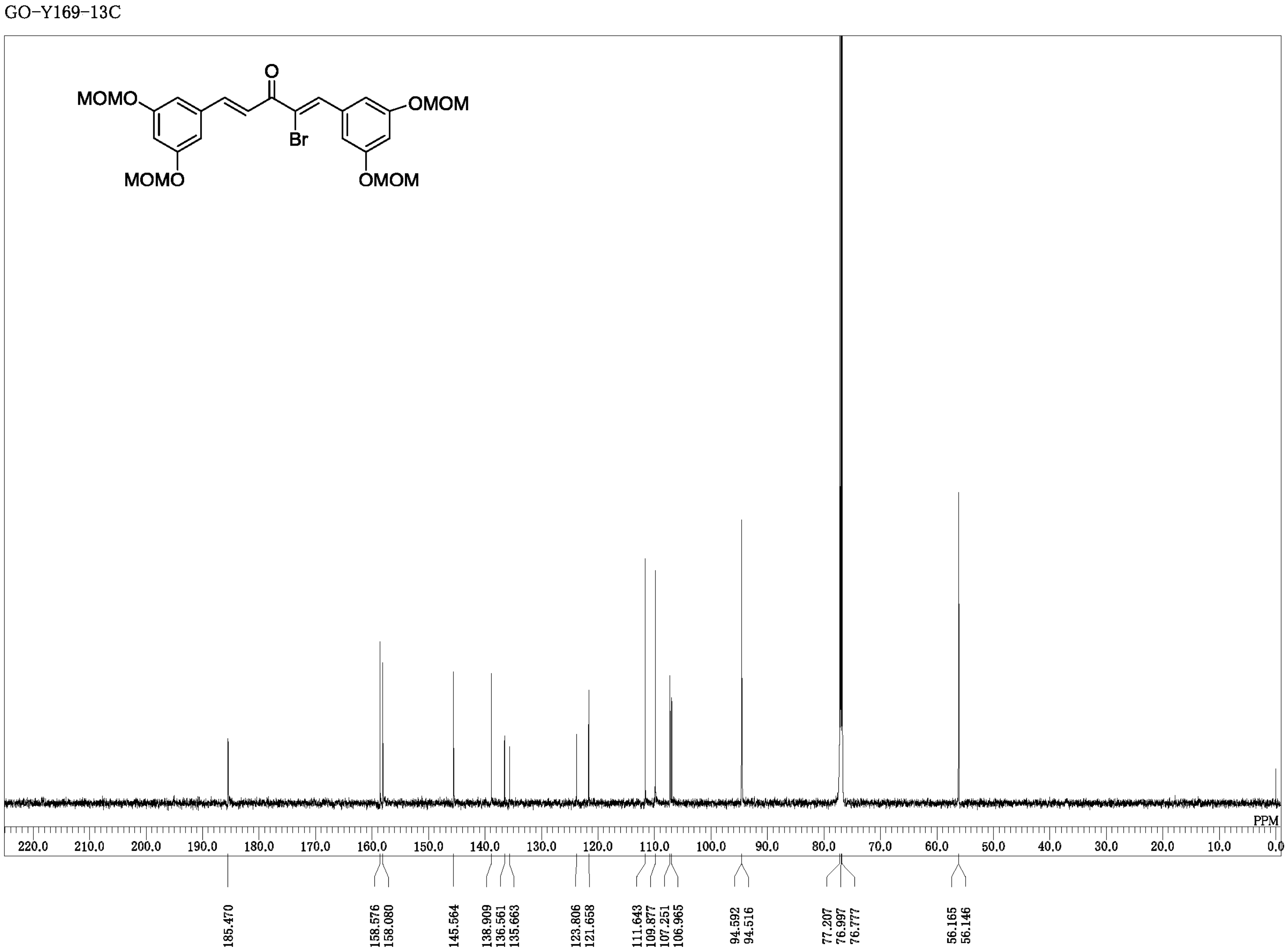

Figure S55. ^{13}C-NMR spectra of GO-Y169.

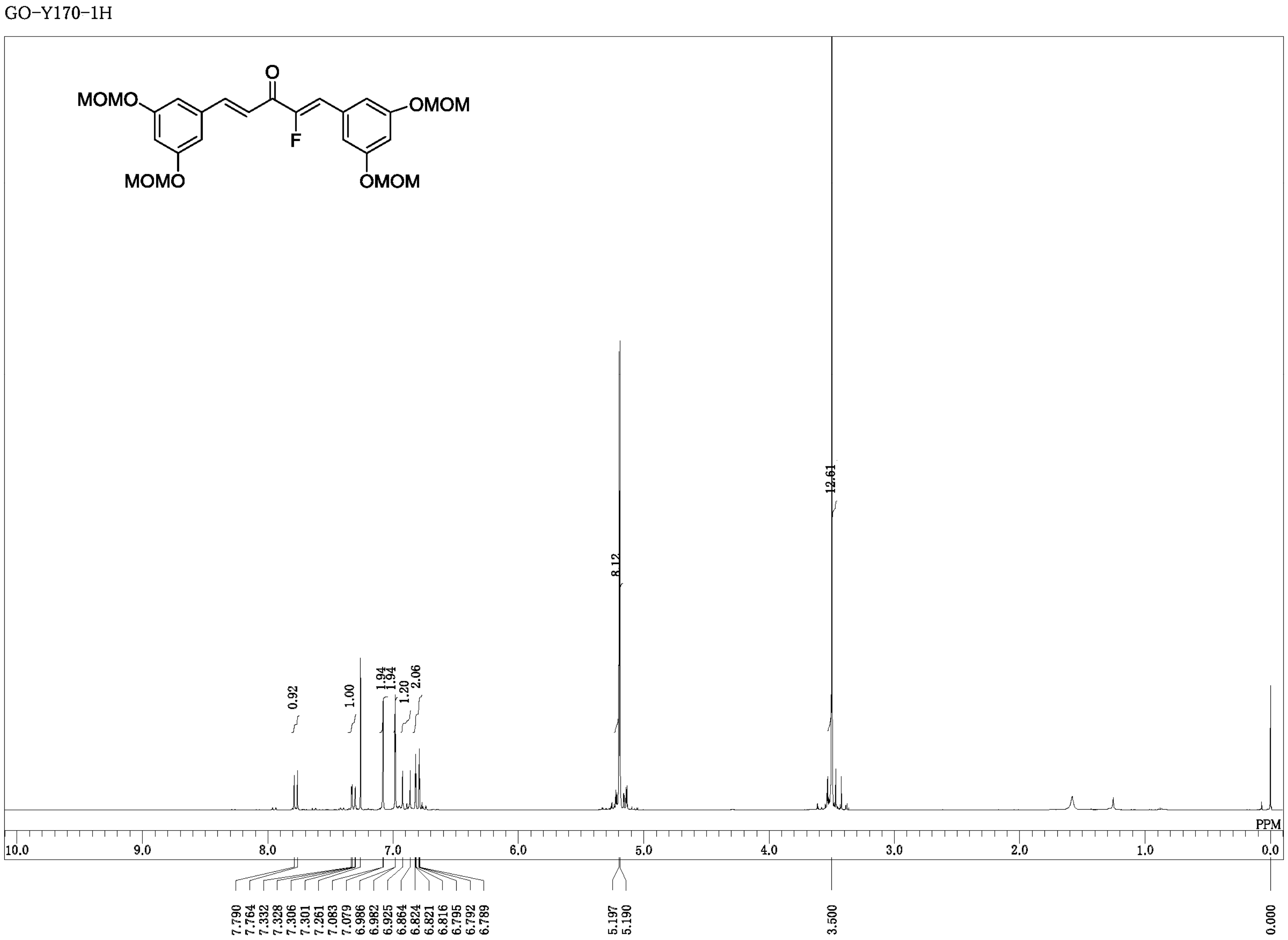

Figure S56. ^{1}H-NMR spectra of GO-Y170.

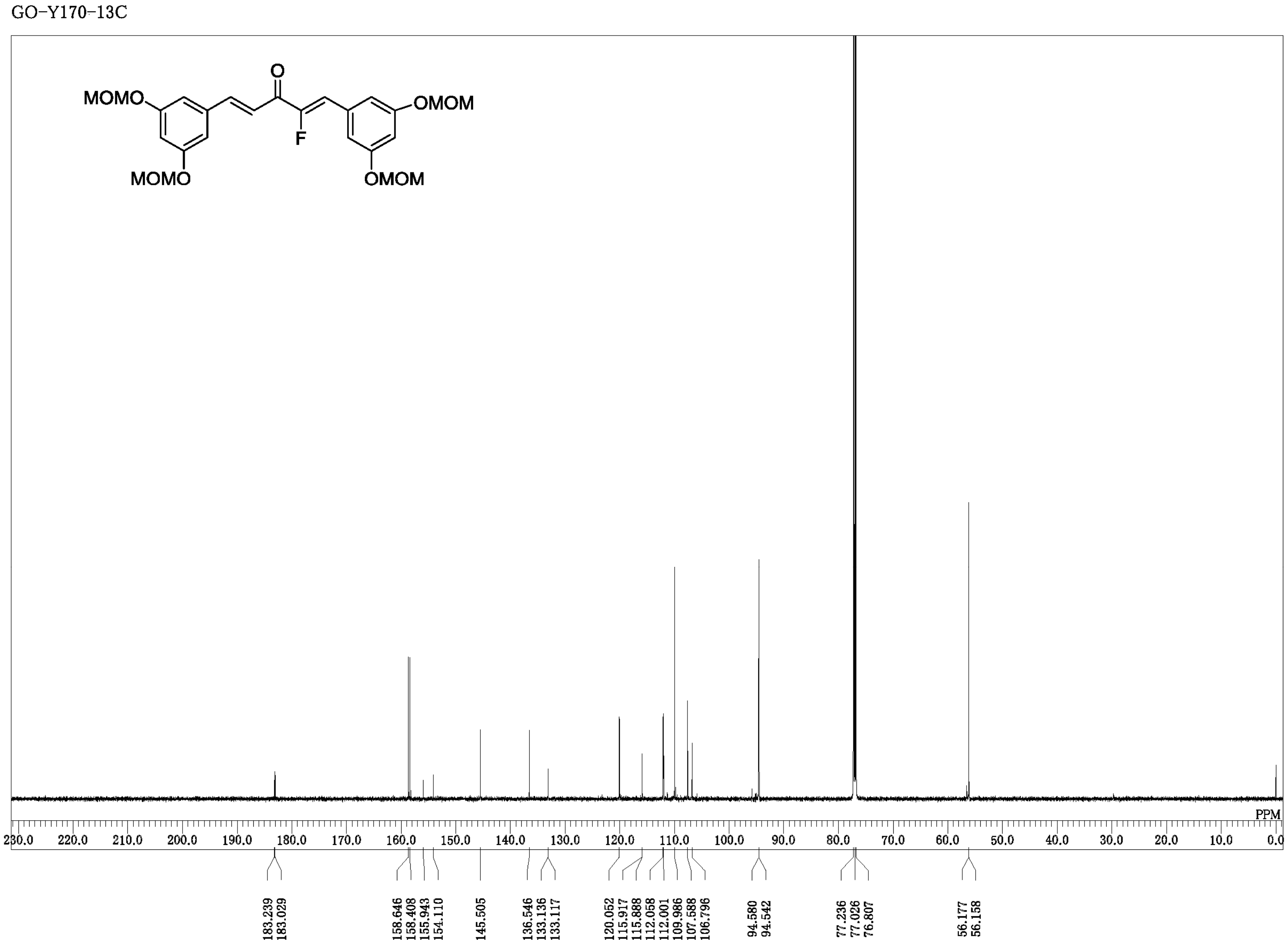

Figure S57. ^{13}C-NMR spectra of GO-Y170.

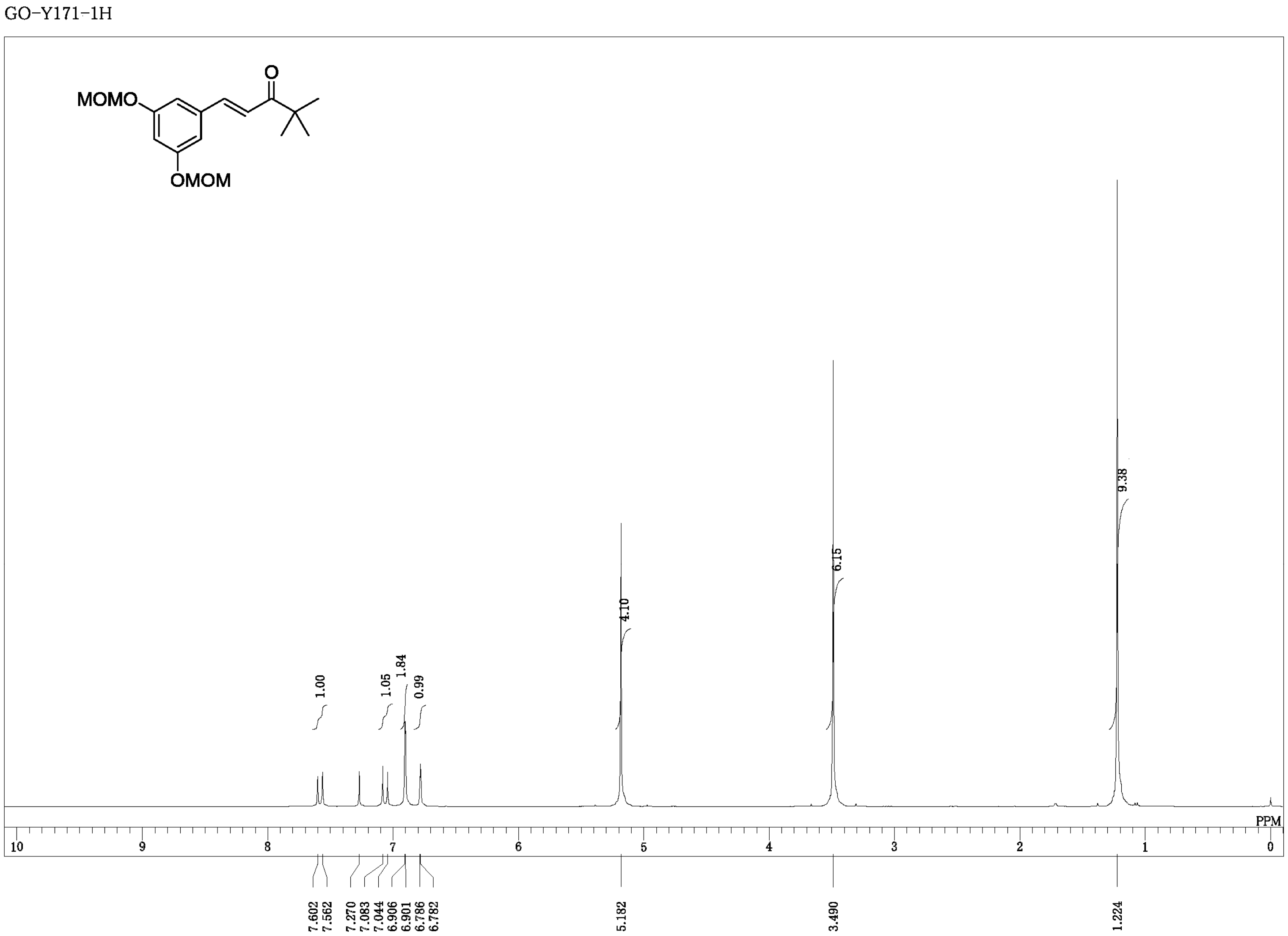

Figure S58. ^{1}H-NMR spectra of GO-Y171.

GO-Y171-13C

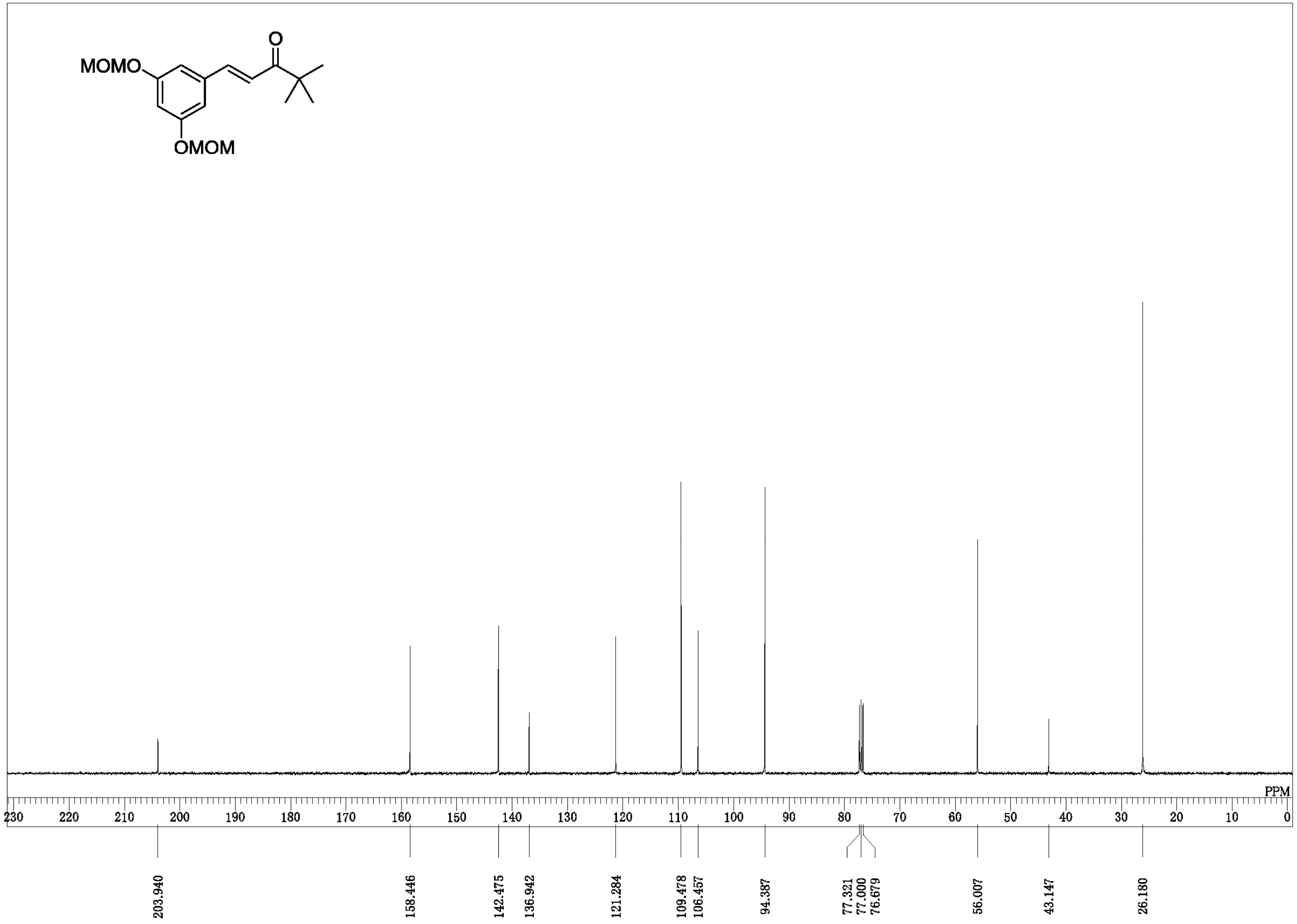

Figure S59. ^{13}C-NMR spectra of GO-Y171.

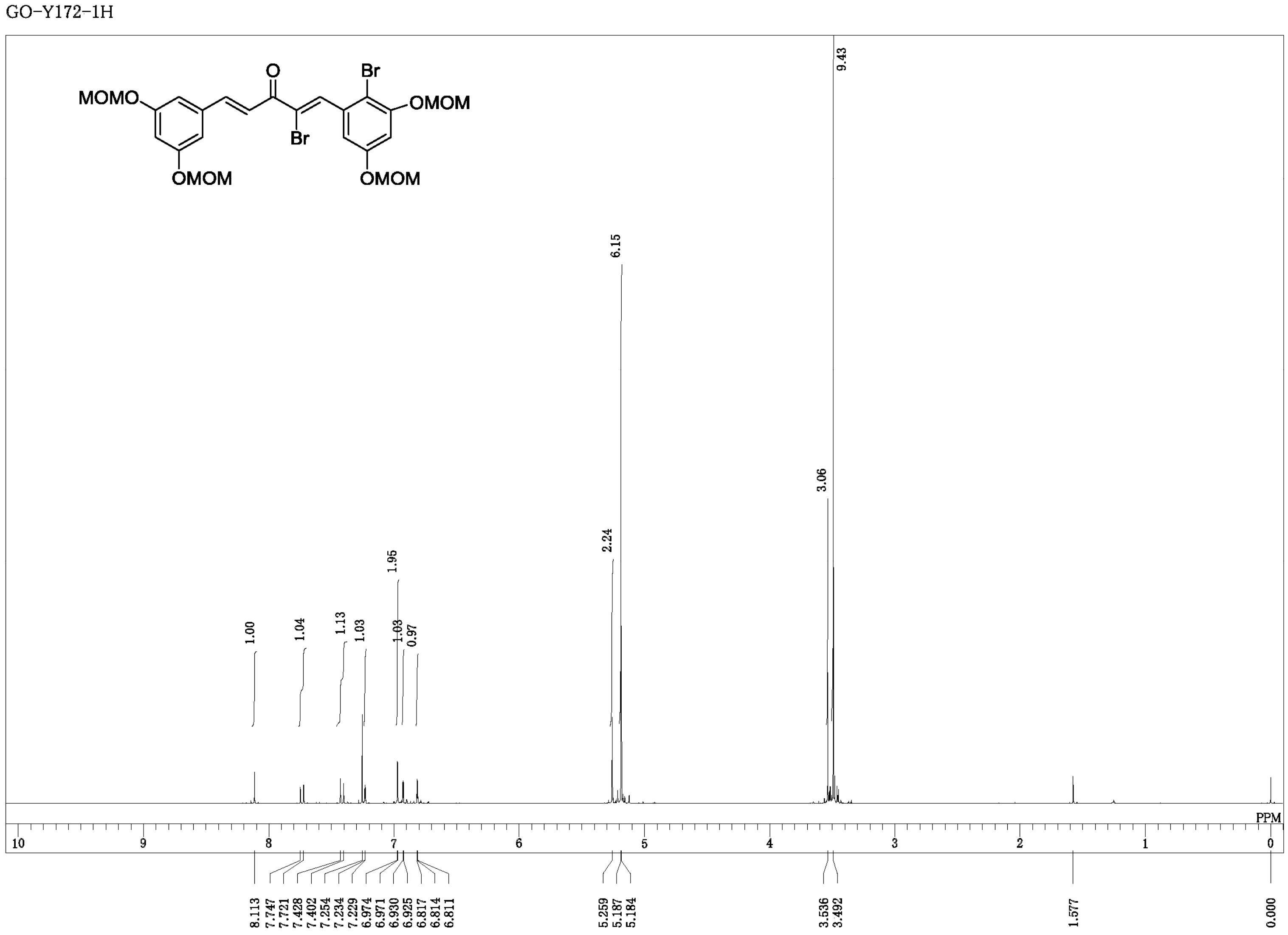

Figure S60. ^{1}H-NMR spectra of GO-Y172.

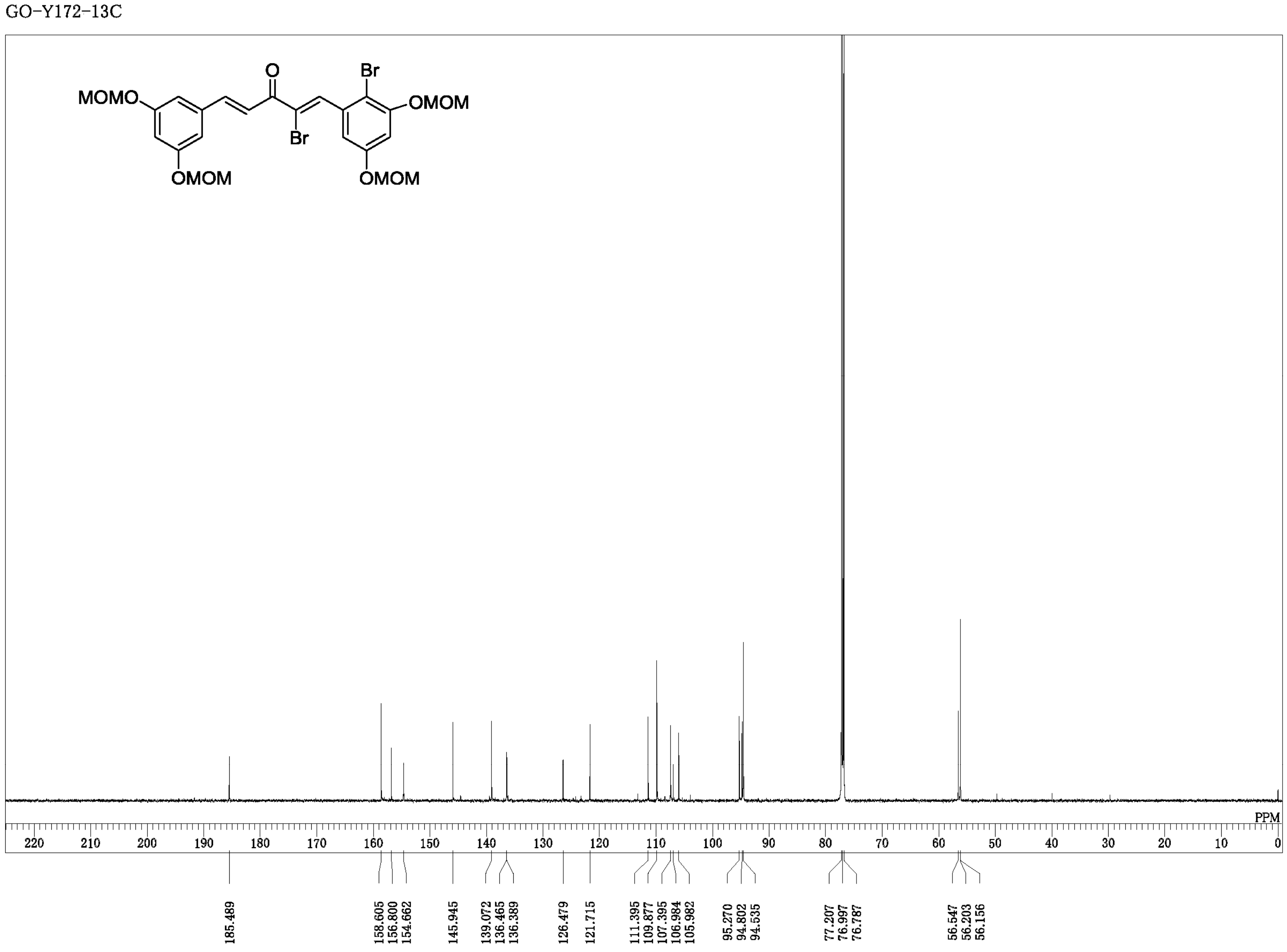

Figure S61. ^{13}C-NMR spectra of GO-Y172.

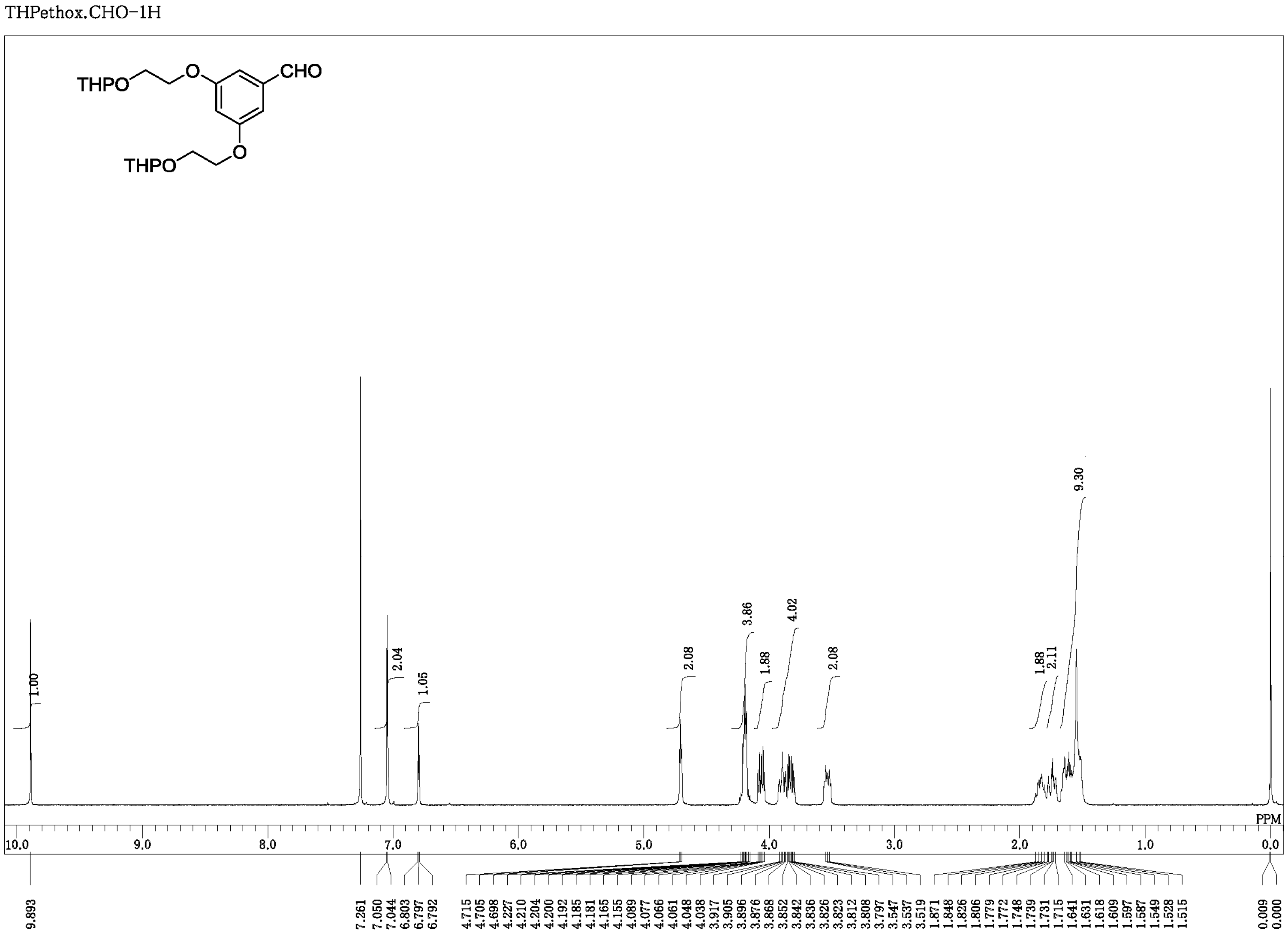

Figure S62. ^{1}H-NMR spectra of compound **S6**.

THPethox.CHO-13C

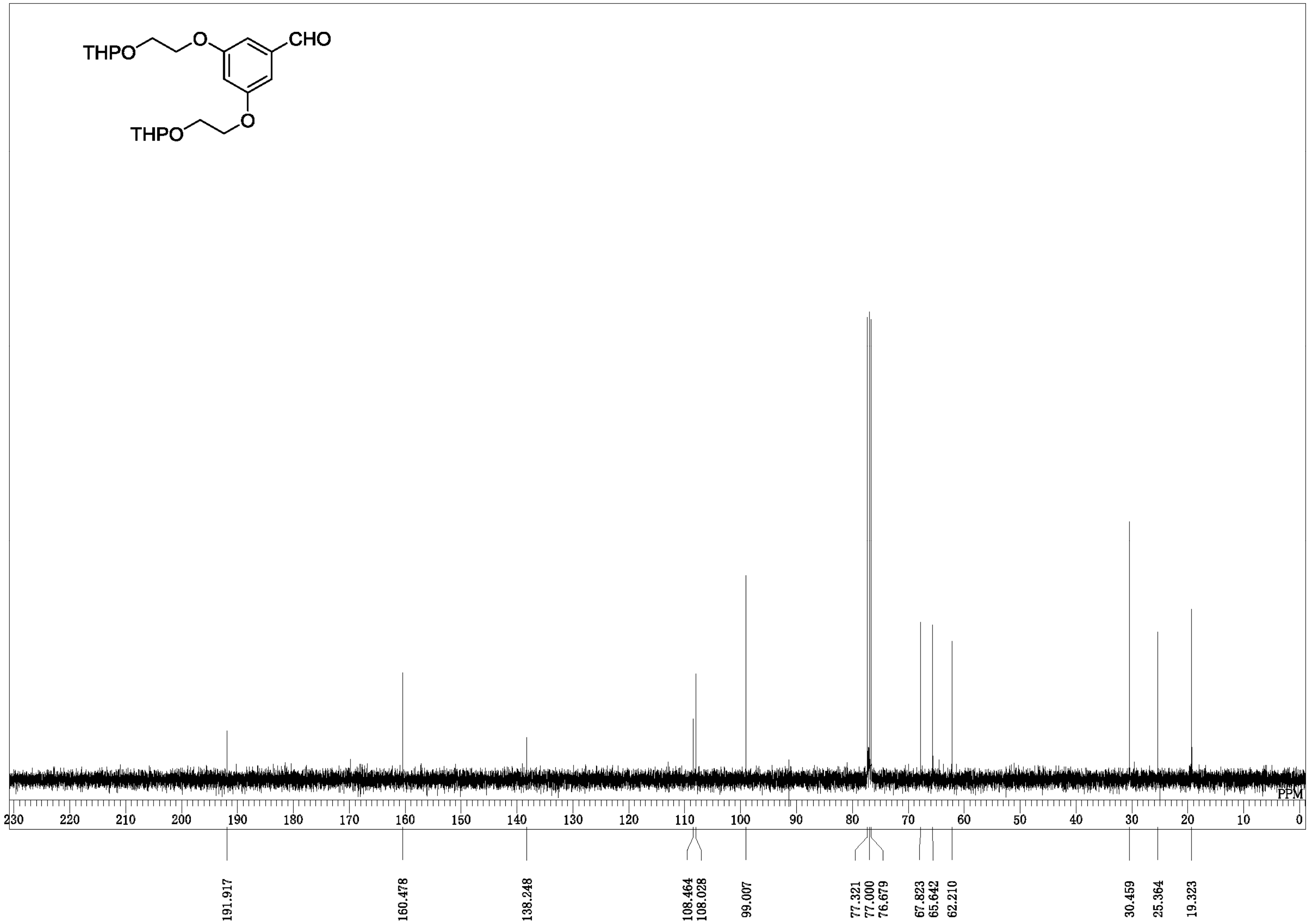

Figure S63. ^{13}C-NMR spectra of compound **S6**.

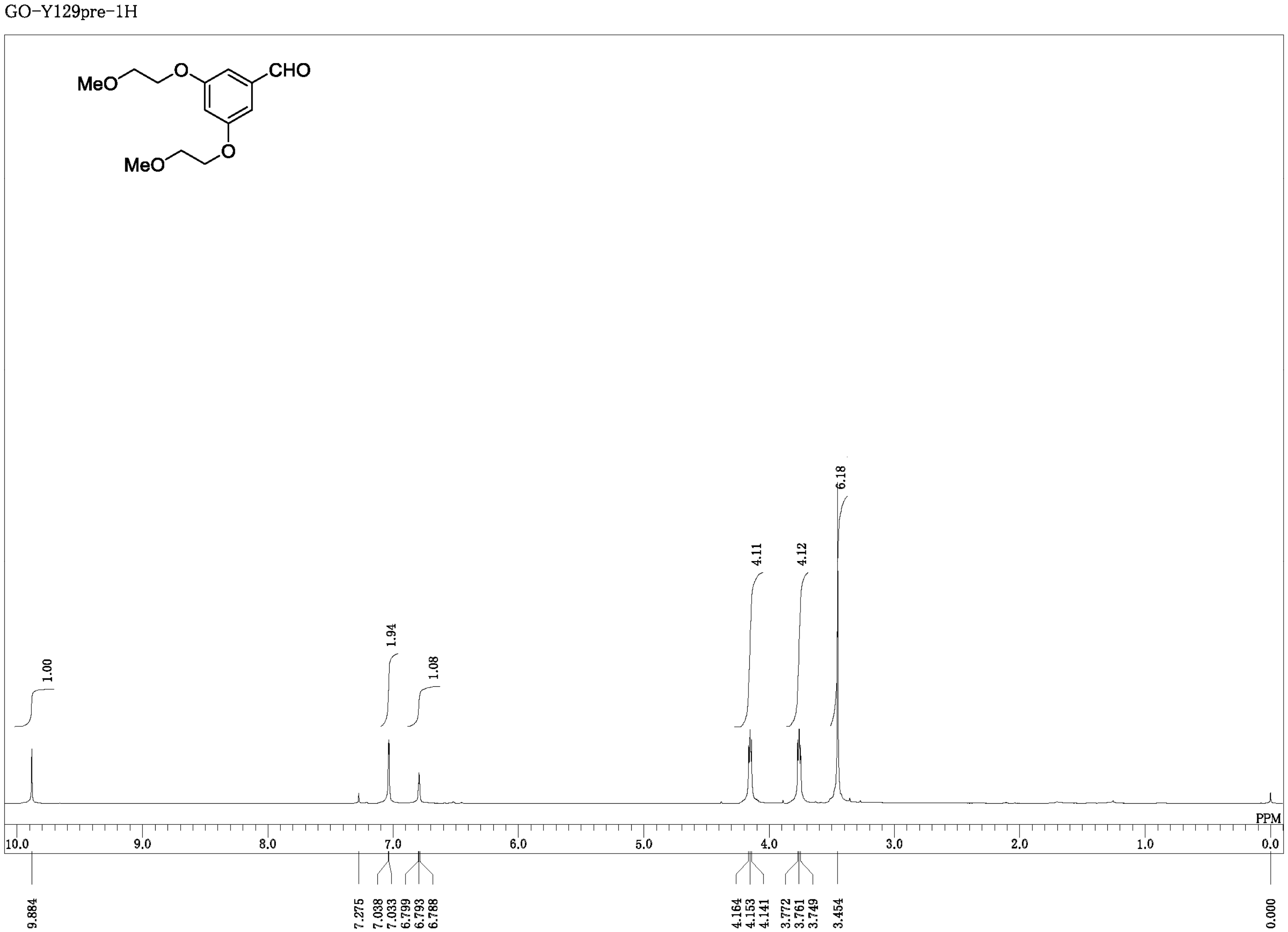

Figure S64. ^{1}H-NMR spectra of compound **S7**.

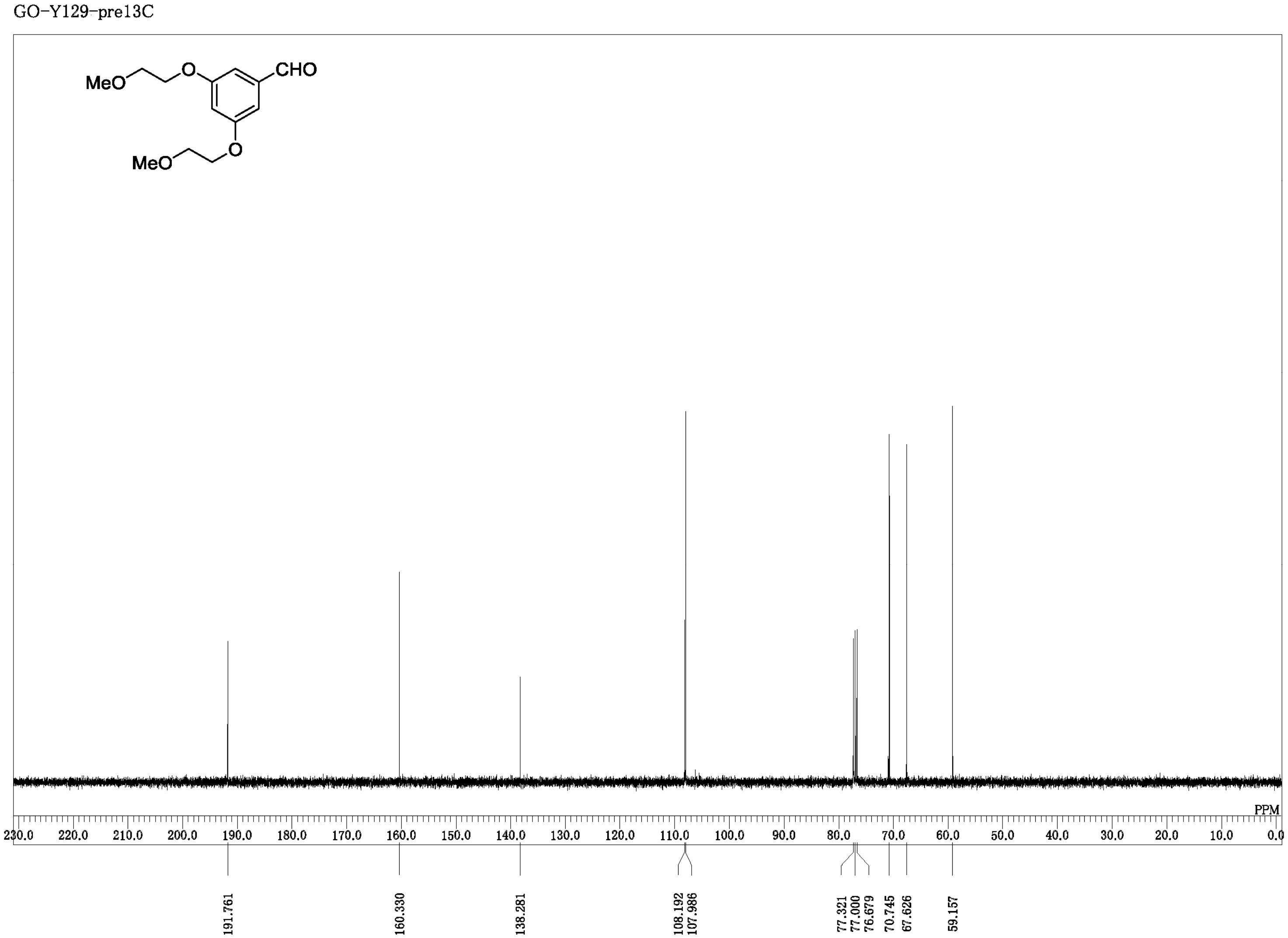

Figure S65. ^{13}C-NMR spectra of compound **S7**.

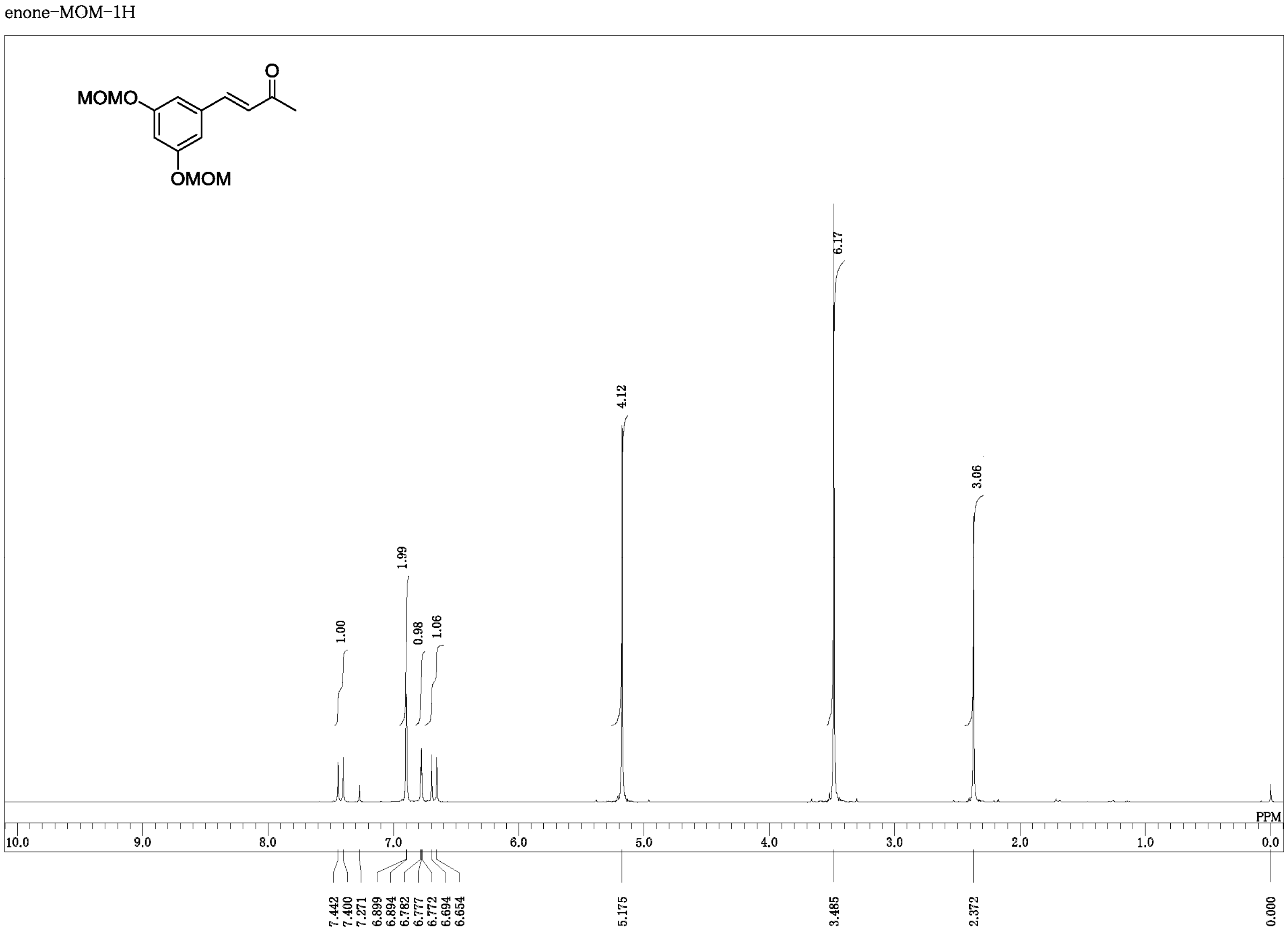

Figure S66. ^{1}H-NMR spectra of compound **6**.

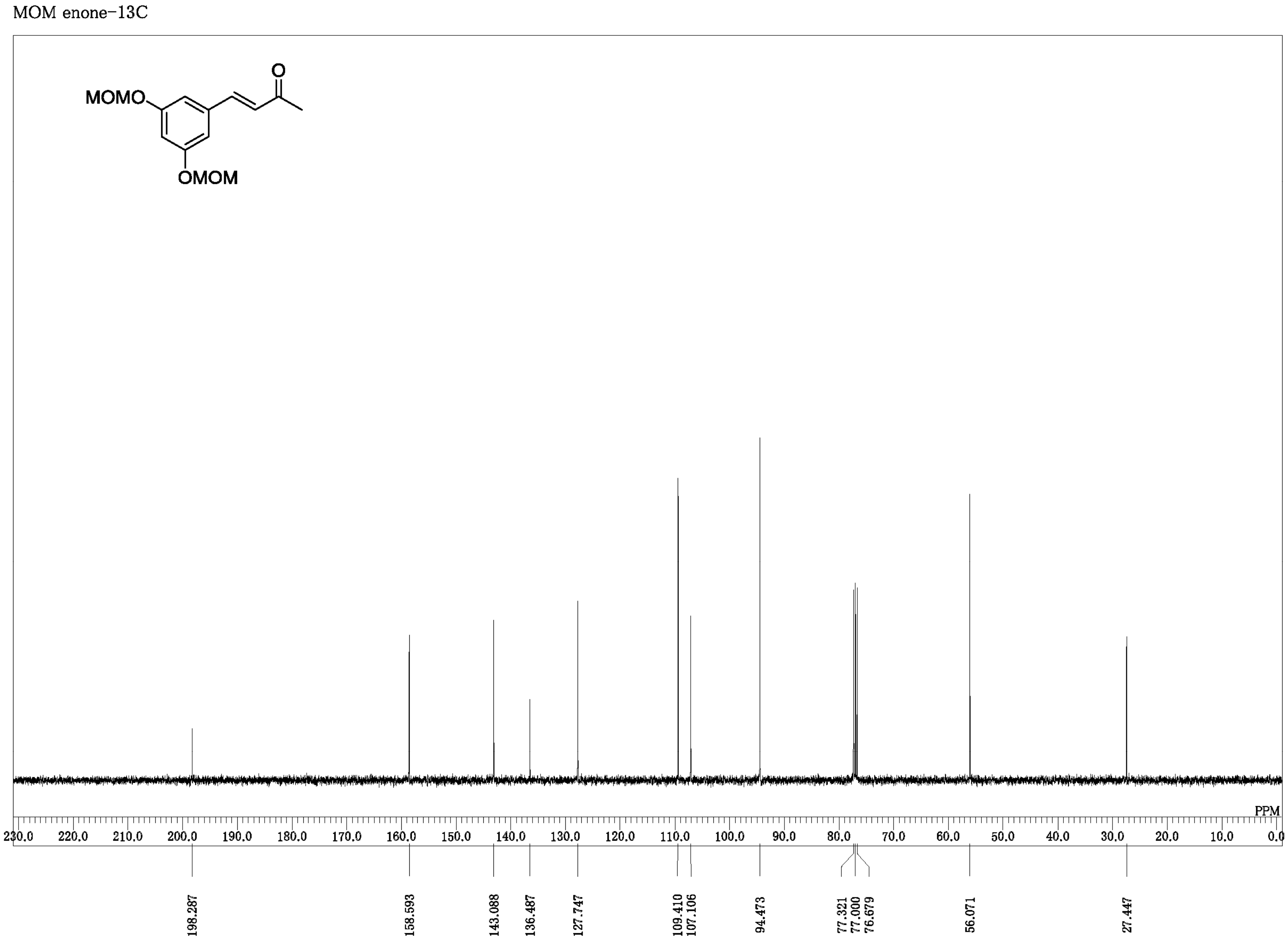

Figure S67. ^{13}C-NMR spectra of compound **6**.

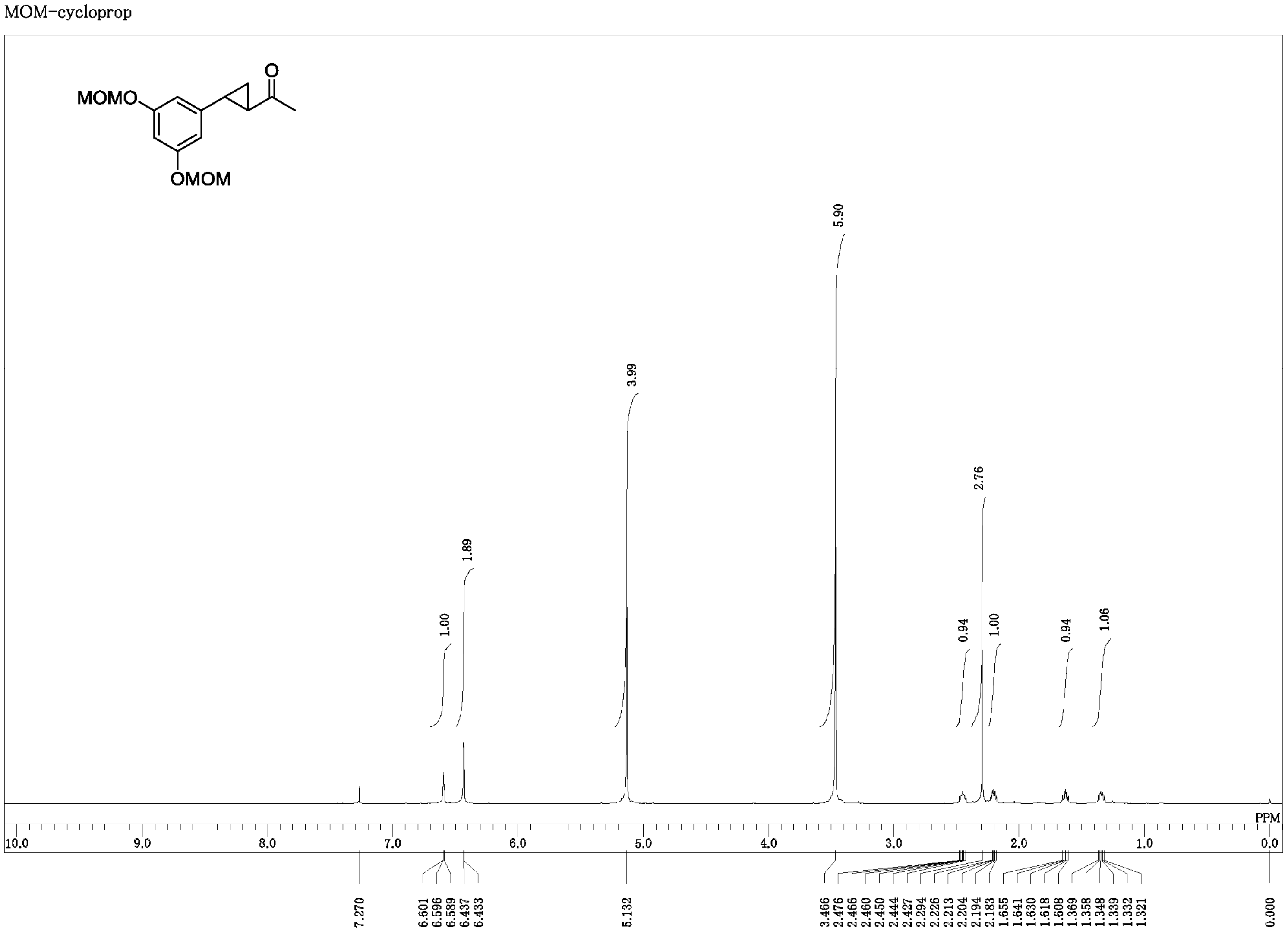

Figure S68. ^{1}H-NMR spectra of compound **7**.

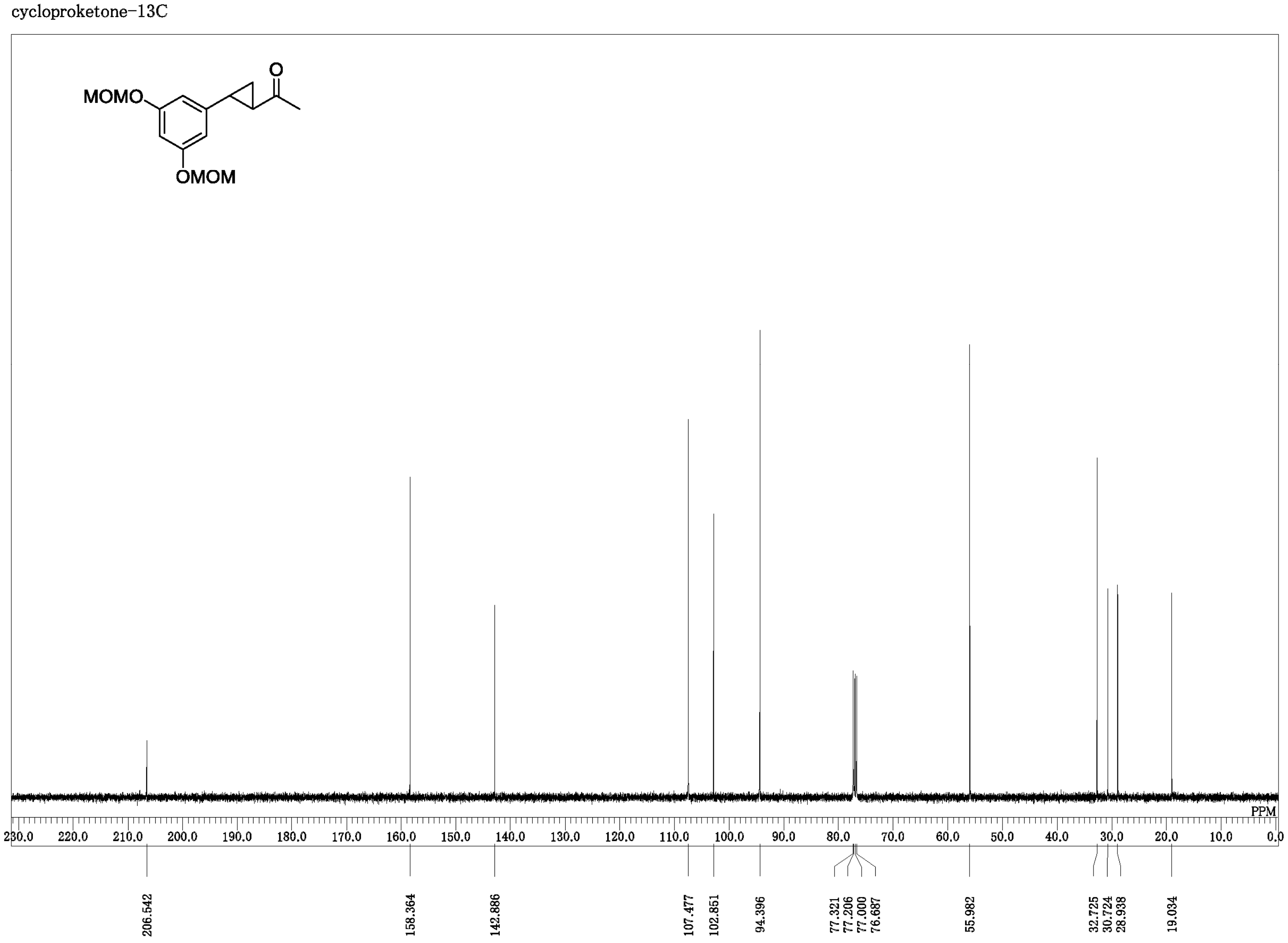

Figure S69. ^{13}C-NMR spectra of compound **7**.

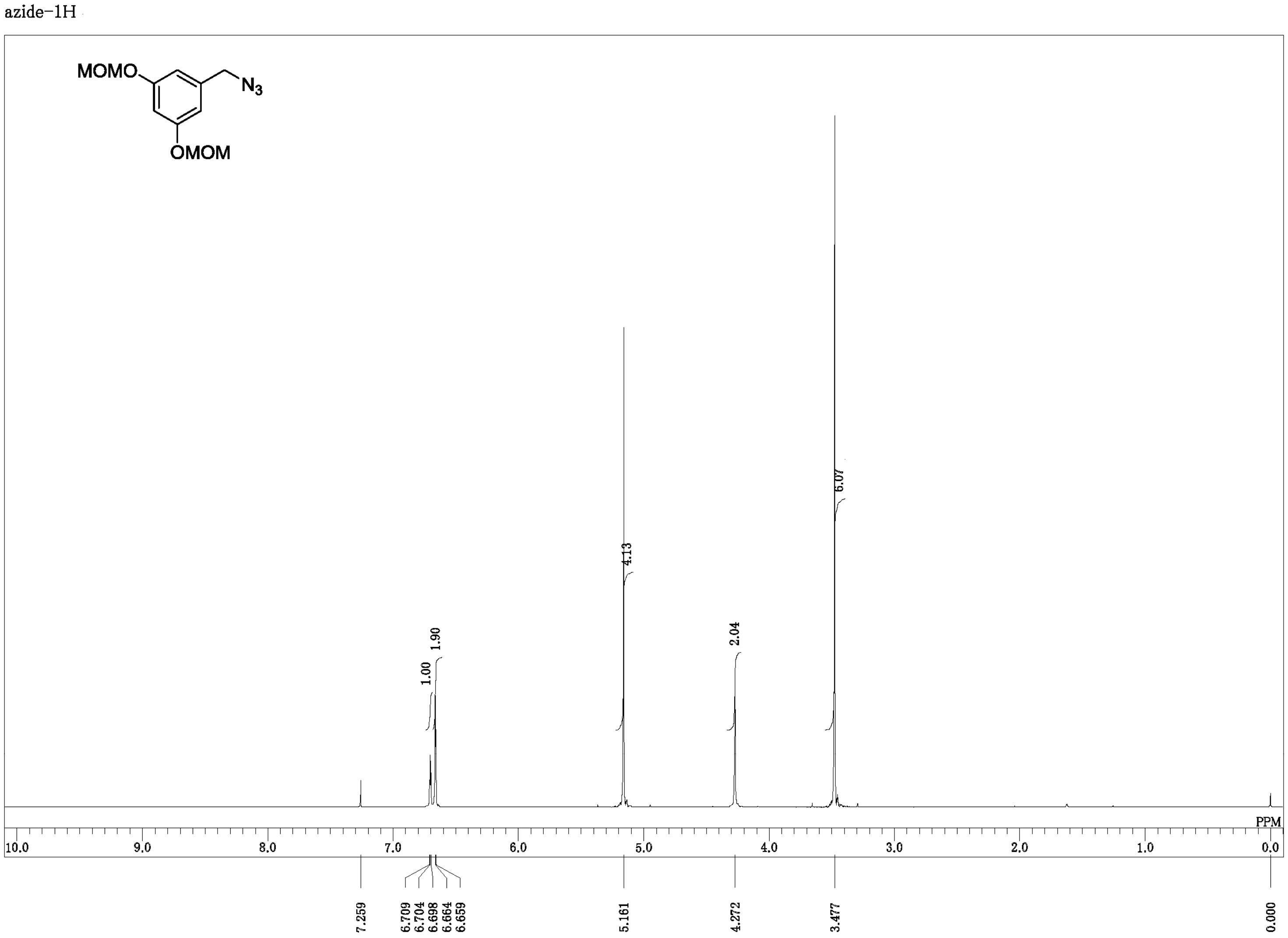

Figure S70. ^{1}H-NMR spectra of compound **S4**.

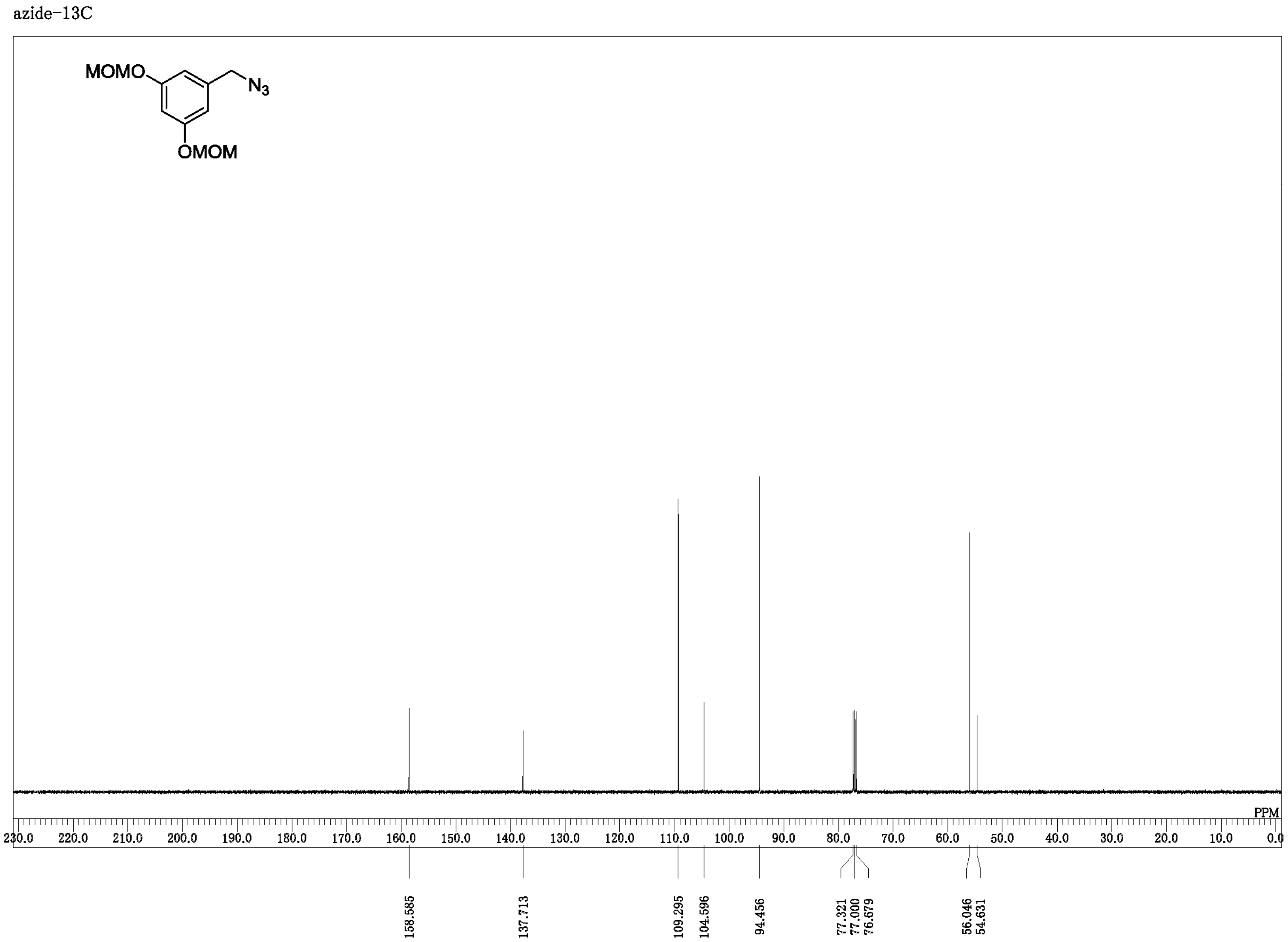

Figure S71. ^{13}C-NMR spectra of compound **S4**.

GO-Y172

NOESY (600 MHz, $CDCl_3$)

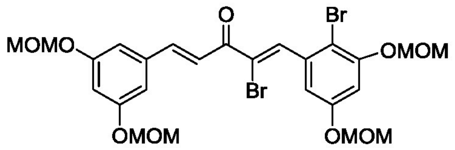

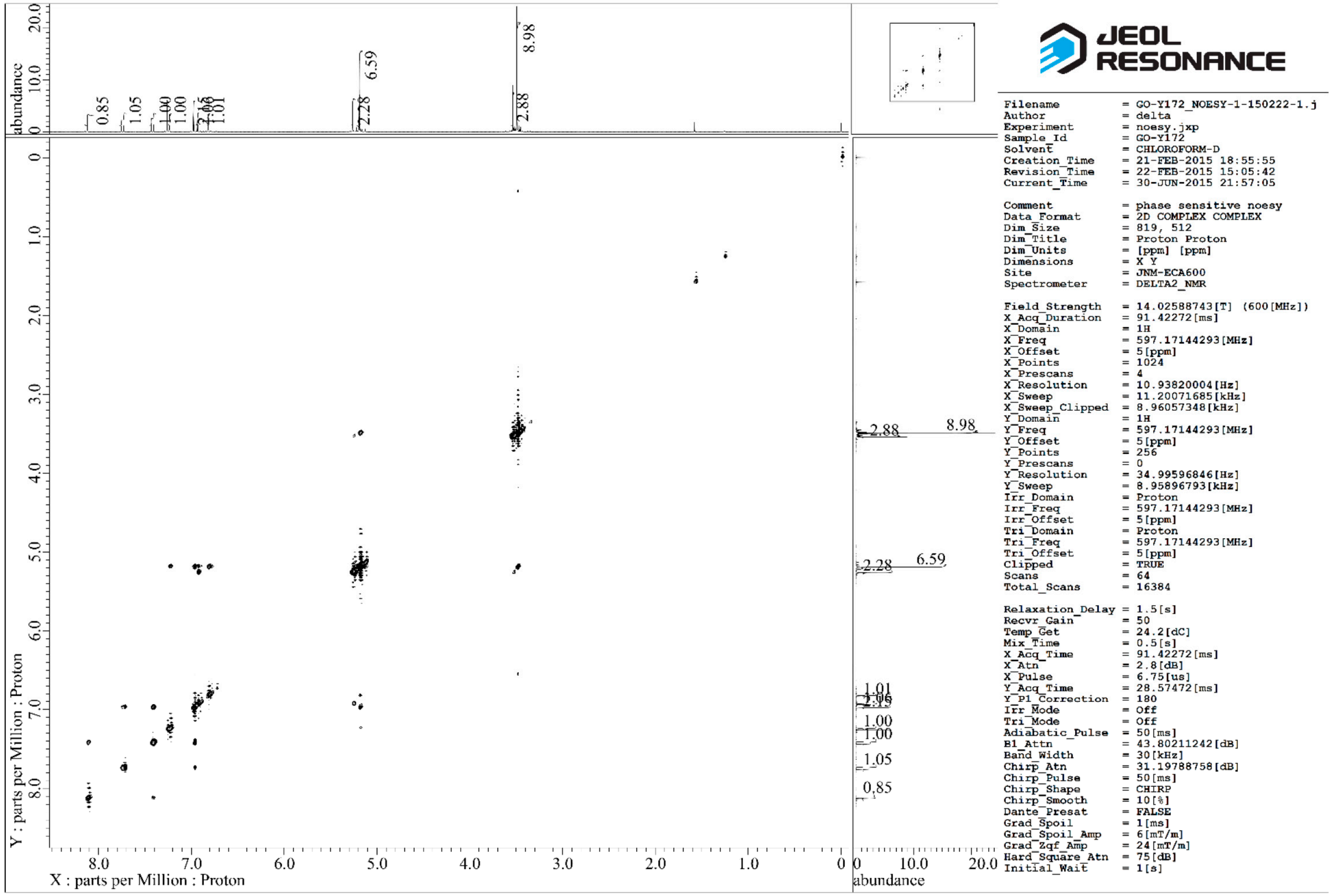

Figure S72. NOESY spectra of GO-Y172.
